# The E3 ligase HUWE1 inhibition as a therapeutic strategy to target MYC in multiple myeloma

**DOI:** 10.1038/s41388-020-1345-x

**Published:** 2020-06-10

**Authors:** Lisa J. Crawford, David C. Campbell, Jonathan J. Morgan, Michelle A. Lawson, Jennifer M. Down, Dharminder Chauhan, Roisin M. McAvera, Treen C. Morris, Claudia Hamilton, Aswini Krishnan, Krishnaraj Rajalingam, Andrew D. Chantry, Alexandra E. Irvine

**Affiliations:** 1 Patrick G Johnston Centre for Cancer Research, Queen’s University Belfast, Belfast, UK; 2 Department of Oncology and Metabolism, Sheffield Myeloma Research Team, Medical School, University of Sheffield, Sheffield, UK; 3 Department of Medical Oncology, The LeBow Institute for Myeloma Therapeutics and Jerome Lipper Myeloma Center, Dana Farber Cancer Institute, Harvard Medical School, Boston, MA USA; 4 Cell Biology Unit, University Medical Center Mainz, Mainz, Germany

**Keywords:** Myeloma, Ubiquitylation

## Abstract

Proteasome inhibitors have provided a significant advance in the treatment of multiple myeloma (MM). Consequently, there is increasing interest in developing strategies to target E3 ligases, de-ubiquitinases, and/or ubiquitin receptors within the ubiquitin proteasome pathway, with an aim to achieve more specificity and reduced side-effects. Previous studies have shown a role for the E3 ligase HUWE1 in modulating c-MYC, an oncogene frequently dysregulated in MM. Here we investigated HUWE1 in MM. We identified elevated expression of HUWE1 in MM compared with normal cells. Small molecule-mediated inhibition of HUWE1 resulted in growth arrest of MM cell lines without significantly effecting the growth of normal bone marrow cells, suggesting a favorable therapeutic index. Studies using a HUWE1 knockdown model showed similar growth inhibition. HUWE1 expression positively correlated with MYC expression in MM bone marrow cells and correspondingly, genetic knockdown and biochemical inhibition of HUWE1 reduced MYC expression in MM cell lines. Proteomic identification of HUWE1 substrates revealed a strong association of HUWE1 with metabolic processes in MM cells. Intracellular glutamine levels are decreased in the absence of HUWE1 and may contribute to MYC degradation. Finally, HUWE1 depletion in combination with lenalidomide resulted in synergistic anti-MM activity in both in vitro and in vivo models. Taken together, our data demonstrate an important role of HUWE1 in MM cell growth and provides preclinical rationale for therapeutic strategies targeting HUWE1 in MM.

## Introduction

Multiple Myeloma (MM) is a hematological neoplasm characterized by clonal proliferation of malignant plasma cells in the bone marrow (BM). The use of proteasome inhibitors (PIs) to disrupt the ubiquitin proteasome system (UPS) has provided a significant therapeutic advance, and highlights the importance of this pathway in MM [1]. Bortezomib, the first-in-class PI, is a widely used component of both frontline and relapsed MM therapy, while second generation PIs, carfilzomib and ixazomib, are approved for relapsed and refractory MM [1, 2]. Despite their success, acquired drug resistance and dose-limiting side effects can limit their clinical utility [3–6] and there is growing interest in targeting other components of the UPS.

The UPS plays a central role in protein homeostasis through the targeted destruction of misfolded proteins and regulatory proteins that control many critical cellular processes. Proteins are recruited for degradation by the process of ubiquitination, an enzymatic cascade involving three types of enzymes. An E1 enzyme activates ubiquitin and connects it to an E2 enzyme; an E3 ligase then mediates the transfer of ubiquitin to the target protein, thereby providing substrate specificity. The process can also be reversed by a group of proteases known as de-ubiquitinating enzymes (DUBs). Altered regulation of UPS enzymes has been associated with transformation and tumorigenesis [7, 8]. E3 ligases and DUBs, in particular, present ideal therapeutic candidates as they enable direct targeting of aberrant signaling pathways. A prime example of this is immunomodulatory drug (IMiD)-induced degradation of the transcription factors IKZF1 and IKZF3 through its binding to cereblon, the substrate receptor for the Cullin-4 E3 ligase complex [9]. Preclinical studies have highlighted a number of other DUBs and E3 ligases that present good therapeutic targets in MM [10–13].

HUWE1 is a large (482 kDa) HECT-domain E3 ligase that is involved in the regulation of key proteins such as c-MYC (MYC), p53 and MCL-1 (ref. [14]). Dysregulation of HUWE1 has frequently been associated with tumorigenesis and mutations in HUWE1 have recently been identified in MM patients [14, 15]. HUWE1 acts as both an oncogene and tumor suppressor depending on cell context. There are conflicting reports of the role of HUWE1 in regulating the oncogene c-MYC (MYC). In one model, HUWE1 regulates the stability of MYC through K48-linked ubiquitination and knockdown of HUWE1 leads to increased MYC and drives tumor progression [16]. Conversely, in other tumor types HUWE1 activates transcription of MYC through K63-linked ubiquitination and HUWE1 silencing or inhibition prevents MYC signaling and reduces tumor cell proliferation [17, 18]. These contradictory observations suggest that HUWE1 may play a cell-type and context-dependent role in MYC regulation. Pathological activation of MYC is a common feature of B-cell malignancies, including MM [19, 20]. High expression of HUWE1 has been demonstrated to specifically correlate with MYC-driven lymphomas [21], however the role of HUWE1 in regulating MYC in MM is unknown.

In this study, we show that HUWE1 is highly expressed in MM compared with healthy plasma cells. Using small molecule inhibitors and loss-of-function approaches, we demonstrate an essential role of HUWE1 in promoting MM growth in both in vitro and in vivo models. We further demonstrate that HUWE1 depletion induces MYC degradation in cell line models and patient-derived MM cells.

## Results

### HUWE1 is dysregulated in MM

**Fig. 1:**
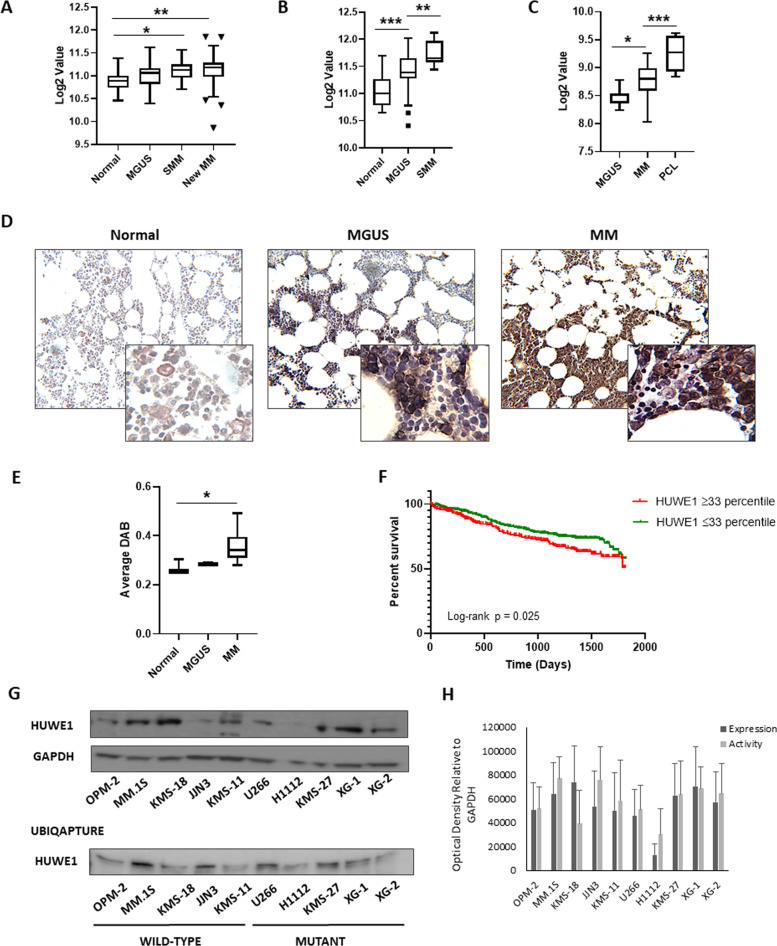
HUWE1 is dysregulated in multiple myeloma. *HUWE1* expression in CD138 + cells from (**a**) normal donors (*n* = 16), monoclonal gammopathy of undetermined significance (MGUS; *n* = 22), smoldering MM (SMM; *n* = 24), and newly diagnosed MM patients (*n* = 72); published dataset GSE6477, (**b**) normal donors (*n* = 22), MGUS (*n* = 44), and SMM (*n* = 14); published dataset GSE5900, and (**c**) MGUS (*n* = 7), MM (*n* = 39) and plasma cell leukemia (PCL; *n* = 6) patients; published dataset GSE2113. **d** Representative immunohistochemistry (IHC) images of dual staining for CD138 and HUWE1 in healthy donor bone marrow, patients with MGUS and MM patients. HUWE1 red staining, CD138 dark brown/black staining, blue counterstain (original magnification ×10 and ×20). **e** Nuclear HUWE1 DAB staining intensity from healthy donors (*n* = 3), MGUS (*n* = 3), and MM (*n* = 25). **f** Kaplan–Meier curves for 5 year overall survival relative to HUWE1 expression (CoMMpass dataset IA14 release); ≥33 percentile *n* = 244 (178 censored), ≤33 percentile *n* = 488 (378 censored); censored cases are represented by vertical ticks. **g** Top panel—HUWE1 expression in MM cell lines; Bottom panel—HUWE1 activity in MM cell lines (HUWE1 auto-ubiquitination is used as a read-out for catalytic activity). **h** Densitometry analysis of HUWE1 expression and activity in MM cell lines; *n* = 3. **a**, **b**, **c**, **e** One way ANOVA was used for statistical analysis; *p ≤ 0.05, **p ≤ 0.01, ***p ≤ 0.001.

Through the use of UPS topic-defined microarray analysis of MM cell lines, we previously identified a number of UPS-related genes, including *HUWE1*, with increased expression in MM compared with normal CD138 + plasma cells (Supplementary Fig. 1a, b) [22]. Further analysis of three independent gene expression datasets revealed a progressive increase in expression of *HUWE1* through a spectrum of plasma cell diseases (Fig. 1a–c). Similarly, immunohistochemical analysis of HUWE1 expression in BM from healthy donors (*n* = 3), patients with MGUS (*n* = 3), and MM (*n* = 25) demonstrated a significant increase in expression of HUWE1 in MM plasma cells, compared with normal controls (*p* = 0.014; Fig. 1d, e). Furthermore, analysis of *HUWE1* gene expression and overall survival of MM patients using data from the CoMMpass study, revealed that patients expressing higher levels of *HUWE1* exhibit a significantly reduced overall survival compared with those with lower expression (log-rank *p* = 0.025; Fig. 1f).

In addition to dysregulated expression of HUWE1 demonstrated here, mutations in HUWE1 have recently been identified in a small proportion of MM patients (5.7%) [15]. These are predominantly missense mutations distributed across the coding sequence and are significantly associated with the *t*(11;14) subgroup. A proportion (7%) of mutations reported in MM patients are found in the dimerization region of HUWE1, which based on previous studies are likely to lead to increased activity [23, 24], however, the effect of the majority of mutations has yet to be determined. Through analysis of publically available MM cell line data (www.keatslab.org) we identified cell lines with HUWE1 mutations and subsequently confirmed HUWE1 mutations in five MM cell lines (U266, XG-1, XG-2, KMS-27, and H1112) by Sanger sequencing (Supplementary Fig. 1c). In line with MM patient data, HUWE1 mutations were predominantly found in cell lines with a *t*(11;14) translocation and are distributed in a similar manner (Supplementary Fig. 1c-d). HUWE1 protein expression in mutant cell lines was compared with expression in five MM cell lines with wild type (WT) HUWE1 (JJN3, MM.1S, ANBL-6, KMS-18, OPM-2). HUWE1 protein expression was significantly lower in the H1112 cell line (*p* = 0.002), which carries a splice-site mutation, compared with HUWE1 wild-type cell lines. However, UbiQapture-Q assays demonstrated HUWE1 catalytic activity, as measured by auto-ubiquitination activity, in all cell lines including H1112 (Fig. 1g, h).

### HUWE1 inhibition or depletion decreases cell viability and induces cell cycle arrest in MM cells

**Fig. 2:**
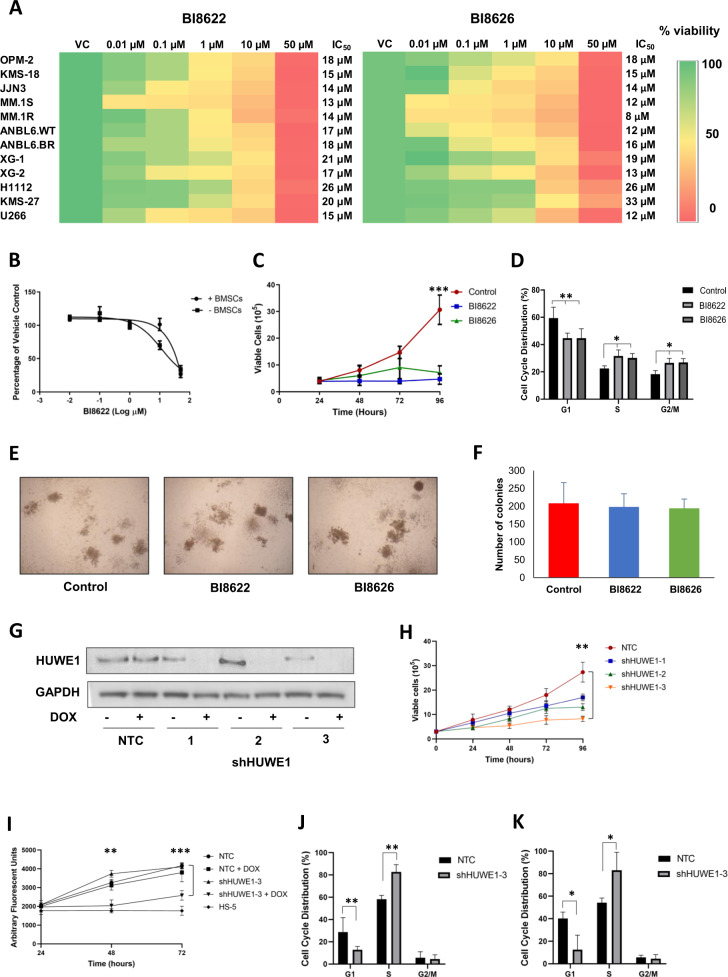
Inhibition or knockdown of HUWE1 halts MM cell proliferation. **a** 24 h dose response of MM cell lines to BI8622 (left panel) and BI8626 (right panel). **b** Dose response of JJN3 cells to BI8622 when cultured alone or in co-culture with bone marrow stromal cells (BMSCs) for 24 h. **c** Growth curve of JJN3 cells cultured in the presence of 14 μM BI8622 or BI8626; DMSO treated cells were used as a vehicle control. **d** Cell cycle distribution of JJN3 cells cultured in the presence of 15 μM BI8622 or BI8626 for 24 h. **e** Representative colony growth of mononuclear cells from healthy donors cultured for 14 days in semi-solid methylcellulose based media in the presence of 15 µM BI8622 or BI8626; DMSO treated cells were used as a vehicle control. **f** Quantification of colonies after 14 days. **g** JJN3 cells expressing doxycycline‐inducible shRNAs targeting HUWE1 or a non-targeting control (NTC) shRNA were treated with 1 μg/ml doxycycline (dox). Expression of HUWE1 was analyzed by Western blotting 72 h post-dox treatment. **h** Growth curve of JJN3 cells expressing doxycycline‐inducible shRNAs or NTC shRNA; 72 h post-dox treatment cells were seeded at 3 × 10^5^/ml and growth was monitored over 96 h. **i** Growth curve of JJN3 cells expressing NTC shRNA or shHUWE1-3 cultured alone or in co-culture with HS-5 stromal cells. **j** & **k** Cell cycle distribution of JJN3 cells expressing NTC shRNA or shHUWE1-3 72 h (**j**) or 1 week (**k**) post-dox treatment. *n* ≥ 3 for all experiments; paired *t*-test was used for statistical analysis; *p ≤ 0.05, **p ≤ 0.01, ***p ≤ 0.001.

To determine whether HUWE1 has any functional significance in MM, we first analyzed the effect of small molecule inhibition or shRNA knockdown of HUWE1 on the growth and survival of MM cell lines. HUWE1 inhibitors BI8622 and BI8626 have been previously identified and characterized [16, 25]. Inhibition of HUWE1 auto-ubiquitination confirmed on-target activity of the inhibitors (Supplementary Fig. 2a). Both inhibitors reduce the viability of a panel of MM cell lines, including those resistant to dexamethasone (MM.1R) and bortezomib (ANBL6-BR) in a concentration-dependent manner (Fig. 2a). Furthermore, no significant shift in sensitivity is observed when cell lines are co-cultured with MM patient-derived BMSCs (Fig. 2b; Supplementary Fig. 2b). A single treatment with IC_50_ dose of BI8622/BI8626 significantly reduced the proliferation of MM cells lines over 96 h (Fig. 2c; Supplementary Fig. 2c), and analysis of cell cycle distribution demonstrated an accumulation of cells in S (*p* ≤ 0.044) and G2/M (*p* ≤ 0.048) with an associated decrease in G1 (*p* ≤ 0.0024; Fig. 2d). Importantly, inhibition of HUWE1 has no significant effect on the viability (Supplementary Fig. 2c) or colony forming ability (Fig. 2e, f) of normal BM mononuclear cells, suggesting a favorable therapeutic index.

For shRNA studies, JJN3 cells were transduced with doxycycline-inducible lentivirus encoding three distinct HUWE1–targeting shRNA constructs; cells transduced with a non-targeting control (NTC) shRNA were used as a control. Following puromycin selection, cells were seeded at a density of 3 × 10^5^ cells/ml and proliferation evaluated over 96 h. Upon addition of doxycycline, all shRNA sequences reduced expression of HUWE1 (Fig. 2g), leading to decreased proliferation (Fig. 2h). Effects on proliferation were greatest using construct shHUWE1-3 (*p* = 0.008) and this shRNA was used in subsequent experiments. As with HUWE1 inhibitors, reduction in proliferation was maintained when HUWE1 knockdown cells were co-cultured with BMSCs (Fig. 2i). Analysis of cell cycle distribution 72 h post-doxycycline induction demonstrated a significant accumulation of cells in S-phase (*p* = 0.003) with an associated decrease in G1 (*p* = 0.019), and this effect was maintained 1 week post-treatment. This is consistent with finding by Choe et al. who reported an S-phase arrest in HUWE1-depleted cells [26]. While both inhibition and knockdown of HUWE1 effectively block the proliferation of MM cell lines, there is little associated activation of caspase-induced apoptosis (Supplementary Fig. 2d-e) suggesting that the effect of HUWE1 depletion is primarily cytostatic.

### HUWE1 expression correlates with MYC expression in MM

**Fig. 3:**
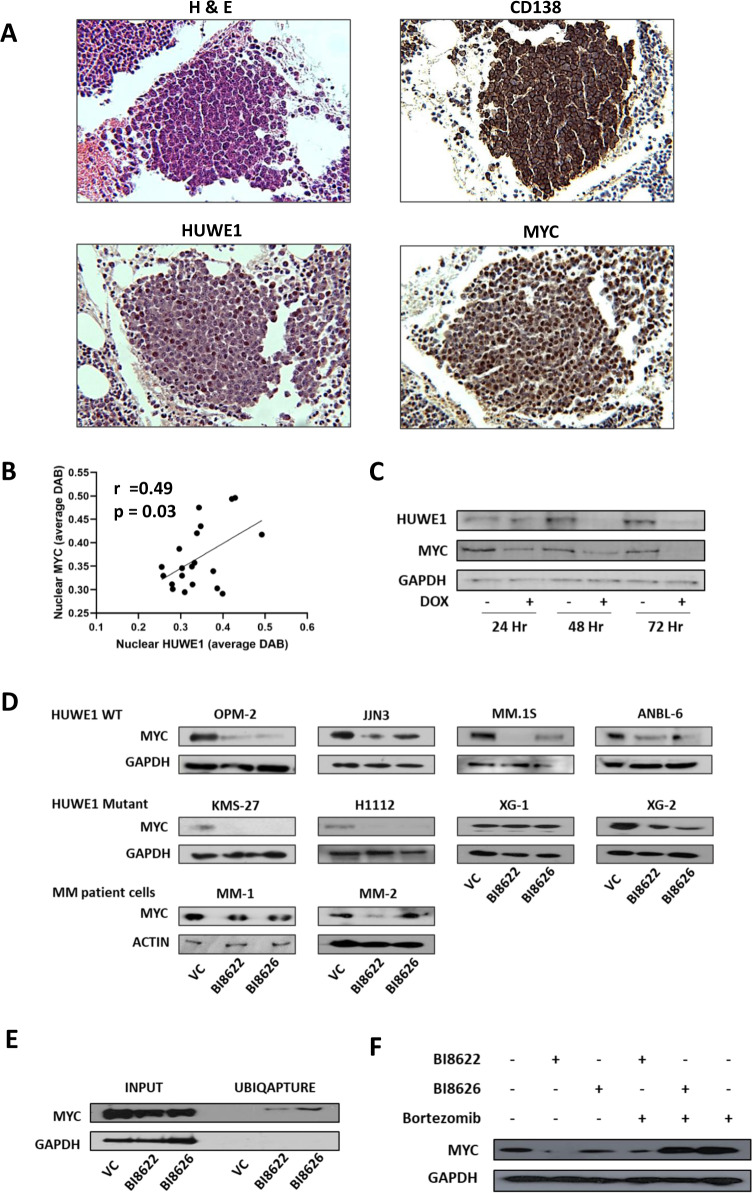
HUWE1 expression correlates with MYC expression in multiple myeloma. **a** Representative IHC from MM patient trephines: hematoxylin and eosin (top left), CD138 (top right), HUWE1 (bottom left), and MYC (bottom right). **b** Correlation of nuclear HUWE1 and nuclear MYC DAB staining in MM patient trephines (*n* = 20); staining intensity was quantified using QuPath. Correlation coefficient and statistical analysis was determined using Pearson’s test. **c** HUWE1 and MYC protein expression in JJN3 cells expressing shHUWE1-3 24, 48 and 72 h post-treatment with 1 μg/ml doxycycline (dox). **d** MYC protein expression in a panel of HUWE1 wild-type (WT) cell lines, HUWE1 mutant cell lines and MM patient samples cultured in the presence of 15 μM BI8622 or BI8626 for 24 h; DMSO treated cells were used as a vehicle control (VC). **e** MYC ubiquitination following HUWE1 inhibition. Left side of blot shows MYC expression in MM.1S cells cultured in the presence of 15 μM BI8622 or BI8626 (Input); right side of blot shows ubiquitinated MYC enriched from input lysate using UBIQAPTURE-Q ubiquitin affinity matrix. **f** MYC expression in MM.1S cells cultured for 6 h with 15 μM BI8622 or BI8626 ±3 h pulse with 20 nM bortezomib.

Qi et al. previously reported that HUWE1 parallels MYC expression in MYC-driven B-cell lymphomas [21]. Here we show that nuclear staining of HUWE1 in MM BM trephines positively correlates with nuclear staining of MYC (*r* = 0.49; *p* = 0.03; Fig. 3a, b). Furthermore, we demonstrate that MYC expression comparably decreases along with HUWE1 expression in knockdown cells (Fig. 3c). Correspondingly, inhibition of HUWE1 with BI8622 and BI8626 leads to decreased MYC expression across a panel of HUWE1 wild-type and mutant cell lines and patient-derived MM cells, with the exception of XG-1 HUWE1 mutant cell line. MYC mRNA levels are not altered in the absence of HUWE1 (Supplementary Fig. 3a-b), indicating that MYC is post-translationally regulated by HUWE1. Ubiquitination assays demonstrate a decrease in K63- and increase in K48-linked polyubiquitination of MYC when HUWE1 is inhibited (Fig. 3e; Supplementary Fig. 3c) suggesting that in the absence of HUWE1, MYC is targeted for proteasomal degradation. This is further confirmed by demonstrating that MYC protein expression is rescued in HUWE1 inhibitor treated cells by addition of the proteasome inhibitor bortezomib (Fig. 3f).

### Identification of HUWE1 substrates in MM

**Fig. 4:**
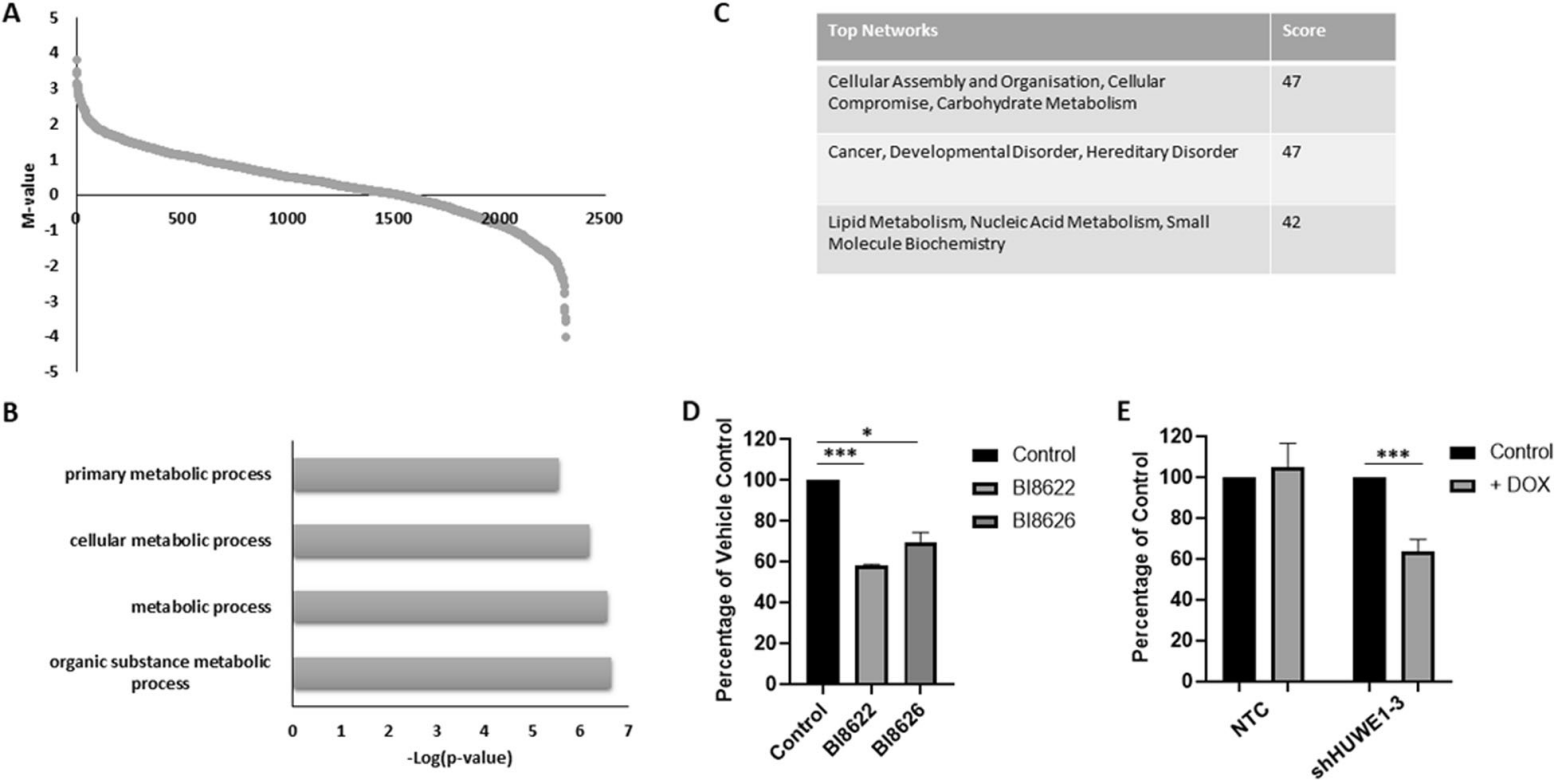
Identification of HUWE1 substrates in multiple myeloma. **a** Protein arrays were incubated with cell lysates from JJN3 cells expressing NTC or shHUWE1, and pan-ubiquitin chains detected using tandem ubiquitin binding entities (TUBE). Graph represents significant changes in protein ubiquitination in the absence of HUWE1 (*n* = 2314), positive values indicate putative substrates; *M*-value measures magnitude of change. **b** Top gene ontology processes enriched in putative HUWE1 substrates (biological processes). **c** Top networks enriched in putative HUWE1 substrates identified through Ingenuity Pathway Analysis. **d** Intracellular glutamine concentration of JJN3 cells cultured with vehicle control or 15 μM BI8622 or BI8626; expressed as a percentage of vehicle control (*n* = 3). **e** Intracellular glutamine concentration of JJN3 cells expressing NTC shRNA or shHUWE1-3 ±1 μg/ml doxycycline (DOX); expressed as a percentage of control (*n* = 3). Statistical significance was determined using paired *t*-tests; *p ≤ 0.05, ***p ≤ 0.001.

To further elucidate the function of HUWE1 in MM, we employed Snapshot proteomics to identify substrates of HUWE1. This technique detects ubiquitination reactions in whole cell lysates using protein microarrays as a substrate platform. Microarrays were incubated with cell lysates from NTC or HUWE1 knockdown cells, and pan-ubiquitin chains detected. Data were obtained from 21,065 proteins, and 2223 proteins demonstrated significant changes (*p* < 0.05) in ubiquitination (Fig. 4a; Supplementary Table 1). Positive changes in *M*-value represent putative HUWE1 substrates, while negative *M*-values represent proteins with increased ubiquitination. In total, 1516 proteins exhibited a statistically significantly decrease in ubiquitination in the absence of HUWE1. While some of the smaller changes are likely to represent noise in the analysis, no cut off in *M*-value was employed as these can also indicate slight but highly reproducible modifications in ubiquitination such as chain shortening or lengthening. Gene ontology and ingenuity pathway analysis of putative substrates of HUWE1 revealed a strong enrichment for metabolic processes (Fig. 4b, c; Supplementary Fig. 4a-c). MM cells display alterations in many metabolic pathways [27], and proteins from pathways including glycolysis, glutaminolysis, and fatty acid metabolism exhibit modified ubiquitination in the absence of HUWE1 (Supplementary Table 2).

As depletion of glutamine has been demonstrated to induce a MM-specific degradation of MYC [28], similar to our findings with HUWE1 inhibition or knockdown, we investigated the effect of loss of glutamine on sensitivity of MM cell lines to HUWE1 inhibitors. Comparable with previous findings, we show decreased MYC expression in cells cultured without L-glutamine (Supplementary Fig. 5a) and demonstrate reduced sensitivity to HUWE1 inhibition in these cells (Supplementary Fig. 5b). No changes to the ubiquitination status of principle glutamine transporters (SLC1A5, SLC38A1, and SLC38A2) were detected in HUWE1-depleted cells, however, altered ubiquitination was identified in a number of transporters, enzymes, and electrochemical gradients related to glutamine metabolism (Supplementary Table 2). We demonstrate that inhibition or knockdown of HUWE1 results in a significant decrease in intracellular glutamine levels in MM cell lines (*p* ≤ 0.012; Fig. 4d, e), suggesting that reduction in glutamine contributes to MYC degradation in the absence of HUWE1; future studies will aim to dissect the mechanism behind this phenomenon.

### Combination of HUWE1 depletion and conventional MM therapies induces synergistic anti-MM activity

**Fig. 5:**
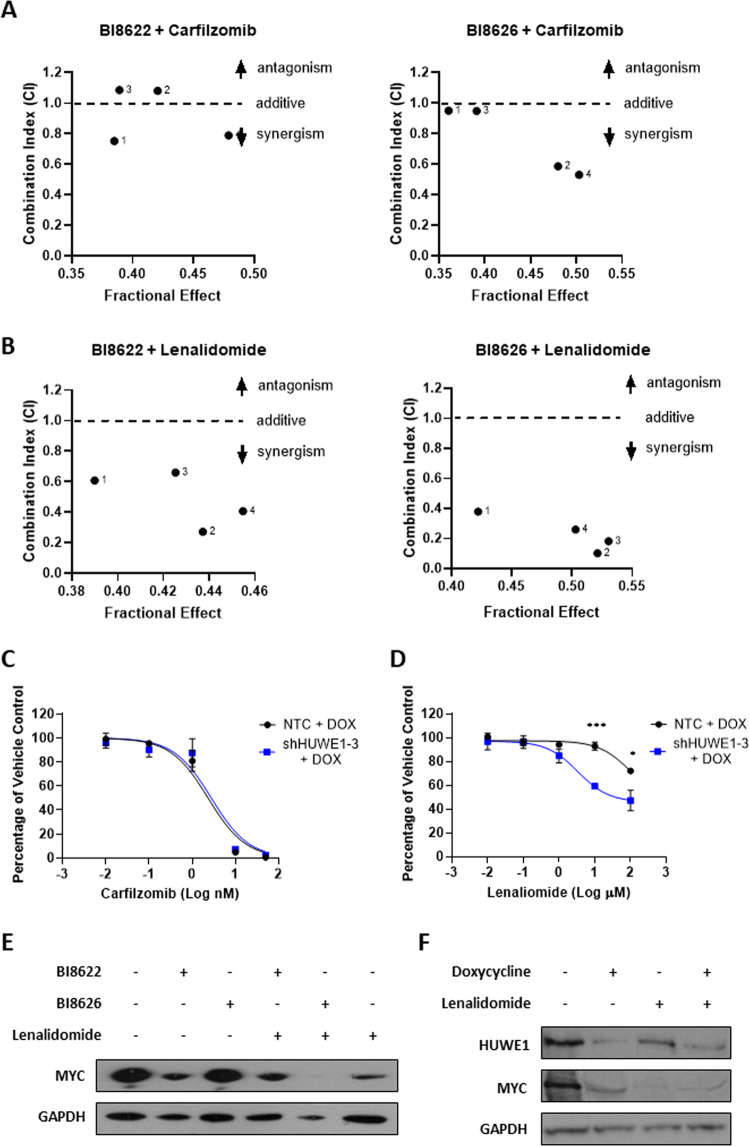
Synergistic activity of HUWE1 inhibition or knockdown with conventional multiple myeloma therapies. **a** MM.1S cells were treated with BI8622 (7.5 or 15 μM) or BI8626 (7.5 or 15 μM) plus carfilzomib (2.5 and 5 nM) for 24 h and analyzed for viability. **b** MM.1S cells were treated with BI8622 (7.5 or 15 μM) or BI8626 (7.5 or 15 μM) plus lenalidomide (1 or 10 μM) for 72 h and analyzed for viability. **a** and **b** display combination index (CI) values whereby a CI < 1 indicates synergy, 1 indicates an additive effect, and >1 indicates antagonism (*n* = 3). **c** Dose response of JJN3 cells expressing doxycycline‐inducible shHUWE1-3 or NTC shRNA to carfilzomib; 72 h post-dox treatment cells were seeded at 3 × 10^5^/ml, treated with carfilzomib, and analyzed for viability after 24 h (*n* = 3). **d** Dose response of JJN3 cells expressing doxycycline‐inducible shHUWE1-3 or NTC shRNA to lenalidomide; 72 h post-dox treatment cells were seeded at 3 × 10^5^/ml, treated with lenalidomide, and analyzed for viability after a further 72 h (*n* = 3). Statistical significance was determined using paired *t*-tests; *p ≤ 0.05, ***p ≤ 0.001. **e** MYC protein expression in MM.1S cells treated with BI8622 or BI8626 alone (15 μM), lenalidomide alone (10 μM), or a combination of both. **f** MYC protein expression in JJN3 cells expressing doxycycline‐inducible shHUWE1-3 treated with doxycycline alone (1 μg/ml), lenalidomide alone (10 μM), or a combination of both.

Proteasome inhibitors and IMiDs are critical components of MM therapy. The combination of HUWE1 inhibitors with carfilzomib enhanced the antiproliferative effects of either agent alone, and combination index (CI) analysis demonstrated some synergistic anti-MM activity (Fig. 5a; Supplementary Fig. 6a). HUWE1 inhibitors in combination with the IMiD lenalidomide similarly enhanced the antiproliferative effects of either agent alone and exhibited strong synergistic activity with both BI8622 and BI8626 at all doses tested (Fig. 5b; Supplementary Fig. 6b). Correspondingly, HUWE1 knockdown JJN3 cells are significantly more sensitive to lenalidomide than their control counterparts, while there is no change in sensitivity to carfilzomib (Fig. 5c, d). The mechanism of strong synergy between HUWE1 depletion and lenalidomide may be, in part, mediated through their combined effects on MYC (Fig. 5e, f).

### HUWE1 knockdown decreases MM tumor burden in vivo

**Fig. 6:**
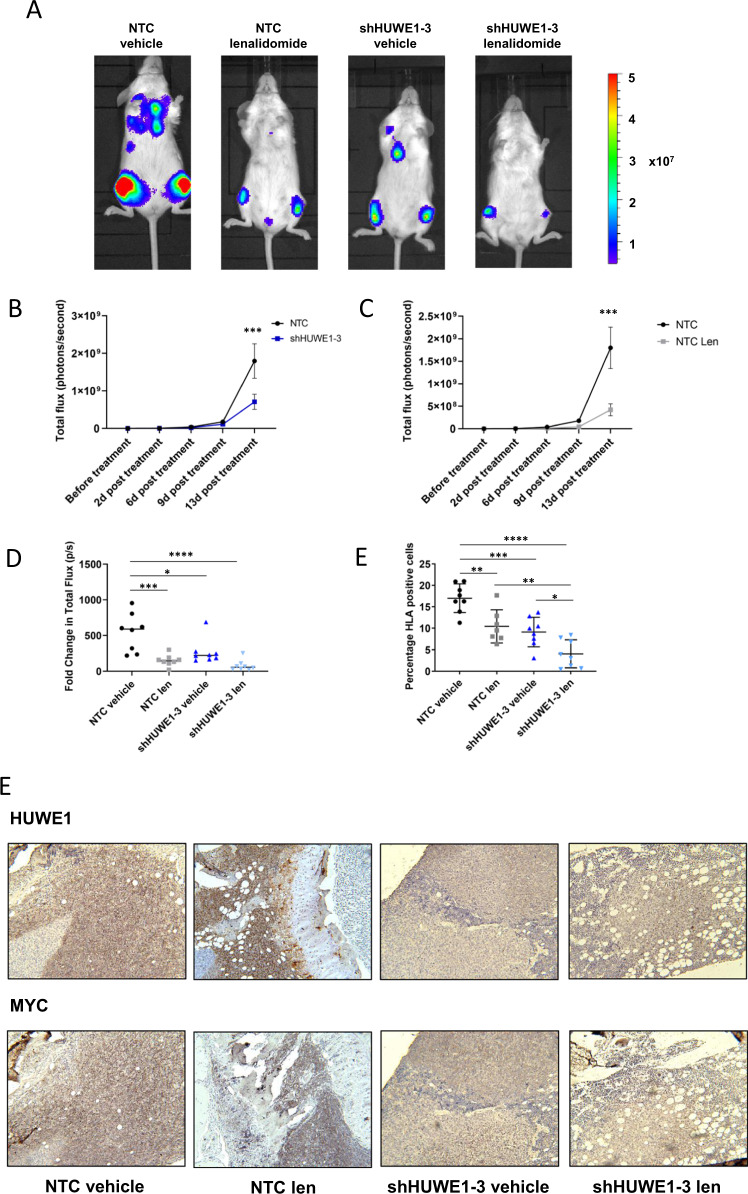
Effect of HUWE1 knockdown on multiple myeloma tumor burden **in vivo**. **a** Representative images of tumor burden as assessed by bioluminescent imaging (BLI): shown are end stage images (13 days post-treatment; 21 days post-injection) for mice injected with JJN3 cells expressing NTC shRNA or shHUWE1-3, treated with doxycycline (1 mg/ml) and vehicle control or doxycycline and lenalidomide (100 mg/kg). **b** Tumor burden was quantified (total flux as measured by BLI) in mice injected with JJN3 cells expressing NTC shRNA or shHUWE1-3 at intervals for 13 days post-treatment with doxycycline. **c** Tumor burden was quantified (total flux as measured by BLI) in mice injected with JJN3 cells expressing NTC shRNA at intervals for 13 days post-treatment with doxycycline plus vehicle or lenalidomide. **d** End stage tumor burden expressed as fold change in total flux as measured by BLI. **e** End stage tumor burden expressed as a percentage of human leukocyte antigen (HLA) positive cells from femora bone marrow flushes. **f** Representative IHC images of sections of distal femur for each treatment group dual stained with CD138/HUWE1 or CD138/MYC (original magnification ×10). *n* = 8 mice/group; data are presented as mean ± SEM; statistical significance was determined using one-way ANOVA; *p ≤ 0.05, **p ≤ 0.01, ***p ≤ 0.001, ****p ≤ 0.0001.

The pharmacokinetic properties for currently available HUWE1 inhibitors BI8622 and BI8626 are unfavorable for in vivo studies [16] and therefore we validated the in vivo effect of HUWE1 depletion using inducible shRNA knockdown JJN3 cells. We have previously demonstrated that JJN3 cells produce an aggressive, short-term model of myeloma in NSG mice, with mice exhibiting signs of morbidity 3 weeks after tumor cell injection [29]. JJN3 NTC or JJN3 shHUWE1-3 cells were injected into NSG mice and following the addition of doxycycline to the drinking water 7 days post-tumor cell injection, mice were treated with either lenalidomide or vehicle and monitored for a further 2 weeks. Addition of doxycycline significantly abrogated tumor growth by 13 days post-treatment as measured by bioluminescence imaging (*p* = 0.03) and percentage of tumor cells (*p* = 0.0006; Fig. 6a, b, d, e); similar effects were seen with single agent lenalidomide (*p* ≤ 0.006; Fig. 6a, c–e). Furthermore, the combination of HUWE1 knockdown and lenalidomide significantly reduced tumor burden compared with either treatment alone (*p* = 0.03; Fig. 6e). Immunohistochemical analysis of CD138/HUWE1 and CD138/MYC validated HUWE1 knockdown in vivo and demonstrated reduction in MYC expression and tumor burden 13 days post-treatment with doxycycline, lenalidomide or a combination of both (Fig. 6f).

## Discussion

HUWE1 is a large HECT-domain E3 ligase that catalyzes ubiquitination of a wide range of protein substrates, implicating it in the regulation of numerous cellular processes including cell proliferation, apoptosis, replication, and DNA damage response [26, 30–32]. It is therefore not surprising that dysregulation of HUWE1 is associated with tumorigenesis. Whether HUWE1 possesses tumor promoting or tumor suppressor activity is a matter of debate and appears to be dependent on cell type. Overexpression of HUWE1 has been identified in many cancers, with one study reporting increased expression of HUWE1 in 7 out of 9 tumor types [33]. Correspondingly, we found increased HUWE1 in malignant compared with healthy plasma cells. HUWE1 can be additionally dysregulated in MM through mutation [15]. We identified MM cell lines expressing HUWE1 mutations distributed in a comparable manner to mutations identified in patients. HUWE1 expression and activity was similar across both HUWE1 mutant and wild-type cell lines. Based on data compiled by the Exome Aggregation Consortium (ExAC) HUWE1 is predicted to be intolerant to loss-of-function mutations (pLI score = 1). Mutations in HUWE1 occur at moderate frequencies in cancers [15] but are commonly associated with X-linked intellectual disability where they can lead to altered or enhanced activity [24, 34]. The association of HUWE1 mutations with *t*(11;14) in patients and cell lines suggests that they may be of functional significance in this subgroup and warrants further investigation. Together with our observations and ExAC predictions it is likely that mutations predominantly lead to altered function in MM rather than total loss of activity. HUWE1 has generated interest as a potential therapeutic target in a number of malignancies [16, 20, 31, 35–37]. In this study we show that small molecule inhibition or knockdown of HUWE1 effectively reduces the proliferation of MM cells without affecting the viability of healthy BM mononuclear cells. Importantly, reduced proliferation was observed even when cells were grown in the presence of BMSCs, which promote the growth and survival of MM cells. Furthermore, knockdown of HUWE1 significantly reduces tumor burden in an aggressive in vivo model of MM validating its potential as a therapeutic target in MM. Combination therapy consisting of PIs or IMiDs form the backbone of MM treatment regimes [38]. Here we demonstrate synergistic activity of HUWE1 knockdown or inhibition with both classes of these drug types. Combinations of HUWE1 inhibition and the IMiD lenalidomide, in particular, lead to significantly reduced tumor growth both in vitro and in vivo. Given the potential of targeting HUWE1 in MM and other malignancies, efforts are currently underway to generate HUWE1 inhibitors with improved physiochemical properties.

MYC, one of the most widely studied substrates of HUWE1, is frequently dysregulated in MM. Regulation of MYC by HUWE1 is context-dependent, with evidence to support both K48- and K63-linked polyubiquitination leading to degradation or activation of MYC, respectively [17, 39]. Previous studies investigating the relationship between HUWE1 and MYC have demonstrated that HUWE1 is required for normal B-cell homeostasis [40, 41], and regulates B-cell maturation through its effects on MYC [41]. Furthermore, high HUWE1 expression has been specifically linked to MYC driven B-cell lymphoma [20]. In this study, we explored the relationship between HUWE1 and MYC in MM, a terminally differentiated B-cell malignancy. We found a positive correlation between MYC and HUWE1 protein expression in MM, and demonstrate that HUWE1 depletion leads to decreased K63-ubiquitination and promotes proteasomal degradation of MYC. This is in keeping with a previous studies reporting K63-ubiquitination of MYC by HUWE1 [18] and demonstrating reduced expression of MYC in murine B cells deficient in HUWE1 [41]. Conversely, HUWE1 inhibition does not affect steady state levels of MYC in a model of colorectal cancer suggesting tissue specific regulation of MYC by HUWE1 [16]. Taken together with earlier studies, our findings indicate that regulation of MYC by HUWE1 is specifically important in the regulation and survival of both normal B cells and malignant B cells.

An ubiquitin-specific proteomics approach was adopted to unravel the mechanisms underlying HUWE1 function in MM. This analysis identified a large number of putative HUWE1 substrates. Consistent with this, structural studies have identified a conformational flexibility in HUWE1 that is predicted to allow ubiquitination of a wide spectrum of substrates [23, 42]. Moreover, HUWE1, in common with other HECT family E3 ligases, can co-operate with different E3s to catalyze ubiquitination, further expanding the number of target substrates [43–45]. Surprisingly, both gene ontology and network analysis of these proteins revealed a strong enrichment for metabolic processes. To date just one study has reported a role for HUWE1 in regulating cancer cell metabolism through K63-linked ubiquitination of the glycolytic enzyme hexokinase 2 [35]. We identified a number of other metabolic enzymes and transporters as potential substrates of HUWE1, suggesting that, in MM, HUWE1 may exhibit a broader effect on tumor metabolism. Altered metabolism contributes to pathogenesis, acquired drug resistance and immune evasion in MM [46–48]. MM cells are more dependent on both glycolysis and glutaminolysis than normal plasma cell counterparts [27]. Interestingly, inhibition of glutaminolysis has been demonstrated to induce MM cell-specific degradation of MYC [28], mirroring the effects of HUWE1 inhibition. The reduction of intracellular glutamine levels in the absence of HUWE1, suggest that glutamine levels may play a role in MYC regulation when HUWE1 is depleted. However, additional studies are required to delineate the role of HUWE1 in regulating MM cell metabolism, particularly in relation to MYC-driven metabolic pathways.

In summary, accumulating evidence points to a context-dependent role for HUWE1 in tumor pathogenesis. Our study demonstrates that in MM, as with other B-cell types, HUWE1 plays a vital role in cell proliferation and regulation of MYC expression. Furthermore, downregulation of HUWE1 enhances the antitumor activity of standard of care therapies, particularly lenalidomide, highlighting the promise of therapeutically targeting HUWE1 in MM.

## Materials and methods

### Cell lines

HS-5, 293T, MM.1S, and ANBL-6 cells were purchased from ATCC and OPM-2, and U266 and JJN3 cells were purchased from DSMZ. XG-1 and XG-2 cells were a kind gift from Dr Catherine Pellat-Deceunynck, INSERM, France. H1112 and KMS-27 cells were a kind gift from Prof Leif Bergsagel, Mayo Clinic, Arizona. All cell lines were maintained according to providers instructions. ANBL6.WT (wild-type), ANBL6.BR (bortezomib resistant), XG-1, and XG-2 were supplemented with IL-6 (2 ng/ml). ANBL6.BR cells were cultured in the presence of 5 nM bortezomib. Cells were routinely tested for mycoplasma, and authentication using short tandem repeat profiling (STR) was conducted by Eurofins Cell Line Authentication Service (Ebersberg, Germany).

### Primary samples

Bone marrow aspirates and trephines from MM patients were obtained with ethical approval from the Northern Ireland Biobank (approval # NIB12-0061) and those involved gave their informed consent in accordance with the Declaration of Helsinki. Human BM mononuclear cells and slides were purchased from Stem Cell Technologies (Cambridge, UK) for use as a normal control. CD138 expression is a hallmark of plasma cells and MM cells. CD138 + cells were enriched from primary samples using the AutoMACS system (Miltenyi Biotech, Germany) according to the manufacturer’s instructions.

### Bone marrow stromal cell (BMSC) culture and MM co-culture assay

MM BMSCs were isolated from BM aspirates as previously described [22]. MM cell lines or MM patient-BMSCs/HS-5 stromal cell line were cultured either alone or together at 1:5 (BMSC/MM) ratio for 48 h, and cell proliferation was measured using the CyQUANT cell proliferation assay, as per manufacturers’ instructions (Thermo Fisher).

### Colony forming unit (CFU) assays

Mononuclear cells from normal donors were cultured in Methocult H4034 Optimum media (StemCell Technologies) in the presence of 15 μM BI8622 or BI8626. After 2 weeks, colonies were counted using a light microscope.

### Immunohistochemistry

Bone marrow trephines were obtained from 25 MM patients, 3 monoclonal gammopathy of undetermined significance (MGUS) patients, and 3 healthy subjects. Consecutive serial sections cut from each FFPET block were cut onto polylysine slides, dewaxed in xylene, and rehydrated through graded alcohol. Heat-induced epitope retrieval was performed in 10 mM citrate buffer pH 6.0 using a microwave oven and endogenous peroxidase activity was inactivated with 0.3% H2O2 in methanol. The following antibodies were used for IHC – CD138 (MCA2459T, Bio-Rad, Hertfordshire, UK), HUWE1 (HPA002548, Merck, Feltham, UK), MYC (ab32072, Abcam, Cambridge, UK), and universal Anti-Mouse/Anti-Rabbit (Vector Laboratories, Peterborough, UK). Immunoreactivity was visualized with ImmPACT DAB peroxidase substrate (Vector Laboratories) and nuclei were counterstained with haematoxylin. Images were taken using blinded analysis with an Olympus BH-2 microscope fitted with an Infinity 3 camera (Lumerera) and analyzed for DAB positivity using QuPATH [49].

### Lentiviral transfection

Transient production of viral vectors encoding SMARTvector Inducible Human HUWE1 shRNA (Dharmacon, Chicago IL, USA) was carried out using 293T cells. 24 h after transfection the media was refreshed and the culture supernatant was harvested 24 h following medium exchange, filtrated (0.45 μm), supplemented with polybrene, and used to transduce MM cell lines or stored at −80 °C for later use. FACS analysis was performed following transduction to select for cells expressing the TurboRFP reporter gene after treatment with 1 µg/ml doxycycline (Merck). shRNA sequences are shown below. Cells infected with vector expressing NTC shRNA were used as controls.

H1 - ATCGAAAGAACGCAATGCT

H2 - TTCGGCACAAATCTACTTC

H3 – TGGACGAATGCCTAGCTGG

### Cell assays

Cell viability and apoptosis was assessed using CellTiter-Glo^®^ and Caspase-Glo assays, respectively (Promega, Southampton, UK). Proliferation was assessed using CyQUANT (Thermo Fisher) according to the manufacturer’s instructions. Intracellular glutamine concentration was measured using the Glutamine Assay Kit (Abcam) according to the manufacturer’s instructions.

### Cell cycle analysis

Cells were harvested, washed in phosphate-buffered saline and cell cycle analyzed using either propidium iodide staining or EDU incorporation. For propidium iodide staining cells were fixed in 70% ethanol and subsequently stained with 50 μg/ml propidium iodide solution containing 0.25 mg/ml RNase. For EDU incorporation cells were fixed in 2% formaldehyde and permeabilized by subsequent addition of 70% ethanol. Cells were then washed in PBS containing 0.05% Triton X-100 before detection of EdU-substituted DNA (EdU-DNA). Detection of EdU-DNA was performed according to the Click-iT EdU Alexa Fluor 488 Imaging Kit as per manufacturer’s instructions (Thermo Fisher). Bulk DNA was subsequently stained with DAPI. DNA content was measured with an LSRII flow cytometer and subpopulations were identified using FACS Diva and Flowing Software (Turku Centre for Biotechnology, Finland).

### Western blotting

Immunoblotting was carried out as previously described using antibodies against HUWE1, (ab70161, Abcam), c-MYC (ab32072, Abcam), GAPDH (ab181602, Abcam) and ubiquitin (sc-8017, Santa Cruz, Heidelberg, Germany) and secondary antibodies anti-mouse and anti-rabbit (DAKO, Cambridgeshire, UK). Ubiquitinated proteins were isolated using UbiQapture-Q (Enzo Life Sciences, Exeter, UK) to bind both mono- and poly-ubiquitinated proteins independent of lysine chain linkage, or tandem ubiquitin binding entity (TUBE) antibodies specific for either K48 or K63 linkages (UM607 and UM604, Life Sensors, Malvern, USA), according to the manufacturer’s instructions. A proportion (25 μg) of total cell lysate was retained for use as an input control and 250 μg of remaining lysate was bound to the appropriate affinity matrix to capture ubiquitinated proteins. Input controls and protein eluted from the matrix were subsequently analyzed by immunoblotting. Blots were visualized using WesternBright^™^ ECL horseradish peroxidase substrate (Advansta, UK), scanned into the G:BOX gel doc system (Syngene, Cambridge, UK) and analyzed using GeneTools.

### Snapshot proteomics

JJN3 cells were transduced with an NTC shRNA or shRNA targeting HUWE1 and selected in 0.5 µg/ml puromycin 48 h post-transduction. Following selection, cells were harvested and lysed in a mild cell lysis buffer (50 mM Tris–HCl pH 7.5, 150 mM NaCl, 1% NP-40, 1% sodium deoxycholate, 10% glycerol, 1 mM PMSF). Fresh lysates were shipped to AVMBiomed (Pottstown, PA) for immediate use in snapshot proteomics (U.M.A.P) to identify ubiquitinated substrates across ~20,000 proteins. Briefly, HuProt protein microarrays were incubated in parallel with lysates from NTC cells and HUWE1 knockdown cells for 90 min at room temperature and biotin-tagged TUBEs (Biotin TUBES2, Boston Biochem) were then employed for detection of pan-ubiquitin chains across both arrays followed by visualization using Alexa647-conjugated streptavidin. Microarrays were scanned with a GenePix 4100A scanner (Molecular Devices) and images were gridded and quantitated using GenePix Pro (v7) software. Median intensities (features and local backgrounds) were utilized, and signal to noise ratio calculated. Values were then normalized to biological controls within each array. Duplicate features (representing identical protein) were summarized by average and standard deviation. These values were compared between arrays (WT HUWE1 minus NTC) then Loess transformed by print tip and location to remove technical sources of error, resulting in the final estimate of magnitude of change (*M*-value). *T*-test (paired, 2 tailed) was used to assess the statistical significance of each estimate (under the null hypothesis that M = 0).

### In vivo studies

All animal experiments were approved by the University of Sheffield Animal Ethics Committee and the UK Home Office (PPL 70/8799) in strict compliance with the Animal (Scientific Procedures) Act 1986. All mice were group housed in individual ventilated cages with a 12 h light/dark cycle and ad libitum access to food and water. Mouse numbers (*n* = 8/group) were calculated prospectively using G*Power software. End stage and in vivo analyses were blinded.

Female, 7-9-week-old NOD *scid* gamma (NSG, NOD.Cg-*Prkdc*^*scid*^ *Il2rg*^*tm1Wjl*^*/SzJ*) mice (Charles River Laboratories) were randomized into groups and injected i.v. with 10^6^ human JJN3 cells expressing NTC shRNA (*n* = 16) or shHUWE1-3 (*n* = 16) via the tail vein. After 1 week all mice were given doxycyline (1 mg/ml in 1% sucrose) in drinking water and then treated with either lenalidomide (100 mg/kg, 5×/week) or vehicle (1% methyl cellulose). Tumor burden was monitored weekly by bioluminescent imaging using an IVIS Lumina II (Caliper Life Sciences). All mice were sacrificed after 2 weeks of treatment. BM was flushed from left femora and human leukocyte antigen (HLA) positive cells were assessed by flow cytometry to measure tumor burden using a FACSCalibur (BD Biosciences). Right femora were fixed in 10% formalin, decalcified, embedded in paraffin and 3 µm longitudinal sections were dual stained with CD138/HUWE1 or CD138/MYC as described above.

### Statistical analysis

Statistical analyses were performed in GraphPad Prisim 8. Differences between groups were evaluated using one-way ANOVA or Student’s *t* test; correlation coefficients were evaluated using Pearson’s test; log-rank test was used for survival analysis. Data are presented as mean ± S.D of at least three biological replicates as indicated in figure legends.

## Supplementary information

Supplemental Data
