# Supplementary material for: The E3 ligase HUWE1 inhibition as a therapeutic strategy to target MYC in multiple myeloma
Source: Oncogene. 2020 Jun 10;39(27):5001–14. doi: 10.1038/s41388-020-1345-x (PMC7329634; doi:10.1038/s41388-020-1345-x)
Supplement: Supplementary file 1 — Supplemental Data [file 41388_2020_1345_MOESM1_ESM.pdf]

Supplementary Figure Legends

Supplementary Figure 1. HUWE1 is dysregulated in Multiple Myeloma. (**a**) HUWE1 gene expression in normal bone marrow (NBM; n=4), OPM-2 (n=4) and U266 (n=3) MM cell lines; GSE78884. (**b**) qPCR expression of HUWE1 in CD138+ cells from healthy donors (n=3), MM patients (n=5) and 4 MM cell lines (U266, OPM-2, KMS-18, JJN3; n=3). (**c**) Confirmed HUWE1 mutation status in MM cell lines. (**d**) Location of mutations in HUWE1 mutant cell lines.

Supplementary Figure 2. HUWE1 inhibition halts the proliferation of Multiple Myeloma cell lines. (**a**) Top panel shows ubiquitinated HUWE1 in JJN3 cells treated with vehicle control (VC), 15 µM BI8622 or 15 µM BI8626 (enriched from cell lysates using UBIQAPTURE ubiquitin affinity matrix). Bottom panel shows HUWE1 expression in total cell lysates used for UBIQAPTURE (input). (**b**) Dose response of JJN3 cells to BI8626 when cultured alone or in co-culture with bone marrow stromal cells (BMSCs; n=3). (**c**) Growth curve of OPM-2, KMS-18, MM.1S and normal bone marrow (NBM) cells cultured in the presence of 15 µM BI8622 or BI8626; DMSO treated cells were used as a vehicle control (n=3). (**d**) Caspase 3/7 activity in JJN3 cells treated with increasing doses of BI8622 or BI8626 (n=3). (**e**) Cell cycle analysis of propidium iodide stained JJN3 cells 24 hrs post-treatment with 15 µM BI8622 or BI8626.

Supplementary Figure 3. Depletion of HUWE1 induces proteasomal degradation of MYC. (**a**) Fold change in mRNA expression of MYC in MM.1S cells cultured in the presence of 15 µM BI8622 or BI8626 for 24 hrs relative to mRNA expression in vehicle treated control cells (n=3). (**b**) Fold change in mRNA expression of MYC in JJN3 cells expressing shHUWE1-3 relative to cells expressing NTC shRNA, 72 hrs post-dox treatment (n=3). (**c**) Top panel shows K48 and K63 ubiquitinated MYC in JJN3 cells treated with vehicle control (VC), 15 µM BI8622 or 15 µM BI8626 (enriched from cell lysates using K48 or K63 specific

tandem ubiquitin binding entities (TUBE) affinity matrix). Bottom panel shows MYC expression in total cell lysate used for input.

Supplementary Figure 4. Ingenuity Pathway Analysis (IPA) for putative HUWE1 substrates. (**a**) Network 1 – Cellular assembly and organization, cellular compromise, carbohydrate metabolism. (**b**) Network 2 – Cancer, development disorder, hereditary disorder. (**c**) Network 3 – Lipid metabolism, nucleic acid metabolism, small molecule biochemistry.

Supplementary Figure 5. Loss of glutamine affects MYC expression and HUWE1 inhibitor sensitivity. (**a**) MYC protein expression in JJN3 and MM.1S cells cultured for 24 hrs in the presence or absence of L-glutamine. (**b**) 24 hr dose response of JJN3 (left) and MM.1S (right) cells to BI8626 when cultured in the presence or absence of L-glutamine.

Supplementary Figure 6. Synergistic activity of HUWE inhibition or knockdown with conventional Multiple Myeloma therapies. (**a**) JJN3 cells were treated with BI8622 or BI8626 (7.5 or 15 µM) plus carfilzomib (2.5 and 5 nM) for 24 hrs and analyzed for viability. (**b**) JJN3 cells were treated with BI8622 or BI8626 (7.5 or 15 µM) plus lenalidomide (1 or 10 µM) for 72 hrs and analysed for viability. **a** and **b** display combination index (CI) values whereby a CI <1 indicates synergy, 1 indicates an additive effect and >1 indicates antagonism (n = 3).

Supplementary Figure 1

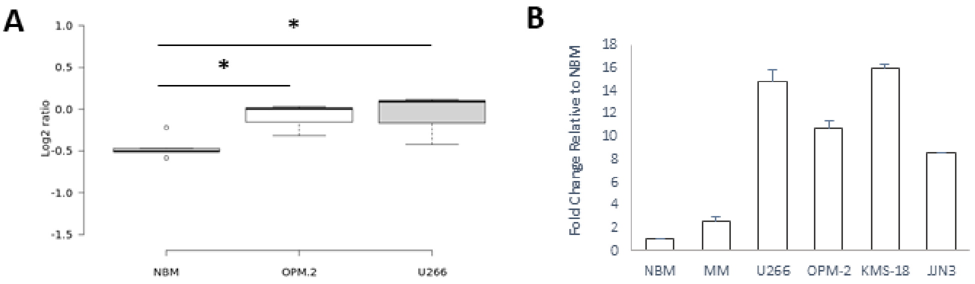

C

Cell Line	Mutation Type	Position	Reference	Alternative	Change
U266 t(11;14)	Missense	53620479	C	T	G1196R
H1112 t(11;14)	Splice	53641493	AC	A	Splice
KMS-27 t(11;14)	Missense	53612028	C	T	V1649M
XG-1 t(11;14)	Missense	53588777	C	T	E2483K
XG-2 t(12;14)	Missense	53641575	A	T	S727R

D

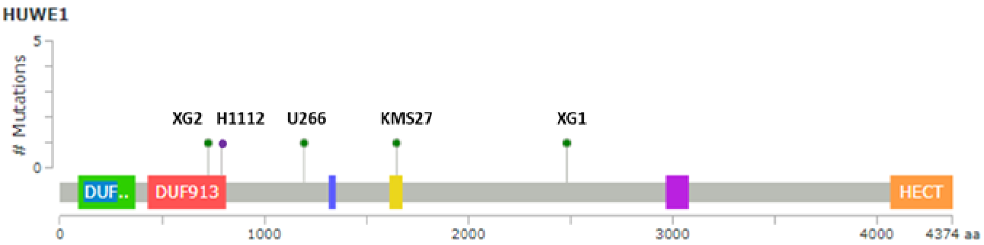

Supplementary Figure 2

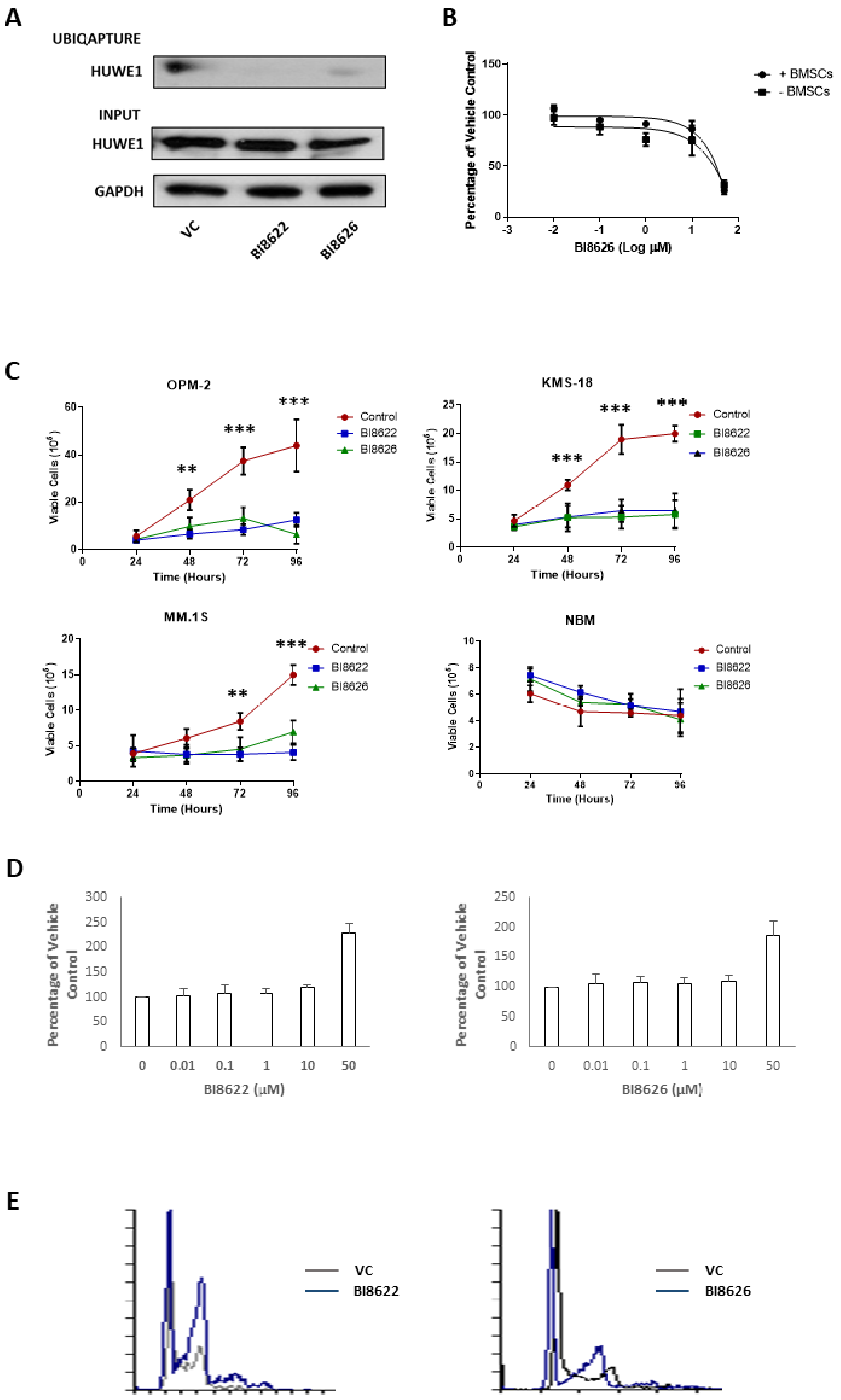

Supplementary Figure 3

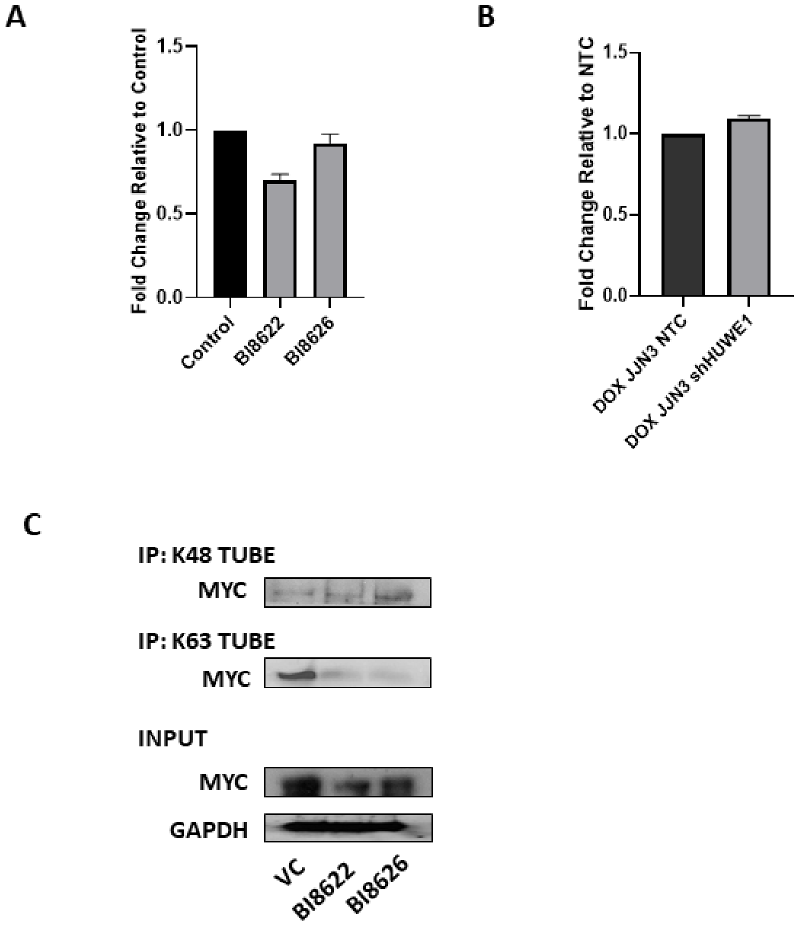

Supplementary Figure 4

A

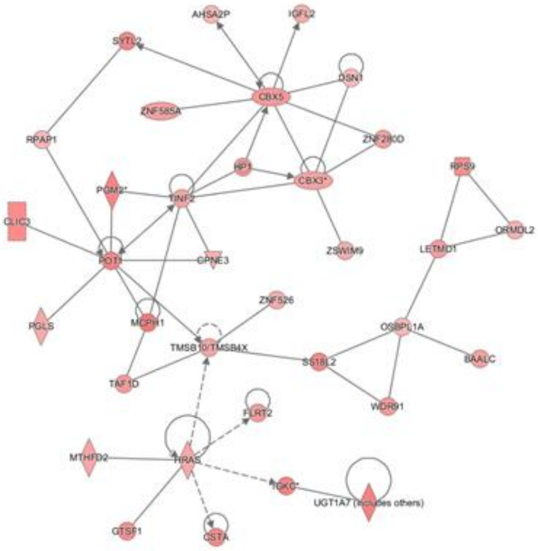

B

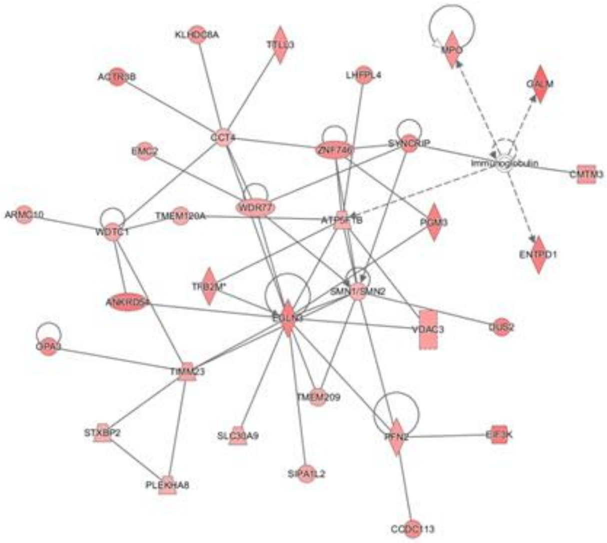

C

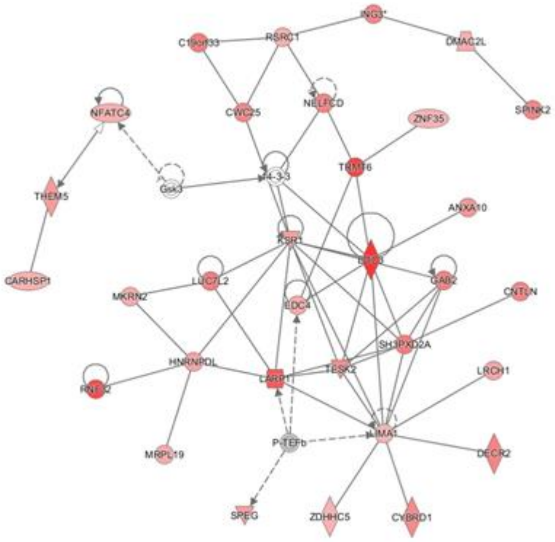

Supplementary Figure 5

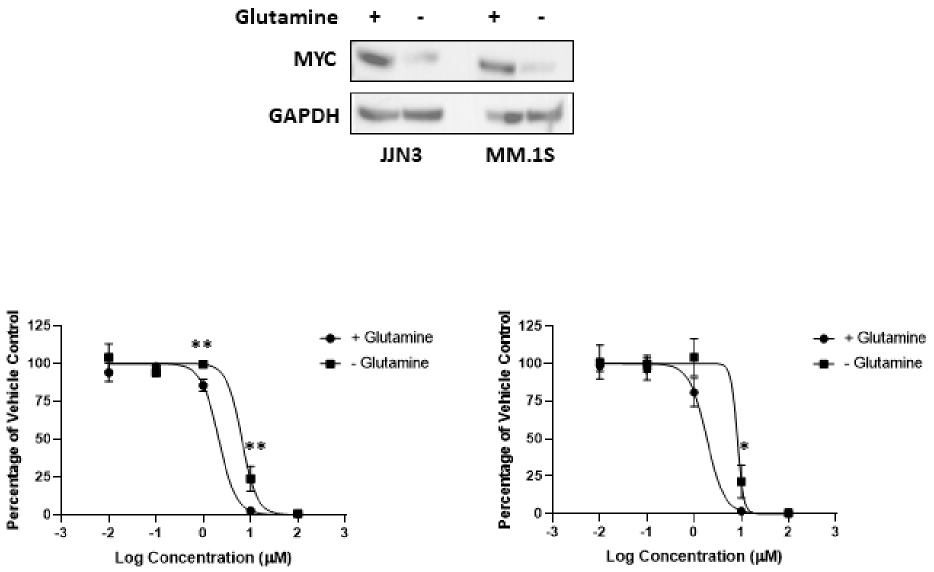

Supplementary Figure 6

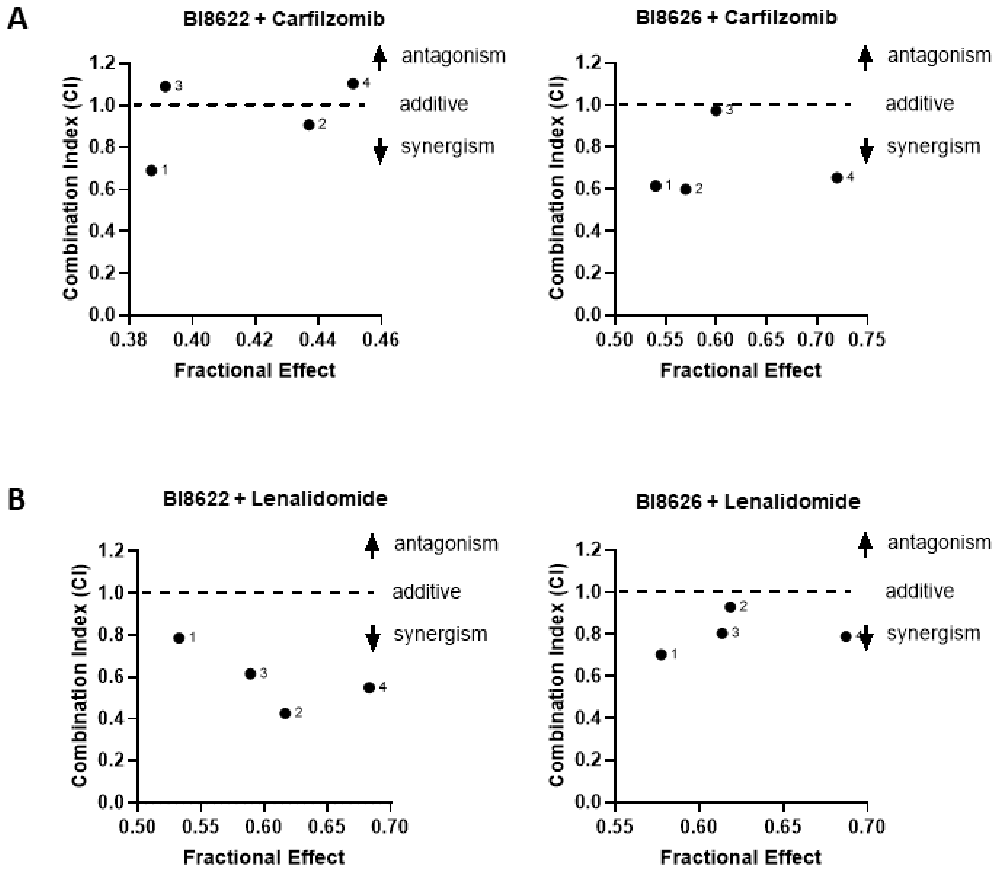

Supplementary Table 1. Proteins with a significant change in ubiquitination in NTC vs HUWE1 knockdown JJN3 cells. Mvalue represents magnitude of change.

Name	ID	Mvalue	stdev	pval
ABCB5	ABCB5	1.2379856	0.001417	0.011626
ABCB7	NM_004299.3	0.8846888	0.07779	0.027708
ABCC6	NM_001079528.1	-0.1622087	0.004164	0.032419
ABCD1	NM_000033.2	-1.5877822	0.015192	0.041915
ABCF3	BC009253.2	0.3565866	0.00684	0.007565
ABCG8	ABCG8	1.5449861	0.00628	0.002897
ABHD1	BC039576.1	0.322729	0.003318	0.002019
ABI1	JHU00289	0.1333797	0.01156	0.049881
ABL2	BC065912.1	0.8634941	0.011054	0.039696
ABR	NM_001092.3	1.2254333	0.006895	0.048498
AC011504.3_frag	JHU10748	-0.6900143	0.002671	0.021461
AC236656.3_frag	JHU03294	2.9655551	0.00709	0.008683
AC254631.1_frag	JHU15176	1.2499939	0.041447	0.020291
ACACA	NM_000664.3	1.2083305	0.015784	0.025195
ACADS	BC025963.1	0.9377184	0.108941	0.041155
ACAP1	NM_014716.2	-0.0353537	0.004677	0.027983
ACBD4	BC041143.1	0.8999142	0.009541	0.023377
ACOT6	ACOT6	1.7549521	0.010656	0.01267
ACOT9	BC012573.1	-1.5208905	0.016637	0.004798
ACSL5	NM_203379.1	-0.1075296	0.009128	0.029127
ACSS2	BC098422.1	1.552379	0.01169	0.031907
ACSS2	JHU15004	-1.774909	0.068501	0.022936
ACTA2	NM_001613.1	1.3273483	0.007422	0.005188
ACTN1	NM_001102.2	-0.0102284	0.028903	0.017778
ACTR3B	NM_020445.4	1.9701049	0.014573	0.005018
ACYP1	NM_001107.3	-0.4632207	0.026554	0.026667

ADAM15	NM_207191.1	0.6455193	0.001731	0.021613
ADAM23	NM_003812.2	0.131236	0.012498	0.009011
ADAM5	BC067864.1	-0.5597483	0.035246	0.014343
ADAMDEC1	NM_014479.3	0.9656085	0.005478	0.014229
ADAP2	BC033758.1	1.5479695	0.003083	0.014064
ADAP2	NM_018404.2	0.3385344	0.013423	0.001946
ADCK5	BC031570.1	-0.6312002	0.044516	0.007964
ADCY9	NM_001116.2	0.5551654	0.002218	0.023625
ADGRB1	JHU17697	-1.7118292	0.038686	0.049338
ADGRF3	JHU17532	0.8480442	0.006431	0.044017
ADIPOR1	NM_015999.2	0.6053884	0.005708	0.011107
ADM2	NM_024866.5	0.1071923	0.020346	0.033563
ADORA2B	BC025722	-1.072024	0.021496	0.010678
ADORA3	NM_000677.2	0.7329317	0.002399	0.02218
ADORA3	BC041707.1	0.5507004	0.007134	0.045387
ADPRH	NM_001125.2	-0.5941672	0.002007	0.002865
ADPRM	BC070155.1	-0.6247814	0.039961	0.035408
ADRBK2	BC036797.1	-0.1892493	0.034731	0.022259
ADSSL1	NM_152328.3	0.824319	0.003139	0.020977
AGPAT2	BC019292.1	-0.2431009	0.009036	0.046007
AGPAT5	NM_018361.3	-0.1822879	0.015956	0.00682
AGTR1	JHU16058	0.0062105	0.002213	0.01484
AGTR1	JHU16058	-0.0167878	0.041359	0.018683
AGTR1	JHU16058	-0.0427071	0.016727	0.000506
AGTR1	JHU16058	-0.3341375	0.004862	0.037667
AGTR2	NM_000686.3	0.2302418	0.05783	0.048672
AHSA2	NM_152392.1	1.0919684	0.005992	0.000887
AICDA	BC006296.2	2.1314118	0.033559	0.017143
AIF1L	BC021253.2	1.4570834	0.001725	0.010181
AJ315540.1_frag	JHU12499	-0.2014377	0.013333	0.005664

AJUBA	NM_032876.4	-0.1974958	0.02711	0.034099
AK1	BC001116.2	1.2125324	0.067983	0.015707
AK127903.1_frag	JHU14464	0.8600003	0.012458	0.035092
AK2	NM_013411.3	-0.3560717	0.018877	0.044197
AK290580.1_frag	JHU12020	0.8372345	0.050606	0.036181
AKAP5	NM_004857.2	0.0484292	0.005892	0.029842
AKR1B1	NM_001628.2	2.5366252	0.005654	0.000569
AKT3	NM_181690.1	0.4021771	0.013615	0.033847
AKTIP	BC095401.1	0.980142	0.006181	0.042737
AKTIP	NM_022476.2	0.1018883	0.084029	0.008902
AL132660.14_frag	JHU10654	-1.0033659	0.003157	0.016537
AL137002.19_frag	JHU02751	0.6415216	0.022054	0.04087
AL137795.10_frag	JHU10840	1.38996	0.006063	0.005354
AL139378.15_frag	JHU12135	1.7275446	0.011987	0.036247
ALDH2	NM_000690.2	-0.5060772	0.006422	0.022519
ALDH3A1	BC008892.2	0.4930297	0.01121	0.024188
ALDH6A1	NM_005589.2	0.5759199	0.047456	0.025506
ALLC	BC029652.1	1.6754415	0.016348	0.016695
ALOX12P2	BC041851.1	1.1021906	0.015008	0.032982
ALOX5	NM_000698.2	0.2856772	0.014123	0.015946
ALS2	BC071576.1	1.144566	0.002783	0.01405
AMBN	NM_016519.4	0.4088223	0.025044	0.028157
AMBRA1	JHU07433	0.8281729	0.012436	0.036313
AMD1	NM_001634.4	-0.9339715	0.005012	0.01173
AMELX	NM_001142.2	1.2216499	0.003491	0.020394
AMHR2	AMHR2	1.1440386	0.012611	0.02785
AMHR2	AMHR2	-0.0080107	0.036643	0.040657
AMICA1	BC100797.1	1.6878337	0.003492	0.021781
AMN1	NM_207337.1	1.4492755	0.012224	0.048971
AMPD3	AMPD3	0.5023604	0.026817	0.022143

AMY2A	JHU07398	1.1489936	0.019174	0.047138
ANGEL1	BC028714.2	1.2427953	0.024977	0.042666
ANGEL2	BC016966.1	0.3771759	0.000292	0.001973
ANGPT4	NM_015985.2	0.029291	0.008207	0.030453
ANGPTL4	NM_139314.1	2.690199	0.010257	0.023805
ANGPTL5	NM_178127.2	-0.9283079	0.029566	0.042426
ANKK1	NM_178510	1.6245789	0.013842	0.026331
ANKMY1	BC073146.1	0.4096452	0.007285	0.001082
ANKMY1	BC033495.1	0.2437865	0.006868	0.027824
ANKRD10	BC001727.1	0.5840503	0.00652	0.042855
ANKRD22	NM_144590.1	2.2053435	0.00984	0.046467
ANKRD49	NM_017704.2	0.8725505	0.009319	0.025442
ANKRD54	BC066909.1	0.9749565	0.00313	0.009244
ANKS6	BC012981.2	0.5315947	0.010268	0.012817
ANTXR2	NM_058172.3	0.8652819	0.013457	0.044978
ANXA10	NM_007193.3	1.559649	0.001324	0.02563
ANXA11	NM_001157.2	0.0821081	0.038257	0.040249
ANXA2R	BC067873.1	0.3461857	0.027606	0.025284
ANXA2R	NM_001014279.1	0.3202254	0.012749	0.001841
ANXA8L1	BC008813.2	-0.8477325	0.003245	0.005892
AP1S1	NM_057089.1	1.6632685	0.008654	0.031945
AP5Z1	BC037399.1	-0.6748648	0.002613	0.043484
APBB1	NM_145689.1	0.1765898	0.017491	0.020098
APH1A	NM_016022.2	-1.2497619	0.053699	0.033071
APH1A	NM_001077628.1	-1.7791976	0.018888	0.001501
APIP	BC017594.2	0.9230921	0.004091	0.0047
APIP	BC008440.1	0.5184507	0.008418	0.030103
APLP1	BC012889	0.0659934	0.010562	0.038657
APOA1BP	BC100932.1	-0.4185371	0.00894	0.034463
APOA4	BC074764.2	1.4191366	0.005957	0.017399

APOA5	BC101789.1	0.5100324	0.001237	0.00312
APOBEC3C	NM_014508.2	0.411456	0.000906	0.01032
APOBEC3C	BC011739.2	-1.6155491	0.01162	0.023947
APOC1	NM_001645.3	-1.553113	0.004277	0.040841
APOC4	NM_001646.1	1.0418522	0.005303	0.015996
APPBP2	NM_006380.2	-1.5584016	0.019909	0.020586
AQP9	NM_020980.2	1.4783024	0.010948	0.034098
ARAF	NM_001654.1	-1.8679908	0.025246	0.039645
ARAP1	BC056401.1	-0.4651653	0.023588	0.037169
ARG2	BC029050.1	-0.5545862	0.010203	0.01302
ARHGAP1	BC018118	0.2214558	0.001887	0.021873
ARHGAP12	BC094719.1	-0.1600941	0.006606	0.007943
ARHGAP44	ARHGAP44	0.0444959	0.013678	0.032595
ARHGAP5-AS1	BC007251.1	0.0803595	0.018263	0.010118
ARHGAP8	JHU17082	0.5787821	0.012897	0.044881
ARID5A	BC067301.1	-0.8524928	0.010693	0.033312
ARL2	NM_001667.1	-3.1732288	0.012499	0.038164
ARL5B	NM_178815.3	-0.1168382	0.003567	0.001403
ARL6IP6	NM_152522.2	0.0530365	0.003728	0.043209
ARL8A	NM_138795.2	-0.3909647	0.049187	0.023458
ARL9	NM_206919.1	0.8338805	0.006743	0.02192
ARMC10	JHU08648	0.411522	0.006607	0.049524
ARMCX3	NM_016607.3	1.61537	0.010465	0.031053
ARRB1	NM_004041.3	-0.0228245	0.021422	0.019764
ARRDC2	BC022516.1	0.6898897	0.001162	0.007911
ARRDC4	BC070100.1	1.484354	0.010872	0.00615
ARSK	NM_198150.2	-1.4908121	0.011463	0.023417
ASAP3	NM_017707.2	0.2108721	0.004856	0.014004
ASB10	ASB10	-1.248257	0.009357	0.001007
ASB17	NM_080868.1	0.9800743	0.029282	0.035544

ASB4	NM_145872.1	1.6518664	0.015456	0.03042
ASB7	NM_198243.1	-0.2628815	0.039896	0.024875
ASCC3	NM_022091.3	-1.0858774	0.025187	0.043032
ASCL1	NM_004316.2	0.9275101	0.00111	0.008499
ASF1A	NM_014034.1	-1.7237052	0.004401	0.005255
ASIC2	NM_001094.4	0.1681937	0.019433	0.03891
ASMTL	BC002508.2	-0.0381315	0.013622	0.025861
ASNSD1	NM_019048.1	1.9640351	0.003622	0.018209
ASPSCR1	BC018722.1	0.0561096	0.009464	0.007392
ASXL1	BC064984.1	-0.1564962	0.014607	0.03652
ATF2	NM_001880.2	0.8216138	0.009035	0.03783
ATG13	BC006191.2	1.0470215	0.012912	0.021886
ATG7	NM_006395.1	0.3516402	0.005892	0.023602
ATN1	BC051795.2	0.855402	0.043638	0.028815
ATP1A1	BC003077	1.1421088	0.018836	0.047014
ATP1B3	NM_001679.2	-1.1970382	0.017845	0.019003
ATP2A3	BC035729	0.6863891	0.033205	0.006742
ATP5B	NM_001686.3	1.1588066	0.06706	0.027196
ATP5S	BC011549.1	0.2244664	0.016883	0.021972
ATP6V0A2	BC022300.1	-1.3505203	0.009916	0.043416
ATP6V1G3	BC101129.2	-0.4158015	0.006635	0.016141
ATPAF1	BC018781	0.6340262	0.002344	0.016894
ATPIF1	BC004955.1	0.7773614	0.004611	0.027745
ATXN7L1	NM_152749.2	0.2478472	0.011878	0.041692
ATXN7L1	BC003517.1	0.0657683	0.001997	0.020149
AVPR1B	NM_000707	1.4961517	0.023812	0.013362
AXL	BC032229.1	0.0773847	0.002833	0.022265
AZI2	BC001853.2	-0.3162673	0.030321	0.022154
B3GALNT2	NM_152490.1	0.0570246	0.01965	0.003694
B3GALT1	NM_020981.2	0.7902346	0.005217	0.044072

B3GALT6	JHU17784	1.279637	0.009224	0.025219
B3GALT6	NM_080605	-0.3580302	0.017129	0.018816
B4GALT4	BC004523.2	0.6805267	0.006548	0.047037
B4GAT1	JHU02888	2.0636818	0.011316	0.005411
BAALC	NM_024812.2	0.4252853	0.032307	0.00599
BAD	NM_004322.2	1.3964953	0.01335	0.010146
BAG4	NM_004874.2	-1.8747452	0.026405	0.010567
BAG5	JHU07586	0.1469	0.00631	0.033364
BANP	NM_017869.3	-0.7880709	0.001517	0.003548
BARX1	BC009458.1	0.6609896	0.006637	0.043072
BBS12	NM_152618.2	-0.4550216	0.007145	0.009133
BBS7	JHU10374	0.9028518	0.010046	0.019686
BC011547.1	JHU17241	-0.0527369	0.012348	0.030647
BC025996.2_frag	JHU01778	-2.0250501	0.019428	0.009705
BC030923.1_frag	JHU19665	0.7358425	0.00555	0.048399
BC033035.2_frag	JHU04733	-0.061571	0.053315	0.018337
BC068305.1_frag	JHU19938	0.3017135	0.001463	0.012616
BC073758.1	JHU16427	-0.680512	0.011516	0.037743
BC132930.1_frag	JHU14399	0.4850864	0.008766	0.044993
BCKDHB	BC034481.1	2.1376585	0.012633	0.042191
BCL11A	JHU10659	1.1650666	0.0079	0.02147
BCL2L10	NM_020396.2	1.4861139	0.002545	0.00954
BCL2L12	NM_001040668.1	1.7077986	0.028295	0.007099
BCL2L2	NM_004050.2	-0.9474354	0.009353	0.049505
BCL7A	NM_001024808.1	0.4910174	0.016777	0.042416
BDNF	NM_001709.3	-1.5172253	0.007038	0.049535
BEND2	BC096745.1	2.0137139	0.007104	0.030939
BHLHA9	BHLHA9	1.0635905	0.005793	0.032152
BIN1	BC004101.2	1.2046946	0.006168	0.012009
BIN1	NM_139348.1	-1.7689729	0.006998	0.028454

BMP3	BC117514.1	0.2506745	0.021586	0.0268
BMP7	BC004248.1	-0.8614233	0.00728	0.047426
BOD1	NM_138369.1	-0.4699182	0.017762	0.018229
BOLA2	JHU17509	-0.3303503	0.021548	0.049378
BOLL	NM_197970.1	0.7049884	0.007997	0.042867
BORA	NM_024808.2	0.3497761	0.006538	0.011932
BPNT1	NM_006085.3	1.8081532	0.008183	0.046788
BPY2	JHU17510	1.2310659	0.006722	0.037957
BRAF	JHU19454	-0.2791248	0.002756	0.013737
BRCA1	JHU12826	-0.0693183	0.009649	0.037516
BRD9	NM_001009877.1	0.4938771	0.030098	0.03984
BRE	NM_004899.3	-1.1107683	0.039062	0.023064
BRI3	BC018737.1	-2.1025062	0.020916	0.007339
BRICD5	NM_182563.2	0.0208995	0.006984	0.022513
BT007360.1	JHU14010	0.6699888	0.005567	0.038411
BTN3A2	BC067086.1	1.3925121	0.003635	0.010805
BUD31	NM_003910.2	-0.2203299	0.027584	0.005198
BYSL	BC007340.2	0.4496725	0.00696	0.043753
C10orf35	NM_145306.1	2.6991923	0.007791	0.025655
C10orf62	NM_001009997.1	0.3490589	0.009139	0.046137
C11orf55	NM_207428.1	-1.3229935	0.02041	0.044678
C11orf94	XM_096472.3	0.9049932	0.007016	0.002916
C12orf10	BC051871.1	0.286993	0.018397	0.003583
c12orf32	NM_031465.2	0.6774575	0.00155	0.001699
C12orf42	BC044617.1	1.30463	0.008854	0.03486
C12orf60	BC029443.1	0.9151607	0.017611	0.031259
C12orf66	BC036246.1	-1.0584421	0.031888	0.013497
C14orf1	NM_007176.1	1.7683701	0.007872	0.041277
C14orf28	NM_001017923.1	-0.2311175	0.007614	0.00148
C16orf58	BC009308.2	-0.3812225	0.00466	0.00236

C17orf80	AL136740	0.1612535	0.012762	0.046529
C18orf32	NM_001035005.1	-1.8605483	0.037656	0.008381
C19orf33	NM_033520.1	1.9583194	0.021396	0.042562
C19orf47	NM_178830.2	0.5440332	0.009907	0.018986
C19orf52	NM_138358.2	-1.2384301	0.041549	0.016587
C19orf68	BC043386.1	0.2368336	0.015399	0.009408
C19orf73	NM_018111.1	0.2160563	0.027039	0.022114
C1D	NM_006333.2	-0.3609961	0.012975	0.025082
C1GALT1C1	NM_001011551.1	-0.8805182	0.0413	0.004505
C1orf123	NM_017887.1	-0.4349971	0.005157	0.018739
C1orf131	BC062353.1	1.2363626	0.018374	0.022459
C1orf146	NM_001012425.1	0.8511719	0.010948	0.009967
C1orf177	NM_152607.1	-1.3287194	0.013523	0.018896
C1orf189	NM_001010979.1	1.5150714	0.023132	0.039161
C1orf43	BC000152.2	-0.6119288	0.048251	0.003145
C1QA	NM_015991.2	1.6336115	0.005387	0.016917
C1QTNF3	NM_030945.2	1.0845391	0.014274	0.02246
C1QTNF9	BC040438.1	0.4395902	0.002569	0.022217
C1RL	BC156780.1	0.5767243	0.024557	0.038029
C20orf144	NM_080825.2	1.0146976	0.011508	0.011119
C20orf195	NM_024059.2	0.0027778	0.002846	0.029719
C20orf24	BC004446.1	-0.0449214	0.005868	0.010601
C21orf2	BC031300.1	0.5758413	0.007342	0.01617
C21orf62	JHU15588	0.6486987	0.006209	0.043488
C2orf27A	BC093716.1	-0.7994678	0.010995	0.00848
c3orf35	NM_178338.2	-0.8120877	0.026812	0.03402
C3orf36	NM_025041.2	2.0099645	0.023389	0.027385
C4orf19	BC037906.1	-0.1954044	0.002628	0.049817
C4orf36	NM_144645.1	1.8993712	0.021287	0.044895
C5orf15	NM_020199.1	1.0142831	0.026727	0.036198

C5orf22	BC021215.2	-0.2596923	0.021197	0.044887
c5orf46	NM_206966.1	0.1090262	0.004455	0.03545
C5orf46	NM_206966.1	-0.1008099	0.0695	0.015019
C5orf51	NM_175921.4	0.6927499	0.001582	0.009845
C6orf201	BC047663.1	1.4541351	0.007165	0.01591
C6orf223	NM_153246.2	-0.7409579	0.041772	0.018707
C6orf25	NM_138272.1	-1.7113515	0.00372	0.009267
C6orf48	NM_001040437.1	1.2969524	0.036294	0.04094
C7orf25	BC000769.2	0.4772492	0.010211	0.035778
C7orf61	NM_001004323.1	0.477659	0.012738	0.036167
C7orf62	NM_152706.2	0.0147806	0.054943	0.03708
C8B	NM_000066.2	0.1635975	0.019093	0.045053
C8orf22	NM_001007176.1	0.4608708	0.013919	0.018902
C8orf33	NM_023080.1	0.4632505	0.012649	0.000308
C9	NM_001737.2	-0.5270748	0.02682	0.00298
C9orf163	NM_152571.2	-0.5738902	0.067553	0.020869
C9orf24	NM_032596.3	-1.4735511	0.039354	0.038426
C9orf61	JHU07412	0.0950316	0.004218	0.033218
C9orf89	NM_032310.3	0.8836933	0.014936	0.037913
C9orf9	NM_018956.3	0.605452	0.004489	0.03491
CA3	NM_005181.2	1.2449668	0.00243	0.007563
CA6	NM_001215.2	0.3527316	0.014803	0.044676
CA6	BC034350.1	-1.0062462	0.012533	0.013807
CA7	NM_005182.2	-0.1370937	0.000672	0.03476
CABYR	NM_138644.1	-1.2613416	0.029966	0.007167
CACYBP	NM_014412.2	-0.7988327	0.037309	0.038674
CACYBP	JHU16165	-0.9054105	0.026873	0.029146
CALB1	NM_004929.2	-0.3108941	0.061507	0.026791
CALM1	JHU03859	1.8265911	0.007369	0.034755
CALM1	JHU04532	0.7747063	0.008185	0.001321

CALML6	NM_138705	0.9038736	0.005198	0.013168
CAMKK2	NM_153500.1	0.1386363	0.014072	0.031796
CAMLG	NM_001745.2	0.670498	0.01165	0.038143
CANX	NM_001746.3	-1.9340522	0.008993	0.047837
CAPN1	NM_005186.2	-0.8708491	0.043689	0.034101
Capn15	BC058094	0.6038814	0.007646	0.041116
CARD9	BC008877.2	-1.5932935	0.024797	0.044602
CARHSP1	JHU04533	1.1403927	0.014488	0.010426
CARNS1	BC036557.1	-0.7855948	0.030491	0.011378
CASC4	ENST00000360824	-1.181714	0.036424	0.044491
CASQ2	NM_001232.2	1.440272	0.032859	0.029277
CASS4	BC027951.1	0.20285	0.015736	0.042719
CASZ1	JHU19806	-0.8746236	0.00098	0.024822
CAT	NM_001752.2	-0.9431903	0.012355	0.015227
CATSPER2	BC028728.1	-1.0847557	0.012381	0.001849
CBX3	NM_016587.3	0.9842404	0.003516	0.036928
CBX3	BX648807	0.4085424	0.008359	0.030429
CBX5	NM_012117.1	0.5408919	0.004296	0.015643
CBX7	NM_175709.2	1.5942202	0.009272	0.029185
CBX7	BC051773.1	-1.5321173	0.057306	0.018281
CC2D1A	NM_017721.3	-0.5129565	0.056299	0.004752
CCBL1	BC021262.2	0.7534615	0.018539	0.040073
CCDC107	BC051285.1	0.4927045	0.035843	0.024466
CCDC109B	NM_017918.3	1.1243062	0.005754	0.02776
CCDC113	NM_014157.2	0.6773287	0.006425	0.00034
CCDC149	BC067735	-0.0928132	0.033666	0.028081
CCDC155	BC029811.1	0.3824258	0.013281	0.022107
CCDC185	NM_152610.1	-0.9685793	0.018952	0.040498
CCDC28B	NM_024296.3	1.9093319	0.01306	0.005592
CCDC69	NM_015621.2	-0.4773071	0.080376	0.00311

CCDC73	NM_001008391	-0.5002184	0.036369	0.043227
CCDC74A	NM_138770.1	1.0324891	0.00979	0.03626
CCDC93	BC028609.1	-0.4352772	0.002569	0.047868
CCIN	BC019251.2	-0.3365078	0.022309	0.010533
CCL11	NM_002986.2	-0.1665957	0.004009	0.002741
CCNA1	BC036346	0.7432377	0.004714	0.030418
CCNA2	NM_001237.2	-1.7040797	0.022211	0.030635
CCNE2	NM_057749.1	-1.0947598	0.013161	0.045605
CCNJL	BC013353.2	0.3293401	0.004184	0.033749
CCNY	NM_145012.3	1.4244207	0.005911	0.043147
CCR3	NM_001837.2	-1.9874223	0.115422	0.010832
CCT4	BC106934	1.0813609	0.023393	0.033964
CD19	NM_001770.4	-0.3370684	0.016417	0.012626
CD2	JHU09429	-1.4901459	0.003719	0.017879
CD200R1	NM_138806.3	0.2489678	0.005039	0.046153
CD320	NM_016579.2	-0.2065669	0.007195	0.012575
CD36	NM_000072.2	1.0519323	0.007012	0.035493
CD3D	NM_000732.4	0.3891154	0.011068	0.000211
CD4	NM_000616.3	1.4966751	0.000849	0.00429
CD40LG	BC074950.2	1.1464574	0.009178	0.02316
CD58	BC005930.1	1.3279525	0.003555	0.024754
CD83	BC030830.1	-1.6644571	0.035281	0.043337
CDC123	BC001600.1	0.5718518	0.007432	0.026329
CDC16	NM_001078645.1	0.1669201	0.019192	0.0067
CDC20B	BC037547.1	-0.9643395	0.035729	0.047403
CDC23	BC017713.1	2.065934	0.006571	0.01493
CDC42BPA	CDC42BPA	0.5001581	0.002108	0.014387
CDCA3	NM_031299.3	-0.1914883	0.052343	0.034657
CDCA5	NM_080668.2	-0.259142	0.019221	0.018193
CDCA7	NM_145810.2	1.0035806	0.010688	0.035431

CDH10	NM_006727	0.4740127	0.007539	0.031632
CDH12	NM_004061.2	0.676622	0.003454	0.026847
CDH13	NM_001257.3	0.6080981	0.030102	0.028503
CDIP1	NM_013399.2	-0.4067946	0.069641	0.001304
CDIPT	JHU05013	0.4766402	0.044018	0.039228
CDK17	NM_002595.2	-0.9013701	0.006403	0.028094
CDK5RAP3	NM_176095.1	0.2718216	0.013738	0.007819
CDK9	NM_001261.2	0.0716664	0.039764	0.018626
CDKL5	JHU07598	0.432714	0.014817	0.023433
CDNF	BC037872.1	0.1188947	0.003055	0.010687
CDSN	BC031993	0.1573524	0.013728	0.044749
CEACAM19	BC083499.1	0.7700716	0.027198	0.028979
CELA2A	BC069331.1	-0.5812223	0.008205	0.011008
CELA3A	BC005918.1	0.941037	0.00751	0.026273
CELF4	BC001946.1	0.2824718	0.024618	0.037694
CELF6	BC030835.1	-0.6633533	0.050285	0.026553
CENPI	BC012462.2	-2.2574258	0.024871	0.030345
CENPQ	JHU13423	-0.0077929	0.011806	0.026802
CENPT	NM_025082.3	-0.6835926	0.005857	0.02876
CENPV	BC052604.1	-0.4736219	0.034613	0.015928
CEP126	JHU17997	0.3157229	0.011711	0.011272
CEP290	BC008641.1	1.032974	0.023784	0.041687
CFH	JHU07599	0.9329522	0.01133	0.022402
CGB	JHU03672	-1.3834833	0.023024	0.033334
CH17-5A7.1	JHU12401	2.357869	0.000599	0.001553
CH25H	JHU04335	0.2994143	0.021637	0.031911
CHAC1	NM_024111.2	0.3746193	0.005798	0.022969
CHGB	NM_001819.1	0.8551549	0.002371	0.000985
CHMP1A	NM_002768.4	1.817362	0.016492	0.044356
CHMP3	NM_016079.2	-0.186169	0.003016	0.01728

CHMP4B	NM_176812.3	-0.8991835	0.057049	0.001066
CHMP6	NM_024591.3	0.9083316	0.026089	0.03312
CHN1	NM_001822.3	0.4596807	0.011704	0.049905
CHORDC1	BC072461.1	1.3536293	0.005284	0.016555
CHPF2	NM_019015.1	0.386554	0.002988	0.023048
CHRDL1	ENST00000372045	1.5112421	0.110447	0.013267
CHRM5	BC041805.1	-0.1348055	0.011984	0.036937
CHRNB3	NM_000749.3	0.0377585	0.01772	0.003488
CHRNE	NM_000080	1.7181168	0.004947	0.023396
CHST13	BC103895.1	0.0715488	0.0067	0.006208
CHST4	NM_005769.1	-1.5503453	0.037498	0.041079
CHST7	BC045537	0.4650953	0.003579	0.033426
CIZ1	BC021163.2	1.5026506	0.02541	0.038701
CKAP2	NM_018204.2	-0.2873677	0.01771	0.017206
CKLF	NM_181640.1	-0.0691258	0.031575	0.027532
CKS2	NM_001827.1	2.1814718	0.009855	0.016073
CLASRP	BC013178.1	-0.9990049	0.015026	0.049969
CLCA2	JHU07600	0.9967008	0.017298	0.015948
CLDN18	NM_016369.3	1.5110066	0.006374	0.016996
CLDN8	NM_199328.1	-0.2035132	0.005385	0.013185
CLEC12A	NM_201625.1	0.2457284	0.007067	0.001447
CLEC1B	BC029554.1	-0.3771882	0.033704	0.048117
CLEC2D	NM_001004419.2	-0.6235235	0.019794	0.041316
CLEC4M	BC038851	0.5767204	0.002984	0.016433
CLEC5A	NM_013252.2	-1.4009243	0.028157	0.037971
CLIC2	NM_001289.4	-1.4511467	0.002173	0.007293
CLIC3	NM_004669.2	0.6754968	0.009627	0.033978
CLK2	NM_003993.2	-0.2072678	0.005297	0.034972
CLPTM1	BC004865.2	0.3266083	0.020412	0.044819
CLVS1	JHU07639	-1.133915	0.0106	0.049497

CLYBL	BC034360.1	0.6492478	0.00558	0.013868
CMA1	BC103974.1	-0.6306746	0.007324	0.044607
CMC4	BC002600	1.8452426	0.01783	0.048035
CMSS1	NM_032359.2	1.369604	0.006267	0.010931
CMTM2	NM_144673.2	0.8034972	0.013736	2.49E-05
CMTM3	ENST00000361909	0.4784458	0.00627	0.048336
CNBP	BC093058.1	-0.3444546	0.004748	0.023627
CNGB1	NM_001297	0.9915341	0.002449	0.041532
CNIH2	NM_182553.1	-0.0723594	0.025654	0.020818
CNOT7	NM_054026.1	0.4435078	0.008983	0.038121
CNTF	NM_000614.2	0.1159397	0.002675	0.022366
CNTLN	NM_017738	1.8176388	0.025237	0.026487
COA3	NM_001040431.1	-0.3620709	0.023486	0.049798
COBLL1	BC006264.2	-0.4507653	0.023987	0.010055
COL10A1	NM_000493.3	1.5472997	0.006209	0.034709
COL18A1	NM_130445.2	0.0332465	0.012067	0.019255
COL21A1	NM_030820.3	1.909776	0.015552	0.013492
COL2A1	JHU02126	-0.8057886	0.112459	0.025452
COLCA2	XM_071712.11	-0.9184454	0.0093	0.04052
COLEC12	NM_030781.2	2.0606256	0.009629	0.032134
COMMD4	NM_017828.3	0.8325719	0.034208	0.006499
COMMD7	BC022073.1	-0.1117743	0.027233	0.029351
COMT	NM_007310.1	1.3419694	0.002082	0.003319
COPS3	NM_003653.2	-1.5777909	0.020152	0.01442
COPS5	NM_006837.2	-0.8596203	0.021279	0.002741
COQ10A	NM_001099337.1	1.038874	0.013018	0.014396
COX18	NM_173827.2	-0.2905956	0.026099	0.027958
COX4I1	BC047869.1	-0.3237548	0.008035	0.043879
COX7A2L	NM_004718.2	1.8988487	0.014669	0.033079
COX8A	NM_004074.2	0.6901148	0.033898	0.026792

CPA2	BC014571.1	0.3814754	0.00791	0.036153
CPA3	NM_001870.1	-2.3391708	0.067973	0.006168
CPLX1	NM_006651.3	-1.619468	0.008965	0.004571
CPNE1	NM_003915.2	0.5348516	0.0193	0.001797
CPNE3	BC036242.1	0.1729182	0.006521	0.022193
CPNE4	NM_130808.1	-1.8600748	0.012886	0.003665
CPO	NM_173077.2	-1.2821936	0.006583	0.032097
CPOX	BC023554.2	1.0763278	0.007739	0.015373
CPOX	BC017210.2	-0.5559201	0.024918	0.005973
CPQ	NM_016134.2	0.1852663	0.005469	0.019128
CRABP2	NM_001878.2	-0.4780432	0.006248	0.048061
CREB3L1	BC014097.1	-0.5194207	0.024452	0.015235
CREB5	NM_182899.3	-0.7680436	0.082648	0.000939
CREBL2	NM_001310.2	0.435066	0.013113	0.048288
CREBRF	BC041709.1	1.2607487	0.018713	0.024186
CREG2	NM_153836.3	0.1234648	0.006525	0.024443
CRELD2	BC002894.1	1.6874988	0.004841	0.028018
CREM	NM_182718.1	1.3091378	0.02246	0.005102
CREM	NM_182770.1	0.683757	0.014084	0.023805
CRIP3	NM_206922	0.1378572	0.006405	0.047664
CRIPT	NM_014171.3	1.0360307	0.008562	0.014174
CROCCP2	BC047736.1	1.5510762	0.035513	0.022153
CROCCP3	BC033082.1	0.2387722	0.010577	0.02614
CRYBA4	NM_001886.1	-0.857003	0.002659	0.035209
CRYBB3	BC102021.1	-0.0580757	0.014131	0.037885
CRYGB	CRYGB	-0.1694546	0.03939	0.019017
CRYM-AS1	JHU11073	0.0966706	0.045044	0.025154
CSAG1	BC059947.1	0.455213	0.010121	0.018669
CSAG1	NM_153479.1	-0.3496441	0.006043	0.04982
CSAG2	JHU03870	-0.0350692	0.00167	0.029334

CSF2	BC113999.1	0.1349155	0.006993	0.044657
CSN2	NM_001891.2	-0.1201961	0.0161	0.016545
CSNK1G1	ENST00000303032	0.8864615	0.033897	0.025676
CSRP2BP	NM_020536.2	0.1855329	0.00571	0.001602
CSRP3	NM_003476.2	-0.1101642	0.008523	0.030409
CST2	NM_001322.2	-1.3543256	0.030084	0.004213
CSTA	NM_005213.3	0.7776093	0.012819	0.041357
CSTF2	NM_001325.2	0.8164943	0.009933	0.017728
CT45A3	JHU03338	1.705842	0.005839	0.036556
CTAGE5	CTAGE5	1.115604	0.015255	0.026426
CTH	NM_001902.4	0.0903732	0.016555	0.02952
CTPS2	NM_019857.3	-0.055702	0.023179	0.040551
CTSK	NM_000396.2	-1.576428	0.036279	0.003698
CU689359.1_frag	JHU05341	0.0955863	0.033701	0.012291
CU692086.1_frag	JHU07258	1.3909962	0.02637	0.013293
CUL4A	NM_001008895.1	0.2441213	0.002967	0.002604
CUL4A	NM_003589.2	-0.0252668	0.018109	0.028536
CWC25	NM_017748.3	0.7939047	0.022104	0.00534
CWF19L1	BC008746.2	0.649891	0.004763	0.02278
CXCL12	P48061	-0.3513844	0.035718	0.044715
CXCL2	NM_002089.1	2.6483546	0.000633	0.002984
CXorf40A	CXorf40A	1.8398087	0.015885	0.003633
CXXC1	BC014940.2	-0.2349034	0.034094	0.033137
CXXC4	NM_025212.1	-0.5567107	0.010332	0.00439
CYB561A3	BC047710.1	2.1733591	0.01136	0.0043
CYBRD1	BC065290.1	0.738022	0.009388	0.023106
CYP27A1	NM_000784.2	0.0109142	0.001026	0.008123
CYP2B6	BC067431.1	1.356462	0.015114	0.002982
CYP2C18	NM_000772.1	1.5455996	0.002308	0.001613
CYP2E1	BC067433.1	1.4705058	0.008878	0.045495

CYP2J2	BC032594	0.338669	0.007907	0.006111
CYP2R1	NM_024514.4	-0.0100557	0.005737	0.040794
CYP4A11	BC041158.1	1.5352005	0.021941	0.030949
CYSLTR1	NM_006639.2	0.0007153	0.011627	0.016304
CYTH2	BC038713.1	-1.5670166	0.013651	0.02992
CYTH4	NM_013385.2	-1.448074	0.040729	0.006944
DAB1	BC067447.1	-0.3746214	0.00381	0.020944
DAB1	BC067445.1	-0.6067397	0.014426	0.01721
DAPK3	NM_001348.1	0.1790785	0.005128	0.033933
DBF4	NM_006716.3	1.8827656	0.022566	0.025833
DBN1	BC007567.1	-0.1025571	0.000407	0.015866
DBNL	NM_014063.5	0.2543716	0.004678	0.041194
DBP	NM_001352.1	0.6545763	0.008661	0.041728
DCDC2	Q9UHG0	-0.7497272	0.007159	0.003336
DCUN1D2	ENST00000375399	1.6773943	0.005686	0.01617
DCUN1D2	NM_001014283.1	-0.3770243	0.061196	0.00729
DDHD2	BC010504.1	-1.6886226	0.005499	0.009046
DDX26B	NM_182540.2	0.4688554	0.018306	0.02449
DDX31	NM_138620	1.779355	0.015769	0.022901
DDX39A	NM_005804.2	-0.9889966	0.012922	0.014533
DDX50	NM_024045.1	1.9383072	0.009819	0.006247
DDX55	BC035911.1	-0.2803072	0.006909	0.032498
DEAF1	NM_021008.2	-0.7641848	0.014644	0.042839
DECR1	NM_001359.1	0.1720818	0.010866	0.015092
DECR2	NM_020664.3	0.8129908	0.013067	0.021661
DEFB1	NM_005218.3	-0.0768506	0.001919	0.039704
DEFB107A	NM_001037668.1	0.1006751	0.006644	0.045495
DEPDC1B	BC010904.1	-1.3929383	0.021447	0.043629
DERL1	NM_024295.3	0.5518039	0.023735	0.045042
DFFB	NM_001004285.1	0.100711	0.005301	0.049443

DGCR6L	BC000682.2	-2.3931472	0.015662	0.004047
DGKB	NM_145695.2	-0.4711226	0.010209	0.045004
DHFR	JHU02454	-0.7517201	0.012719	0.018769
DHPS	NM_001930.2	-0.7804162	0.007644	0.010653
DHRS3	NM_004753.4	1.3298679	0.003006	0.012328
DHRS4	NM_021004.2	-0.1961916	0.010109	0.00238
DHRS4L2	BC000663.2	1.0406132	0.057169	0.001336
DHRS9	BC051797.1	0.2472905	0.002899	0.046405
DHRS9	NM_005771.3	-0.5056618	0.037535	0.006638
DHTKD1	NM_018706.4	1.1041395	0.006863	0.033105
DLX5	NM_005221.5	0.2482767	0.021419	0.0098
DMGDH	NM_013391	0.4666086	0.005739	0.031217
DMTN	BC006318.2	0.5004162	0.011417	0.034543
DNAJB3	NM_001001394.2	1.6697203	0.0123	0.035564
DNAJC12	NM_201262.1	1.8150427	0.005178	0.005389
DNAJC5	NM_025219.1	-0.121775	0.004361	0.030308
DNAJC6	NM_014787.2	0.4716465	0.010701	0.024136
DNASE1L2	NM_001374.2	0.5468317	0.003167	0.012755
DNASE2	NM_001375.2	-1.2064397	0.002017	0.016846
DNMT3B	BC111933	1.7613993	0.014677	0.019327
DNTT	BC012920.1	-0.6773306	0.01917	0.02675
DOCK2	BC016996.1	1.0286357	0.001645	0.011868
DOK2	BC032623	0.048399	0.011622	0.02424
DOK6	NM_152721.2	-0.4539901	0.034052	0.039545
DOLPP1	NM_020438.3	1.6064257	0.003284	0.015852
DPEP1	BC017023	0.2687417	0.004933	0.013227
DPEP2	BC024021.1	0.4377912	0.010662	0.023996
DPP10	BC030832.1	0.6335134	0.010741	0.003144
DPP3	NM_005700.3	0.2158468	0.00514	0.037153
DPP3	BC001446.2	-0.4789684	0.011385	0.018406

DPP9	BC037948	0.5737472	0.028528	0.030068
DPPA3	NM_199286	1.4104062	0.015858	0.047938
DRG2	NM_001388.3	0.8680101	0.011335	0.045643
DSC1	NM_024421	0.109186	0.001003	0.011265
DSN1	BC026011.1	1.0190057	0.008703	0.02078
DTNA	NM_032975.2	0.2346188	0.006188	0.037569
DTWD1	BC093073.1	0.4990038	0.006327	0.011945
DTWD1	ENST00000329873	0.3719569	0.015003	0.035111
DUOXA1	BC020841.1	0.0527893	0.006766	0.035244
DUS2	BC006527	1.8159562	0.023419	0.045676
DUSP10	NM_007207.3	2.0852293	0.008206	0.045144
DUSP18	BC028724.1	0.3993511	0.000866	0.004371
DUSP22	NM_020185.3	0.0979311	0.004179	0.034016
DUSP22	BC016844.1	-0.1266106	0.002349	0.030894
DUSP23	NM_017823.3	2.0911494	0.005686	0.028552
DUSP6	JHU02133	1.5827799	0.027129	0.028589
DUSP8	NM_004420.2	2.022328	0.04116	0.007431
DYNC1I1	BC037854.1	-1.034446	0.007304	0.002387
DZIP3	BC063882	1.0142155	0.003166	0.02074
E2F6	NM_198325.1	0.5375929	0.007187	0.00637
E2F6	NM_212540.1	0.378314	0.00124	0.022805
Ebf2	BC050922	-0.195875	0.009113	0.018949
EBI3	NM_005755.2	0.7727581	0.061887	0.002567
EBPL	BC018478	0.6651132	0.002735	0.016718
ECE1	NM_001397.1	-0.9235628	0.057502	0.042271
ECHDC1	BC003549.1	0.868201	0.055583	0.034587
ECI2	NM_206836.1	0.1020434	0.028703	0.02225
EDAR	CR749352	0.6008693	0.010704	0.008815
EDC3	NM_025083.2	3.1424687	0.025718	0.034759
EDC4	JHU14195	1.2499404	0.041852	0.005678

EDN2	NM_001956.2	1.0911575	0.006822	0.034749
EDNRA	ENST00000324300	-0.9641874	0.001154	0.04514
EED	NM_003797.2	-2.345522	0.009014	0.028284
EEF1B2	NM_001959.3	0.9397518	0.003339	0.011102
EEF1G	BC013918.1	1.6440715	0.006627	0.018932
EEF2KMT	JHU08669	2.4204226	0.011722	0.044986
EEFSEC	BC007933	0.3926266	0.019311	0.048612
EFEMP1	NM_004105.3	0.4191605	0.002473	0.004675
EFHB	NM_144715.2	0.416898	0.005592	0.011579
EFNB2	NM_004093.2	2.2441639	0.012801	0.040489
EFNB3	NM_001406.3	1.7200394	0.010899	0.019276
EFNB3	NM_001406.3	0.5410865	0.003038	0.02372
EFS	NM_032459.1	0.208246	0.028995	0.01076
EGLN3	NM_022073.2	0.8246001	0.027599	0.022086
EIF1AX	NM_001412.3	1.3401374	0.031559	0.025037
EIF2B1	NM_001414.2	0.8814373	0.010961	0.036036
EIF2B2	BC000494.2	0.4409302	0.028775	0.037427
EIF2B3	JHU13523	1.4981153	0.01355	0.040698
EIF2D	NM_006893.2	0.551015	0.019944	0.039269
EIF3K	NM_013234.2	0.9131467	0.010654	0.03475
EIF4A3	NM_014740.2	0.7208406	0.02103	0.047681
ELF2	NM_006874.2	-0.4139107	0.029002	0.044603
ELOF1	NM_032377.3	1.4633413	0.001484	0.006827
ELP6	BC000623.2	1.7061452	0.012707	0.041119
EMC1	NM_015047.1	-0.7558832	0.005081	0.038341
EMC2	NM_014673.2	0.3768612	0.032485	0.045157
EMCN	BC017781.1	1.4382601	0.006334	0.033183
EME1	BC016470.2	-0.5917275	0.00409	0.012734
EMR4P	NM_001080498	0.3973518	0.025648	0.032655
ENDOU	NM_006025.3	0.8441869	0.033564	0.019997

ENHO	BC022101.1	0.1882975	0.005406	0.015742
ENSA	NM_004436.2	0.8681661	0.019948	0.032506
ENTPD1	NM_001776.3	0.8998528	0.007401	0.004455
ENTPD8	NM_001033113.1	2.1098775	0.037679	0.032552
EP400NL	BC066974.1	0.9851148	0.006549	0.028246
EPB41L2	BC034718.1	-0.2704982	0.010502	0.01579
EPB41L3	BC006141.1	0.6665702	0.004261	0.008513
EPB42	BC096093.4	0.865538	0.002293	0.003929
EPDR1	BC018299.1	1.096939	0.029707	0.004081
EPHA4	BC016981.1	-1.6221644	0.011211	0.000576
EPHX2	NM_001979.4	0.2690455	0.00474	0.025995
EPM2A	BC005286.1	-0.2960558	0.036732	0.039611
EPN3	BC001038.2	0.0302499	0.005125	0.021365
EPS8L2	NM_022772.2	1.1149882	0.019573	0.019483
EPSTI1	NM_001002264.1	0.2095375	0.015355	0.038146
ERCC5	NM_000123.2	1.0398901	0.006911	0.007706
ERG	NM_004449.3	1.8639173	0.006003	0.027833
ERG	NM_182918.2	0.5017446	0.005301	0.026802
ERLIN2	NM_007175.5	1.121034	0.013418	0.036021
ERMP1	BC031630.1	2.0483502	0.028406	0.003511
ESD	BC001169.1	-0.1775245	0.002091	0.019813
ETFA	NM_000126.2	2.2958475	0.007353	0.007577
ETS1	NM_005238.2	2.3899587	0.003233	0.004987
ETS1	BC017314.2	0.1494556	0.023269	0.008904
EU832464.1	JHU18572	1.0305561	0.033945	0.044667
EVX2	EVX2	0.2359395	0.001901	0.003284
EXO5	NM_022774.1	1.6708825	0.012667	0.020506
EXOC3-AS1	JHU03026	0.9316254	0.00112	0.002258
EXOC6B	NM_015189	-0.077807	0.017916	0.047468
EXOG	NM_005107.1	0.7238687	0.007503	0.042316

EXOG	JHU10873	0.470021	0.012997	0.024345
EXT2	NM_000401.2	-0.1925192	0.005353	0.003313
EZH1	NM_001991.2	-0.6541232	0.017167	0.042651
F12	BC012390.1	0.0145618	0.006143	0.028964
F13B	NM_001994.2	0.0644915	0.015058	0.033812
F2R	JHU07609	-1.1314027	0.000974	0.004828
F7	NM_019616.1	1.6201402	0.003715	0.020004
F8A1	BC039693	0.9022269	0.002322	0.01557
FAAH2	NM_174912.2	0.1711999	0.04624	0.048193
FABP9	NM_001080526	1.3936848	0.00464	0.042479
FAM102B	NM_001010883.1	0.3935168	0.009088	0.005495
FAM104B	NM_138362.1	0.9441033	0.025871	0.026752
FAM119A	BC009462.1	0.4553542	0.019937	0.016682
FAM129A	NM_052966.2	-0.7860004	0.00064	0.028875
FAM134C	NM_178126.2	-0.1404828	0.021876	0.027618
FAM160A2	BC030825.1	0.0419558	0.017598	0.012593
FAM188A	NM_024948.2	0.6786883	0.009384	0.024528
FAM189B	ENST00000487649	0.3881488	0.014728	0.016505
FAM210A	NM_152352.2	1.6572575	0.027077	0.040409
FAM214A	BC040548.1	0.9013386	0.012279	0.031887
FAM220A	BC006110.1	-0.5031206	0.003335	0.017861
FAM26D	NM_153036.2	0.238829	0.012002	0.013597
FAM27E3	XM_001720463.3	0.1173037	0.007904	0.026198
FAM35A	BC051863.2	1.3084773	0.015155	0.017395
FAM46B	NM_052943.2	-1.372726	0.010709	0.002314
FAM57A	BC026023.1	-0.9420188	0.028303	0.027099
FAM63A	NM_018379.3	-0.1483796	0.002385	0.006273
FAM71A	NM_153606.1	-0.9114509	0.012578	0.010937
FAM71C	NM_153364.1	0.901272	0.001875	0.009741
FAM72B	XM_935477.2	0.3685633	0.0121	0.022601

FAM81A	NM_152450.1	1.6502691	0.031811	0.027116
FAM81B	FAM81B	0.4467758	0.005026	0.041875
FAM89B	NM_152832.1	0.5409092	0.003295	0.003207
FAM92A1	BC014598.2	0.2154822	0.053979	0.040018
FANCD2OS	NM_173472.1	0.089822	0.001689	0.016442
FARSB	BC017783.1	1.4625355	0.007479	0.028143
FAS	NM_152872.1	0.0692111	0.009807	0.01693
FASTKD2	JHU00999	0.0813289	0.046125	0.04888
FATE1	NM_033085.2	0.9198964	0.012133	0.016987
FBRS	BC014212.1	0.9656067	0.005999	0.045049
FBXL13	BC026121.1	1.1218569	0.036314	0.040637
FBXL20	NM_032875.1	0.0503954	0.003555	0.016773
FBXL4	NM_012160.3	-0.748738	0.027139	0.03441
FBXO15	NM_152676.1	-0.7171555	0.027323	0.034598
FBXO16	BC102028	0.0470313	0.061313	0.010849
FBXO22	NM_012170.2	-0.2175575	0.017224	0.002741
FBXO27	NM_178820.3	-1.1744224	0.040712	0.007181
FBXO31	BC012748.1	-1.8120762	0.045262	0.042734
FBXW12	BC101304.1	0.5363787	0.006674	0.034594
FBXW5	ENST00000371641	-1.8579085	0.01924	0.049761
FCAR	NM_002000.2	0.2792359	0.000636	0.007071
FCGR1A	NM_000566.2	-0.4907417	0.004646	0.001996
FCGRT	BC008734	0.6954917	0.006846	0.004325
FCHSD2	NM_014824.1	-0.0349477	0.006735	0.045718
FCRLA	NM_032738.3	0.4883121	0.010523	0.044365
FER	FER	0.3245323	0.00754	0.001408
FERMT3	NM_031471.4	-1.1842398	0.02231	0.022827
FFAR3	BC035657	0.2134502	0.037212	0.015679
FGD3	BC032429.1	1.4425069	0.035152	0.035602
FGF12	NM_004113.3	-1.5797072	0.001706	0.0019

FGF16	NM_003868.1	1.0938356	0.008748	0.020129
FGF17	FGF17	1.0764873	0.012148	0.007975
FGFR2	NM_022970.2	1.783147	0.023017	0.005263
FGFR2	NM_000141.3	1.08783	0.003903	0.031334
FIGNL2	NM_001013690	0.7069401	0.002418	0.016095
FIP1L1	BC017724.1	-2.2271607	0.004572	0.037859
FITM1	BC042179.1	1.3222887	0.00225	0.014994
FKBP1B	NM_054033.1	2.5453285	0.00733	0.031392
FKBP7	NM_181342.1	1.6681794	0.010553	0.010514
FLI1	NM_002017.2	0.791038	0.004055	0.014719
FLJ45831	NM_001001684.1	0.6063636	0.026652	0.00866
FLRT2	FLRT2	0.515844	0.008535	0.023089
FMNL1	BC021906.1	-1.2203306	0.021269	0.040183
FMNL3	BC033181.1	1.0813597	0.018188	0.036639
FMO1	FMO1	1.0321234	0.010852	0.021265
FOPNL	NM_144600.1	2.1908309	0.007319	0.009633
FOS	NM_005252.2	-1.5857128	0.016177	0.048048
FOSL1	NM_005438.2	0.608984	0.008241	0.010741
FOXC2	JHU19731	0.2916123	0.08949	0.005457
Foxd1	Foxd1	1.0210373	0.007645	0.019015
FOXF1	FOXF1	-0.6463912	0.032093	0.001007
FOXF2	NM_001452.1	1.3330369	0.00562	0.033999
FOXJ2	NM_018416.2	-0.9509288	0.055772	0.005092
FOXL1	BC117226	1.6560014	0.005476	0.036606
FOXM1	NM_202003.1	0.2234764	0.00386	0.032848
FOXN3	NM_005197.2	1.419693	0.044191	0.006715
FOXP2	BC018016.1	0.7608886	0.025352	0.007435
FPR1	NM_002029.3	-2.1546294	0.086476	0.039027
FRAT2	BC020165.1	1.1087618	0.040204	0.021235
FSCN2	FSCN2	0.6325369	0.007991	0.033519

FSIP1	JHU07906	1.4109608	0.016008	0.015348
FSIP1	JHU07906	0.8476492	0.014137	0.00982
FSIP1	JHU07906	0.1767819	0.003256	0.023871
FTSJ2	NM_013393.1	0.0406158	0.000504	0.010868
FUT10	BC063462.1	-0.2177808	0.017414	0.020253
FUT8	NM_178154.1	-0.2049669	0.035925	0.018228
FUZ	NM_025129.3	0.2271436	0.011761	0.040254
FXN	JHU07331	-0.1634868	0.012105	0.046554
FXYD5	BC009642.2	0.4754646	0.024774	0.023223
FXYD7	NM_022006.1	-1.5935975	0.007643	0.032806
FZD4	JHU04348	0.0500781	0.006926	0.006565
G3BP2	NM_203504.1	-0.2456133	0.000578	0.010818
G6PC	NM_000151.1	-0.3240901	0.057719	0.021545
GAB2	NM_080491.1	1.877358	0.008463	0.021228
GABARAPL1	NM_031412.2	0.781817	0.069605	0.049891
GABBR1	BC041332.2	-0.3313003	0.053251	0.048849
GABPB1	BC036080.1	1.2190005	0.008259	0.000445
GABRA1	NM_000806.3	1.3270131	0.032011	0.00346
GABRA6	NM_000811.1	1.2495205	0.018798	0.017827
GABRB3	NM_021912.3	0.6604972	0.003886	0.029578
GABRG1	BC031087.1	0.4772939	0.003003	0.003614
GAGE1	NM_001468.3	-0.9304424	0.003831	0.042551
GALM	NM_138801.1	2.1832582	0.012063	0.028084
GALNT12	BC013945.1	-0.599938	0.005432	0.034327
GAN	NM_022041.2	-0.0278024	0.00165	0.022229
GAS2L3	NM_174942.1	0.0664576	0.02749	0.038962
GAS6	NM_000820.1	-0.5742183	0.029087	0.011529
GAST	NM_000805.3	0.1616336	0.010401	0.00169
GATA3	NM_002051.2	0.6284381	0.012664	0.037406
GATAD1	NM_021167.3	0.4722099	0.005844	0.007614

GBP4	JHU13051	0.3516891	0.008383	0.048689
GBP4	BC070055.1	0.1030673	0.00427	0.04018
GBP7	NM_207398	0.0217337	0.039172	0.047682
GCHFR	NM_005258.2	-0.0093527	0.010669	0.014709
GCSAML	NM_145278.1	0.5230616	0.009798	0.041817
GCSH	NM_004483.4	0.6305365	0.010707	0.015582
GDPD5	NM_030792.4	-0.1342774	0.031136	0.034893
GFM2	NM_032380.3	1.1841013	0.010098	0.044579
GFRA1	NM_005264.2	0.2964666	0.039791	0.045507
GGCT	NM_024051.2	1.5034011	0.011413	0.028103
GGCT	JHU00400	0.2395581	0.015004	0.024333
GIMAP6	NM_024711.3	1.6368219	0.003369	0.012489
GJB3	NM_001005752.1	-0.6515036	0.02992	0.014427
GKN1	BC059778.1	-1.0813609	0.086158	0.025268
GLO1	NM_006708.2	-0.2783078	0.015991	0.00258
GLT6D1	NM_182974.2	0.5362041	0.006554	0.019887
GLYATL2	BC021682.1	0.4074726	0.037531	0.048925
GM2A	NM_000405.3	0.5265081	0.008163	0.026799
GMPPA	NM_013335.2	-2.0297834	0.001509	0.000944
GMPR	NM_006877.2	0.9332248	0.006947	0.026587
GMPR2	NM_001002000.1	2.1734004	0.03933	0.044011
GMPS	NM_003875.2	0.0224446	0.005984	0.027405
GNAI1	NM_002069.4	0.3049529	0.006188	0.011508
GNB5	BC011671.2	-1.7031379	0.017226	0.034329
GNE	NM_005476.3	-0.4533222	0.013749	0.039103
GNG2	NM_053064.2	2.3887736	0.025991	0.016196
GNG3	BC015563	1.4236626	0.007242	0.025879
GNL3L	NM_019067.4	-1.2785833	0.040182	0.043806
GNPAT	NM_014236.2	1.9636486	0.006074	0.010512
GNPAT	BC000450.2	1.5463787	0.006725	0.011337

GNPNAT1	NM_198066.2	-0.2214882	0.023713	0.037749
GOLM1	NM_016548.2	-0.3377107	0.064047	0.044294
GORAB	BC047476.1	0.1825075	0.002484	0.001032
GORASP2	NM_015530.3	2.0726563	0.004981	0.020153
GOSR2	NM_004287.3	-1.7712707	0.001757	0.040916
GPAM	BC030783.1	-1.7998888	0.017971	0.003843
GPANK1	NM_033177.2	-1.5378972	0.008961	0.024823
GPC5	NM_004466.3	0.3806203	0.020464	0.047145
GPER1	BC082766.1	0.623066	0.03932	0.044405
GPER1	NM_001505.2	0.3010929	0.02986	0.040345
GPR17	BC031653.1	0.3162065	0.004935	0.040422
GPR21	NM_005294.1	-1.6767131	0.006924	0.040921
GPR56	BC008770.2	0.5218698	0.013059	0.033713
GPR63	BC067468.1	1.1220492	0.00548	0.000697
GPR75	NM_006794.1	-0.1597837	0.017484	0.02852
GPR78	NM_080819.2	-1.0946975	0.00158	0.003522
GPR85	NM_018970.3	-0.6115485	0.005353	0.031627
GPSM3	NM_022107.1	1.0730202	0.001487	0.008856
GPX6	NM_182701	0.4912702	0.013761	0.01226
GRAMD2	NM_001012642	1.2080279	0.004674	0.045866
GRAP2	NM_004810.2	0.4666837	0.021888	0.047351
GRB2	NM_002086.3	0.1170083	0.027138	0.006332
GRHL3	NM_198173.1	1.311789	0.013555	0.021051
GRHL3	BC036890.2	-1.3394013	0.03156	0.02843
GRIN3A	NM_133445.1	0.346312	0.004614	0.032763
GRK7	GRK7	-0.2367815	0.013805	0.002629
GRM7	NM_000844.2	0.8666994	0.00519	0.035469
GRPEL2	NM_152407.3	0.1232855	0.006852	0.025177
GTF2A1L	NM_006872.2	0.5802632	0.018003	0.024715
GTF3C3	NM_012086.1	0.9308989	0.007442	0.039154

GTF3C5	BC017337.2	0.8014388	0.024546	0.04694
GTPBP8	ENST00000295864	-1.1409908	0.025698	0.040566
GTSF1	NM_144594.1	1.7030554	0.007635	0.048839
GTSF1L	NM_176791.3	-0.8123186	0.027704	0.046671
GUCA1C	BC103993.1	1.0293793	0.015968	0.037485
GUCY2F	NM_001522.2	0.819092	0.076195	0.013775
GUF1	BC012338.1	-0.030614	0.034446	0.010289
GXYLT1	BC039145.1	0.4178502	0.025545	0.00565
GYG2	BC023152.1	0.9778579	0.007739	0.006219
H1F0	BC029046.1	-2.3730368	0.023187	0.008213
H2AFB2	JHU16861	1.6150069	0.013462	0.046586
H2AFV	NM_012412.3	1.7153829	0.00732	0.042185
H2AFV	NM_201516.1	1.488881	0.005548	0.004822
HADHA	JHU12664	0.0092397	0.006437	0.045851
HAO1	NM_017545.2	0.6854643	0.004944	0.005184
HAS3	NM_005329.2	0.9000488	0.037982	0.045137
HAUS6	NM_017645.3	0.4778971	0.035145	0.00816
HBG1	NM_000559.2	2.509311	0.006217	0.005413
HCCS	BC001691.2	0.243169	0.038038	0.001453
HDAC6	BC013737.1	-0.1093695	0.015782	0.012536
HELLS	NM_018063.3	0.1216401	0.018158	0.017272
HENMT1	BC012198.1	-0.8188128	0.008028	0.026835
HES6	NM_018645.3	-0.4354278	0.004805	0.048253
HESX1	JHU09729	0.5082185	0.007265	0.044369
HESX1	JHU09729	0.2418154	0.007389	0.048069
HESX1	NM_003865.1	-0.4396197	0.013303	0.032956
HEYL	NM_014571.3	1.1683844	0.080796	0.036189
HFE	NM_139010.2	2.5339823	0.004784	0.036982
HG492309.1_frag	JHU14995	0.0717013	0.006966	0.009931
HG496653.1_frag	JHU14519	-0.0366774	0.016097	0.023359

HG503526.1_frag	JHU01684	-0.6603421	0.011212	0.02407
HGS	NM_004712.3	0.8478144	0.013038	0.003531
HHLA3	NM_001031693.2	0.3310454	0.007305	0.009903
HIP2	NM_005339.3	2.1123501	0.009519	0.04899
HIPK2	NM_022740	0.4292533	0.032189	0.022914
HIPK2	HIPK2	0.3762398	0.028857	0.02097
HIPK4	NM_144685.3	0.7267896	0.011135	0.022508
HIST1H2AB	JHU18621	0.5587965	0.006747	0.015585
HIST1H2AG	BC016677	0.7219592	0.010453	0.044095
HIST1H2AJ	NM_021066.2	0.883748	0.005152	0.006747
HIST1H2BC	NM_003526.2	1.2904232	0.013236	0.04158
HIST1H2BC	JHU18563	0.476563	0.007548	0.034791
HIST1H4A	JHU17444	1.1938741	0.011145	0.03517
HIST3H2BB	NM_175055.2	-0.1280636	0.067056	0.007407
HK3	BC028129.1	-0.0117063	0.006773	0.035798
HKDC1_frag	JHU00138	-0.500083	0.005455	0.028872
HLA-A	NM_002116.5	-1.1507664	0.009104	0.044367
HLA-DMA	JHU04936	2.5550983	0.013178	0.021797
HLA-DPB1	BC015000.1	-0.9358913	0.019775	0.011274
HLA-DRA	NM_019111.3	0.8402813	0.031137	0.01271
HLA-DRB5	NM_002125.3	-0.3006934	0.003671	0.010735
HMGCR	BC033692.1	1.026113	0.006017	0.039701
HMGN3	NM_138730.1	0.5259617	0.005908	0.020577
HMGN4	HMGN4	-0.1321798	0.010582	0.034744
HNRNPC	BC089438.1	0.6322762	0.005236	0.04001
HNRNPDL	BC071944	1.3668325	0.035878	0.018566
HNRNPU	NM_004501.3	-0.217591	0.012525	0.000992
Hoxc12	BC120847	1.1089578	0.012095	0.030303
HOXC5	NM_018953.2	0.2830272	0.008925	0.023118
HPCAL4	JHU17329	1.1168261	0.002554	0.000593

HRAS	BC006499.2	0.2953324	0.013265	0.046877
HRH1	NM_000861.2	0.4776084	0.017848	0.04504
HSD17B12	BC012536.1	-3.2910614	0.007409	0.0251
HSD3B7	NM_025193.2	-0.3522949	0.001934	0.00297
HSF1	NM_005526.1	0.1089721	0.004718	0.029989
HSFY1	JHU14555	-0.0061315	0.009957	0.014734
HSPA12A	JHU18091	0.7781484	0.010836	0.033397
HSPA5	NM_005347.2	0.9873691	0.003937	0.015275
HSPB1	NM_001540.2	-0.290492	0.011141	0.005811
HSPB8	NM_014365.2	1.8882502	0.016988	0.043781
HVCN1	BC007277.1	1.6656296	0.011279	0.001553
HYAL3	BC005896.1	0.8306711	0.007325	0.009575
HYKK	ENST00000360519	-1.7972219	0.013594	0.013746
ICAM4	JHU08865	-0.3117769	0.009295	0.026074
ID4	NM_001546.2	0.6224354	0.006632	0.027163
IDNK	NM_001001551.1	0.1375821	0.011177	0.033848
IFI6	NM_022872.2	0.6218407	0.007979	0.012797
IFIT2	NM_001547.3	0.6551913	0.003053	0.018658
IFNE	NM_176891.3	-0.4754163	0.000917	0.008104
IFNL1	IL29	1.030909	0.011988	0.0056
IFT22	BC004522.2	1.8432766	0.015132	0.026175
IFT81	NM_014055.2	-0.843654	0.016688	0.034954
IGFALS	BC025681.1	-0.7647081	0.033624	0.048477
IGFL2	BC130490.1	0.2537058	0.004818	0.022705
IGHA1	BC073771.1	-0.04856	0.00541	0.022604
IGHG1	BC051328.1	1.2548785	0.001405	0.001619
IGHG1	BC069020.1	1.1753434	0.006746	0.025119
IGHG1	BC062336.1	0.4602964	0.015196	0.010766
IGHG1	BC018747.1	-0.0965816	0.009561	0.029619
IGHG1	BC089417.1	-0.2651405	0.008834	0.0477

IGHG1	JHU08300	-1.4702353	0.01537	0.044435
IGKC	BC070335.1	0.8919093	0.005938	0.003256
IGKC	BC073793.1	0.1960318	0.008513	0.018916
IGL	BC032452.1	1.6048049	0.010054	0.023145
IHH	NM_002181.3	1.1151538	0.003569	0.007294
IK	BC013005.2	-1.5808475	0.005123	0.006004
IL10RA	NM_001558.2	0.9304979	0.008159	0.000633
IL12A	NM_000882.2	0.4989032	0.007874	0.041818
IL15	BC100962.1	0.1457044	0.005762	0.047777
IL15RA	NM_002189.3	0.0974892	0.002908	0.021568
IL17F	BC070124.1	0.3451078	0.010208	0.028736
IL18BP	ENST00000337131	0.0629113	0.001326	0.004575
IL1R1	BC067508.1	0.1448248	0.013392	0.002831
IL1RN	NM_173842.1	0.9491811	0.021847	0.022247
IL2	BC066256.1	-0.1044421	0.05351	0.024216
IL20RB	NM_144717.2	-0.0815761	0.034386	0.038848
IL22RA2	NM_181309.1	-0.6320429	0.048787	0.037543
IL2RG	NM_000206.1	-0.1721536	0.007766	0.020767
IL4	BC067514.1	-0.257585	0.040461	0.004613
IL7R	BC067539.1	0.1062573	0.003648	0.033767
ILVBL	BC011761.2	0.6046079	0.005827	0.00334
IMMP1L	NM_144981.1	-1.9897925	0.020939	0.005753
IMPDH1	P20839	-1.1318321	0.019431	0.020029
ING3	NM_198267.1	0.8377625	0.010377	0.044446
ING3	NM_019071.2	0.3121047	0.004872	0.049813
ING3	ENST00000315883	0.2628384	0.012651	0.019148
INPP1	NM_002194.2	-0.5757658	0.001216	0.016702
INPP5D	NM_001017915.1	0.3975233	0.010105	0.006284
INPP5F	NM_198331.1	-0.0327147	0.002561	0.012429
INSL4	NM_002195.1	-0.8868765	0.013859	0.037076

INSRR	NM_014215	2.0018121	0.021054	0.038504
INTU	ENST00000296461	-1.849003	0.037753	0.034824
IP6K2	BC001864.1	0.0840003	0.003939	0.027327
IQGAP2	NM_006633	-0.8261981	0.010271	0.007861
IRAK4	NM_016123.1	0.5527689	0.006892	0.032552
IREB2	BC017880.1	0.2193685	0.001378	0.007473
IRF5	NM_032643.3	-0.0670666	0.0282	0.021334
IRX4	BC110912	0.460465	0.016732	0.023692
ISLR	BC022478	0.2649686	0.007747	0.020999
ITGA2	NM_002203	0.9945417	0.022019	0.012022
ITGAE	ITGAE	-0.5172247	0.002935	0.044767
ITGB1BP2	NM_012278.1	1.6926058	0.009538	0.049025
ITIH6	NM_198510	0.2834534	0.007168	0.03639
ITLN2	ITLN2	-0.5131919	0.008656	5.62E-06
ITM2C	NM_001012514.1	-1.0176184	0.025307	0.02484
ITM2C	BC002424.1	-1.5840279	0.015357	0.0259
JADE2	BC004292.1	-1.5686405	0.02829	0.02723
JADE3	NM_014735.2	-0.9008569	0.041545	0.004227
JAG1	NM_000214.1	0.7162505	0.007006	0.039579
JAGN1	BC032101.2	-0.8324508	0.018904	0.020745
JAK3	NM_000215.3	0.6071032	0.029984	0.028551
JAM3	BC012147.1	1.3705701	0.006201	0.022016
JHU10735	JHU10735	0.442355	0.020044	0.016133
JHU12892	JHU12892	0.7638772	0.063672	0.011614
JHU19600	JHU19600	-1.3998342	0.023122	0.007757
JMJD1B	BC001202.1	1.4803252	0.00844	0.010121
JUN	BC068522.1	-0.5510757	0.057911	0.007659
JUNB	JHU13061	-1.5364615	0.036942	0.027394
KANSL1	BC006271.1	-0.6300158	0.009362	0.036169
KANSL1L	NM_152519.2	1.1472639	0.024152	0.003128

KANSL2	BC013900.2	0.0154163	0.006944	0.028546
KCNAB3	NM_004732.2	-0.8354252	0.030691	0.03384
KCNE2	NM_172201.1	-0.3413448	0.012663	0.000645
KCNE4	NM_080671.1	1.0082588	0.008981	0.033005
KCNG1	NM_172318.1	0.4151317	0.01058	0.014266
KCNIP1	NM_014592.2	-0.9978904	0.05308	3.25E-06
KCNIP2	NM_173191.2	1.6788405	0.008407	0.044892
KCNJ10	NM_002241.3	-1.04711	0.004315	0.003521
KCNK12	NM_022055.1	0.1908663	0.008987	0.047309
KCNK18	NM_181840	-0.589809	0.049423	0.004253
KCNK2	NM_014217.2	-0.2391819	0.01083	0.037967
KCNMB3	NM_171829.1	1.2625547	0.010133	0.013034
KCNN3	NM_170782.1	1.606639	0.005199	0.022545
KCNQ3	NM_004519	1.5297936	0.020932	0.029606
KCNT2	BC103948.1	-0.0531498	0.038428	0.012476
KCTD18	BC059366.1	2.8081472	0.006625	0.010044
KCTD7	NM_153033.1	1.4316187	0.011158	0.007913
KDM2A	BC047486.1	1.3281665	0.010761	0.012933
KDM4B	NM_015015.1	0.7423518	0.003094	0.019619
KDM4D	NM_018039.2	1.2917265	0.016527	0.02377
KIAA0319L	BC014530.1	-1.3048581	0.052906	0.04562
KIAA0408	NM_014702.3	0.5377404	0.011919	0.002111
KIAA0664L3	BC000552.1	0.7814495	0.00542	0.035549
KIAA1024	NM_015206	1.0505257	0.001437	0.003662
KIAA1467	NM_020853.1	0.781972	0.017818	0.0427
KIAA1522	JHU18098	0.5879112	0.006519	0.01686
KIAA1715	NM_030650.1	1.433543	0.008578	0.014347
KIF16B	JHU14627	-0.5791177	0.022412	0.004852
KIF2C	BC008764.2	1.1402955	0.005926	0.022674
KIFC3	ENST00000268514	1.34013	0.007111	0.032845

KIR2DL2	NM_014219	-0.7129814	0.010531	0.037774
KIRREL2	BC007312.1	-2.0533873	0.023066	0.022337
KIRREL3	NM_032531.2	1.4349617	0.00332	0.031078
KITLG	NM_003994.4	0.5966623	0.002778	0.00314
KIZ	JHU16162	-1.5157176	0.0237	0.047748
KJ900282.1	JHU04799	0.1275759	0.004148	0.016407
KJ900895.1_frag	JHU03990	1.3086775	0.005647	0.034352
KJ900954.1_frag	JHU11380	0.2588826	0.01873	0.012044
KJ900957.1_frag	JHU06766	0.7228464	0.023791	0.037225
KJ901014.1	JHU16367	-0.0256147	0.00939	0.025333
KJ901136.1_frag	JHU07736	0.093768	0.000627	0.004125
KJ901169.1_frag	JHU15524	0.6322563	0.033866	0.040297
KJ901204.1_frag	JHU11911	0.0552601	0.016669	0.006727
KJ901215.1	JHU08036	1.114192	0.002527	0.039418
KJ901253.1_frag	JHU09638	1.7658877	0.014091	0.016169
KJ901255.1_frag	JHU10224	0.7858898	0.006501	0.04114
KJ901597.1	JHU07824	0.9453251	0.003869	0.023539
KJ901878.1	JHU06499	1.2624277	0.014831	0.034648
KJ901923.1_frag	JHU03190	0.174267	0.009747	0.001329
KJ901963.1_frag	JHU12050	-0.0851391	0.00771	0.015673
KJ902159.1	JHU01899	0.4825288	0.070986	0.02197
KJ902228.1	JHU03634	-1.8197853	0.015556	0.03549
KJ902277.1_frag	JHU09143	-1.4206279	0.019759	0.015523
KJ902398.1	JHU09422	-1.5545514	0.044676	0.027598
KJ902572.1	JHU09130	-2.40369	0.02806	0.034724
KJ903107.1	JHU08223	0.2016394	0.011819	0.04815
KJ903117.1_frag	JHU03928	0.9030786	0.062388	0.024837
KJ903230.1	JHU04229	0.1561788	0.008935	0.043586
KJ903246.1	JHU01861	-1.6657821	0.033323	0.023868
KJ903335.1_frag	JHU00385	-0.1389051	0.024641	0.020638

KJ903429.1	JHU12263	1.4215559	0.031888	0.044571
KJ903453.1_frag	JHU12739	-0.1441631	0.002694	0.02076
KJ903543.1_frag	JHU04029	0.6328152	0.010214	0.048294
KJ903622.1	JHU07968	1.18592	0.024058	0.048832
KJ903648.1	JHU07457	0.420596	0.007271	0.046906
KJ903660.1	JHU15199	0.7796705	0.01577	0.02552
KJ903907.1	JHU08243	1.6907135	0.009209	0.03088
KJ904326.1_frag	JHU17071	0.1050762	0.00613	0.048084
KJ904328.1_frag	JHU16115	-1.6136516	0.020136	0.000399
KJ904383.1_frag	JHU02305	1.305162	0.022566	0.037065
KJ905803.1	JHU12583	0.1619781	0.006387	0.032387
KJ905804.1	JHU03978	1.7153728	0.005748	0.017165
KJ905887.1_frag	JHU07270	-0.5358956	0.03154	0.02366
KJ906200.1	JHU08382	0.0810248	0.009764	0.024
KLF15	NM_014079.2	-0.5475678	0.025051	0.009115
KLF6	BC004301.1	0.3096943	0.005154	0.045012
KLF7	BC012919.1	2.8929593	0.018632	0.020844
KLHDC2	NM_014315.2	0.9603901	0.004791	0.012204
KLHDC7B	NM_138433.2	1.7007437	0.009047	0.047857
KLHDC8A	BC036076.1	0.548265	0.010278	0.041511
KLHL1	NM_020866.1	-0.597998	0.027642	0.023976
KLHL13	NM_033495.2	0.6769648	0.00551	0.001062
KLHL2	NM_007246.2	1.8113065	0.005196	0.034951
KLHL26	BC026319	0.2602163	0.006419	0.012985
KLHL41	BC006534	0.0315304	0.011728	0.045724
KLK1	BC005313.1	-0.9148154	0.007487	0.042506
KLK11	NM_006853.2	1.3928694	0.124944	0.038626
KLK11	NM_144947.1	0.5817485	0.010004	0.019421
KRAS	NM_033360.2	1.4308139	0.020846	0.002962
KREMEN2	NM_024507.2	-0.3442954	0.024174	0.01732

KRI1	NM_023008	0.5042335	0.003042	0.017792
KRT17	NM_000422.1	0.2692304	0.008036	0.003869
KRT3	NM_057088	0.7285163	0.001294	0.008879
KRT40	KRT40	0.9212325	0.004945	0.02696
KRT6A	BC014152.2	-0.5321686	0.044506	0.004333
KRT6A	BC069269.1	-0.8136654	0.017779	0.027613
KRT9	NM_000226	0.7290931	0.000758	0.038916
KRTAP10-2	NM_198693	0.6361443	0.003738	0.015806
KRTAP10-6	NM_198688	0.1743219	0.042734	0.037157
KRTAP10-7	NM_198689.1	0.0735154	0.003204	0.016011
KRTAP19-6	NM_181612	0.7242799	0.024619	0.021962
KRTAP3-3	NM_033185.2	2.8861657	0.003325	0.012447
KRTAP5-2	NM_001004325	0.4787013	0.028153	0.031968
KRTAP5-4	NM_001012709	-1.1006045	0.031396	0.007414
KSR1	KSR1	0.4217324	0.02452	0.010261
L3MBTL3	BC060845.1	-1.0370405	0.063836	0.031485
LACTB2	NM_016027.1	0.7556521	0.005878	0.045
LAMA5	BC003355.1	0.8862871	0.017169	0.02113
LAMP2	JHU04749	0.0634339	0.018289	0.038159
LARP1	BC033856.1	2.4406177	0.023083	0.031776
LAS1L	NM_031206.2	-0.1165705	0.003658	0.039088
LAS1L	BC019302.1	-0.2579837	0.007963	0.043013
LBHD1	JHU09320	0.4186949	0.004977	0.02749
LCE1C	JHU18783	2.6072046	0.002124	0.004256
LCE1D	NM_178352	0.4898221	0.021585	0.049309
LCE5A	NM_178438	0.4022641	0.015632	0.020115
LCK	BC013200.1	1.4161135	0.06178	0.044318
LCN10	NM_001001712.2	-0.3144901	0.024251	0.019348
LCORL	NM_153686.4	1.2744508	0.007182	0.032959
LDB1	NM_003893.3	1.0725468	0.010855	0.031065

LDHA	NM_005566.1	-0.0211911	0.019605	0.037179
LECT1	NM_007015.2	1.048671	0.026795	0.030714
LEP	BC069452.1	-0.5310342	0.032343	0.019968
LETMD1	JHU10123	1.481176	0.009105	0.038906
LFNG	BC014851.1	-0.6871856	0.007731	0.047087
LGALS13	BC066304.1	1.597405	0.018226	0.030573
LGI4	NM_139284.2	1.9915815	0.08307	0.022088
LGR6	NM_001017404.1	0.3856375	0.003011	0.003722
LHFPL4	NM_198560.2	0.6958419	0.007329	0.036483
Lhx1	Lhx1	0.0359495	0.006957	0.026614
LHX5	BC109230	0.3268409	0.025446	0.048942
LIFR	NM_002310.5	0.0371633	0.00508	0.033984
LILRA5	NM_021250.2	0.210895	0.111094	0.046284
LIMA1	NM_016357.3	1.1099353	0.004954	0.037399
LIMK1	LIMK1	0.4350201	0.008339	0.030197
LINC00313	BC107109.2	0.6997062	0.052096	0.032057
LINC00846	JHU01645	-0.8465538	0.064052	0.045256
LINC01587	JHU08457	0.4841596	0.038947	0.006682
LIPK	NM_001080518.1	1.1982607	0.011242	0.03617
LK937951.1_frag	JHU03515	1.2587994	0.008483	0.02033
LLPH	JHU07686	-0.8615816	0.019963	0.003525
LMNB1	BC012295.1	1.3181008	0.030103	0.01824
LN608403.1_frag	JHU11898	-0.1035056	0.055258	0.027609
LNX1	BC022983.1	0.6784808	0.006322	0.04754
LNX1	BC034737.1	0.117832	0.001673	0.001603
LOC100996763	JHU16014	0.4425643	0.003475	0.02164
LOC387942	XM_373573.2	1.1917508	0.00631	0.028917
LOC407835	BC067760.1	1.4052542	0.010534	0.039893
LPAR2	NM_004720.4	-1.005523	0.059644	0.031198
LPAR4	BC095538.1	0.5945863	0.005846	0.001934

LPIN1	NM_145693.1	0.9208607	0.001663	0.004213
LRBA	BC064834.1	-0.0001426	0.013575	0.048465
LRCH1	LRCH1	1.3500889	0.008093	0.003073
LRFN4	NM_024036.3	0.8104299	0.00416	0.012959
LRIG1	BC071561.1	0.9251268	0.00757	0.048286
LRP1	BC045107.1	1.6781714	0.055051	0.044295
LRRC23	BC029858.1	1.4790434	0.013583	0.005304
LRRC42	NM_052940.3	0.0937784	0.005422	0.01015
LRRC4C	NM_020929.1	0.4568566	0.003659	0.030053
LRRC56	NM_198075.1	-0.9939263	0.064249	0.041677
LRRN2	BC034047.1	1.6465075	0.0144	0.024068
LSM12	NM_152344.1	1.0794697	0.000967	0.007992
LSM2	BC009192	0.2193553	0.01278	0.042231
LSM3	NM_014463.1	0.4521458	0.01067	0.043314
LSMEM1	NM_182597.1	0.9377068	0.015161	0.007761
LTBP3	NM_021070.4	-1.4500503	0.029651	0.034591
LUC7L	JHU05134	-0.128353	0.022025	0.027715
LUC7L2	BC042625.1	0.9312489	0.015787	0.013365
LURAP1	NM_001013615.1	0.3919059	0.004622	0.024272
LUZP1	BC051733.1	1.2873858	0.010149	0.04192
LUZP2	NM_001009909.2	1.3786072	0.005667	0.016482
LY6G5C	NM_001002849.1	-1.302969	0.004775	0.029209
LY6G6F	NM_001003693.1	1.0937102	0.005306	0.032513
LY96	NM_015364.2	0.7092683	0.024853	0.018455
LYAR	JHU13464	-1.2657862	0.0151	0.010692
LYG2	NM_175735.3	-0.3419871	0.025817	0.003978
LYPD2	NM_205545.1	0.3869178	0.016274	0.022987
LYRM7	JHU12402	1.5614372	0.054489	0.049249
MAF	NM_005360.2	-0.1512862	0.003244	0.033665
MAFG	NM_002359.2	-1.3723277	0.00905	0.004608

MAGEA12	NM_005367.4	-0.3477995	0.002849	0.034852
MAGEA2	BC013098	0.2873152	0.006956	0.02052
MAGEA6	BC067731.1	0.0888399	0.001754	0.016988
MAGEA9	NM_005365.4	-0.4178822	0.002347	0.033046
MAGEB10	BC101307.2	1.1226598	0.023638	0.000363
MAGEB4	BC032852.2	0.457679	0.044512	0.043436
MAGEE1	BC050588.1	-1.0376393	0.001709	0.009276
MAN1B1	BC006079.1	-0.4038446	0.007696	0.014231
MANSC1	BC032998.2	1.0911708	0.005319	0.01513
MAP1LC3B	BC067797.1	0.434142	0.005865	0.029393
MAP2K3	NM_145109.1	0.9343987	0.005445	0.030765
MAP2K7	BC038295.2	1.2373539	0.008667	0.044042
MAP3K10	NM_002446.3	0.1643171	0.009605	0.045397
MAP3K13	NM_004721.3	1.5455588	0.04557	0.024285
MAP3K19	ENST00000315513	-0.9862932	0.008397	0.009143
MAP3K2	NM_006609.4	1.1825655	0.009012	0.04546
MAP3K5	NM_005923.3	0.3864625	0.019132	0.014468
MAP3K7	NM_145331.1	0.8699297	0.019775	0.011656
MAP4K2	BC047865.1	-0.6953684	0.024597	0.042404
MAPK12	NM_002969.3	1.2867843	0.006314	0.031365
MAPK4	MAPK4	1.3984353	0.013983	0.034863
MARCH2	NM_001005416.1	1.6080444	0.012628	0.041932
MARCH6	NM_005885.2	0.0126375	0.002503	0.034542
MARK4	MARK4	-0.014381	0.007117	0.02821
MARVELD2	JHU05049	1.4329956	0.026729	0.019996
MASP1	NM_001031849.1	-1.2679401	0.02244	0.002872
MAZ	BC041629.1	0.7741048	0.002064	0.015608
MBD3L1	NM_145208.1	0.2729878	0.001649	0.00029
MC2R	NM_000529.2	-0.4571985	0.005758	0.014298
MCAM	JHU07062	-0.0260913	0.010573	0.040101

MCM8	NM_032485.4	1.33869	0.012846	0.008289
MCPH1	BC030702.1	2.0403132	0.017524	0.049096
MDFIC	NM_199072.4	0.8867406	0.00515	0.004568
MDM2	NM_006879.2	0.4251895	0.011396	0.004318
MECR	JHU00333	-2.3576065	0.015499	0.035775
MED15	PCQAP	-0.4394905	0.01917	0.025795
MED17_frag	JHU04683	-0.9624122	0.001388	0.018229
MED28	NM_025205.3	-0.0371046	0.001629	0.004444
MED30	NM_080651.1	1.4658378	0.003472	0.021721
MED8	NM_001001654.1	2.1591792	0.010123	0.036211
MEF2B	JHU13645	0.9553204	0.006675	0.034304
MEGF6	JHU19118	1.3078464	0.012514	0.039182
MELK	NM_014791.3	-0.5279319	0.011123	0.022181
MEPCE	BC000556.2	1.0124757	0.009331	0.00193
MEPE	NM_020203.3	-0.123153	0.008882	0.015958
METTL14	NM_020961.2	1.0419206	0.017228	0.046176
METTL25	NM_032230.1	-0.8230078	0.018354	0.003954
MFAP4	BC062415.1	2.0371836	0.020382	0.030383
MFI2	NM_033316.2	1.7942875	0.01175	0.01531
MFSD3	NM_138431.1	-1.0924199	0.01652	0.035678
MIA2	NM_054024.3	0.4538769	0.008971	0.003062
MIB2	JHU17358	0.7045216	0.008448	0.015718
MICU1	BC004190.2	-1.4603099	0.009024	0.010638
MICU2	NM_152726.1	0.7151474	0.010436	0.033936
MIF	NM_002415.1	-0.2927439	0.020344	0.004871
MIF4GD	NM_020679.2	-0.1364326	0.02207	0.012717
MIOX	CR456478	0.2092629	0.010941	0.041668
MKNK2	NM_017572.2	0.6195862	0.011161	0.049037
MKNK2	NM_199054.1	0.1609406	0.060017	0.00771
MKRN2	JHU01198	1.2357511	0.0051	0.012397

MLEC	JHU29112	1.1759907	0.029381	0.026497
MLKL	BC028141.1	1.7592094	0.009791	0.005274
MLKL	JHU08209	1.1350433	0.005606	0.01126
MLKL	NM_152649.1	0.4634368	0.003958	0.037396
MLLT6	BC064612.1	0.5866043	0.006127	0.049103
MLXIP	MLXIP	0.035434	0.018016	0.035111
MLXIPL	MLXIPL	0.6918233	0.011113	0.040762
MMADHC	NM_015702.1	0.3642931	0.010767	0.01505
MMGT1	NM_173470.1	0.7456258	0.008333	0.044397
MMP1	NM_002421.2	0.1681114	0.017243	0.006895
MMP28	BC002631.2	-0.0236809	0.014466	0.031555
MMP7	BC003635.2	-1.0273689	0.031782	0.038508
MMP9	BC006093.1	-0.160429	0.00509	0.020285
MOCOS	BC012079.1	-0.5877819	0.033792	0.040699
MORF4L1	BC067826.1	-1.1035084	0.02348	0.013831
MPHOSPH9	NM_022782.2	1.4320467	0.01467	0.0371
MPLKIP	NM_138701.1	-1.5707054	0.075615	0.033957
MPO	MPO	0.5698616	0.008476	0.045966
MPP5	BC095485.1	1.1354343	0.008435	0.045553
MPP6	BC023638	0.4154478	0.008521	0.030093
MPPE1	BC002877.2	-0.2983613	0.005504	0.009117
MPPED1	NM_001044370.1	0.016363	0.008345	0.013255
MR1	BC012485.1	-1.2690038	0.009194	0.026863
MRAS	BC047690.1	0.0687591	0.014524	0.019911
MREG	BC082990.1	0.7680758	0.016231	0.004291
MRPL19	ENST00000233713	0.2370145	0.015729	0.003243
MRPL21	NM_181515.1	1.186074	0.024671	0.022951
MRPL34	NM_023937.2	1.383775	0.003133	0.001151
MRPL43	NM_176793.1	1.2954891	0.012617	0.033351
MRPL43	NM_176792.1	-0.0747232	0.028075	0.04014

MRPL46	NM_022163.2	1.1103158	0.002395	0.011342
MRPL47	NM_177988.1	-1.135959	0.002084	0.010985
MRPL55	NM_181462.1	0.3523926	0.008441	0.030214
MRPS34	NM_023936.1	-2.1001876	0.061964	0.013331
MS4A3	NM_006138.4	0.1853323	0.008145	0.009596
MSL3	NM_006800.2	-1.2430762	0.004272	0.001035
MSN	BC017293.2	0.9081057	0.005759	0.041387
MSRB2	NM_012228.3	1.2226124	0.009187	0.004757
MST1R	NM_002447.2	0.9461902	0.013689	0.031137
MSTN	BC074757.2	0.18521	0.007186	0.035621
MT1DP	NM_175620.1	-0.1868391	0.024047	0.03107
MT1F	NM_005949.1	1.1547544	0.057012	0.042201
MT1M	NM_176870.1	0.3653386	0.006196	0.031472
MT2A_frag	JHU12505	-0.1273565	0.029565	0.04543
MTAP	BC026106.1	-1.2979273	0.085239	0.048947
MTERF2	JHU15437	0.4436601	0.004235	0.021864
MTG1	BC035721.1	0.1504979	0.008805	0.029289
MTHFD2	BC001548.1	0.3140045	0.019556	0.048277
MTM1	BC030779.1	0.7978923	0.121455	0.006963
MTNR1A	MTNR1A	0.2506425	0.007405	0.014557
MTPN	NM_145808.1	0.8336632	0.008444	0.041521
MTX2	NM_006554.3	-0.3118211	0.040617	0.012772
MUC15	BC020912.1	0.798504	0.019044	0.020503
MUL1	BC014010.1	0.1207321	0.006078	0.034255
MVD	NM_002461.1	-1.1365094	0.006127	0.004194
MXI1	NM_005962.4	1.4687693	0.036757	0.038463
MYB	NM_005375.2	1.1488902	0.009527	0.030676
MYBPC1	AL834249	0.671033	0.023	0.030867
MYDGF	JHU08267	-0.2432546	0.001841	0.036324
MYL6	NM_079423.2	1.7017587	0.025034	0.027383

MYL7	NM_021223.2	-0.1011484	0.009902	0.028854
MYO5A	NM_000259	0.1732381	0.01318	0.003167
MYO9A	BC060886.1	-0.1444672	0.004687	0.020754
MYOD1	NM_002478.4	2.0389673	0.015528	0.038427
N4BP2L2	NM_033111.2	-0.2179856	0.002615	0.002171
NAA11	NM_032693	1.3760216	0.003977	0.006263
NAA60	NM_024845.1	1.8037027	0.008626	0.040805
NACC2	NM_144653.3	1.0210524	0.021785	0.030202
NADK	BC001709.1	-0.1950433	0.02158	0.014635
NAGLU	BC053991.1	-0.2477009	0.004267	0.000312
NAMPT	JHU11960	0.8547857	0.014952	0.001124
NANP	NM_152667.1	0.9106519	0.011657	0.003949
NAP1L5	NM_153757.1	-0.3446152	0.011387	0.012182
NAPG	BC001889.1	1.7126451	0.012678	0.047575
NAPRT	JHU15343	0.771554	0.009696	0.039246
NARF	NM_031968.2	-0.4914792	0.00161	0.022528
NAT6	Q93015	0.8148346	0.036608	0.01037
NAT8L	NM_178557.2	-0.8198476	0.01439	0.045743
NCAPG2	BC020560.1	1.1555476	0.009989	0.017487
NCAPH	JHU05238	1.1866824	0.005831	0.008445
NCAPH	BC024211.2	1.147761	0.038864	0.009771
NCF1	NM_000265.4	1.7146242	0.041396	0.023367
NCSTN	BC047621.1	0.9026267	0.004544	0.037924
NDFIP2	BC021988.1	-0.0551886	0.007544	0.046559
NDNF	ENST00000309554	-0.8005104	0.015277	0.008432
NDUFA13	BC009189.2	-0.1491762	0.021834	0.025176
NDUFA6	BC002772.1	1.6128522	0.007077	0.022009
NDUFAF7	NM_144736.3	1.3247952	0.010025	0.000229
NDUFB3	NM_002491.1	0.6928234	0.005469	0.032251
NDUFS4	BC005270.1	1.3859284	0.008297	0.035965

NECAB1	NM_022351.2	0.8059811	0.007957	0.033673
NEIL1	BC010876.1	-0.8194538	0.008653	0.015864
NEIL2	NM_145043.1	-0.9997997	0.016908	0.002765
NELFCD	JHU01911	0.8167018	0.019852	0.014033
NELL1	BC096100.1	0.6185212	0.016669	0.047265
NENF	JHU08793	-1.6662278	0.064266	0.022683
NEUROD2	BC022481.1	-0.7079509	0.011374	0.005948
NFAM1	BC038241.1	0.2246618	0.005152	0.017434
NFATC4	NM_004554.3	0.170025	0.01839	0.016808
NFS1	ENST00000306750	1.216502	0.011217	0.034251
NFU1	NM_001002755.1	0.5594812	0.006548	0.020841
NG_021302.1_frag	JHU04117	0.2083298	0.007576	0.029571
NG_042316.1_frag	JHU04608	-1.8647891	0.004824	0.033159
NGB	BC032509	0.4577049	0.012919	0.010978
NGLY1	BC017220.2	0.9274591	0.05618	0.014779
NHLRC3	NM_001017370.1	1.2559404	0.013524	0.045191
NIF3L1	BC007654.2	0.3069482	0.012658	0.033438
NIPA1	BC025678.1	1.2855103	0.01196	0.01499
NIPA2	BC011775.2	-1.8882841	0.013439	0.019177
NKX6-1	JHU17838	0.0700564	0.015003	0.021986
NLRC4	BC031555	1.249715	0.011151	0.001811
NLRP11	NM_145007.1	-2.7501407	0.017246	0.021972
NLRP14	NM_176822	-0.8698057	0.007503	0.020735
NLRP2	JHU07643	1.1125382	0.016821	0.02794
NM_001018042.3_frag	JHU19855	0.4401012	0.00704	0.001567
NM_001291029.1_frag	JHU19838	0.9333713	0.007522	0.000603
NM_007674.3_frag	JHU19618	-0.4017016	0.008301	0.01539
NM_007892.2_frag	JHU19626	0.7264817	0.012566	0.04214
NM_008592.2_frag	JHU19576	0.1481482	0.00866	0.019869
NM_009234.6_frag	JHU19601	1.5090257	0.016344	0.035993

NM_010451.2_frag	JHU19648	1.0238131	0.009787	0.019414
NM_017877.3_frag	JHU07689	1.3101635	0.0017	0.010787
NM_024104.3_frag	JHU02702	1.5226977	0.018498	0.047632
NME4	NM_005009.2	0.6111788	0.054045	0.049728
NMNAT2	NM_015039.2	-1.7140894	0.032585	0.008329
NMU	JHU03608	-0.1485233	0.031141	0.038736
NOA1	JHU18161	0.0513156	0.004807	0.04069
NOBOX	NM_001080413	0.5356955	0.005828	0.044267
NOP16	BC040106.1	-0.2026337	0.024386	0.047728
NOX1	NM_013955.1	0.4114108	0.004507	0.035653
NOX3	NM_015718	-0.1944062	0.008796	0.004482
NPDC1	BC004217.2	-0.609775	0.002662	0.015584
NPHP4	BC050076.1	-0.7194242	0.008309	0.027972
NPM1	BC012566.1	0.6986131	0.00126	0.008344
NPPC	NM_024409.2	0.1848242	0.006226	0.017916
NPTXR	NM_014293	1.2482793	0.009783	0.001717
NPVF	NM_022150	2.4840368	0.008357	0.035411
NPY5R	NM_006174.2	0.4722913	0.008556	0.04776
NPY5R	JHU07645	0.0798189	0.009251	0.006469
NR_003581.2_frag	JHU15426	0.1382035	0.005073	0.044374
NR_026908.1_frag	JHU14436	0.8301074	0.0101	0.048825
NR_027074.1_frag	JHU10844	-1.3908245	0.008681	4.27E-05
NR_131935.1_frag	JHU03073	0.1893709	0.009456	0.033363
NR2C2	BC051670.1	0.8904538	0.015969	0.002803
NRGN	NM_006176.1	0.2777253	0.013343	0.02424
NRN1L	BC100863.2	-0.9803694	0.007976	0.03984
NSRP1	NM_032141.2	-0.0917613	0.017233	0.043537
NSUN4	BC014441.1	1.3677211	0.049924	0.040123
NTF4	NM_006179.3	0.5001044	0.005239	0.034975
NTS	NM_006183.3	-1.4781971	0.022314	0.011334

NTSR2	JHU08215	0.3774989	0.007803	0.005507
NUDT1	NM_002452.3	0.8465808	0.006024	0.042027
NUF2	NM_031423.3	0.183964	0.00745	0.002326
NUGGC	NM_001010906	0.01142	0.011738	0.040119
NUP133	NM_018230.2	0.9855582	0.072761	0.047978
NUP35	NM_138285.3	2.0673677	0.028812	0.039202
NUP62	NM_012346.3	1.5683901	0.003765	0.008435
NUP85	JHU03991	2.6341052	0.032974	0.023503
NUS1	NM_138459.3	1.0925472	0.02367	0.001787
NXN	BC104634	1.1747636	0.02738	0.034373
NXPH2	BC101461.1	1.7908515	0.002456	7.61E-05
NXPH3	NM_007225.1	1.1783975	0.017773	0.033105
NXPH4	BC036679.1	-0.3567838	0.022498	0.035255
NXT1	NM_013248.2	0.5067207	0.0285	0.025919
NYX	NYX	2.6645143	0.012997	0.036797
NYX	NM_022567.2	0.2614336	0.028109	0.038059
OAF	NM_178507.2	0.459939	0.003761	0.031075
OAZ1	OAZ1	2.1526068	0.008439	0.036926
ODAM	BC017796.1	3.7866545	0.012108	0.006232
ODC1	NM_002539.1	-0.7324386	0.001202	0.01643
ODF3	NM_053280.3	0.6670418	0.00869	0.046861
ODF4	NM_153007.3	-1.4061141	0.006468	0.000226
OFCC1	JHU16891	0.3203119	0.002226	0.002675
OGDHL	OGDHL	0.5055588	0.005262	0.012816
OGG1	NM_016829.1	1.4444808	0.003919	0.03293
OGG1	NM_016827.1	0.1680511	0.04963	0.027609
OGT	NM_181672.1	-3.5734067	0.012855	0.049176
OIP5	NM_007280.1	0.2455643	0.016432	0.013479
OLA1	BC013925.1	-2.2987833	0.01135	0.02218
OLFML2A	BC054001.1	1.6684337	0.010054	0.019471

OLIG2	BC036245.1	2.1426301	0.007729	0.024284
OLR1	NM_002543.2	0.0272504	0.021505	0.028546
OPA1	NM_015560.1	-0.5338026	0.00414	0.022888
OPA3	BC047316.1	0.7112147	0.013473	0.023558
OPCML	BC074742.2	0.4522189	0.028566	0.028141
OPHN1	NM_002547.1	1.5730618	0.008373	0.033041
OR10G6	JHU05701	-0.6316744	0.018126	0.039428
OR10T2	NM_001004475.1	0.1710675	0.000455	0.003382
OR11A1	JHU10140	1.5219717	0.002617	0.016053
OR11H4	NM_001004479.1	-1.4540814	0.099511	0.02222
OR1J1	NM_001004451.1	-1.0455991	0.007888	0.033988
OR1J4	NM_001004452	0.2621816	0.015221	0.013631
OR2C1	BC130328.1	-0.1072029	0.015915	0.019459
OR2L13	NM_175911.2	-0.8840822	0.019895	0.033775
OR2T1	NM_030904	1.6427593	0.007853	0.01637
OR2T10	NM_001004693.1	3.4245697	0.010209	0.039185
OR2T29	NM_001004694.1	-2.370795	0.012166	0.009549
OR4K1	NM_001004063.1	-0.621961	0.009527	0.030541
OR4N5	NM_001004724.1	-0.2105819	0.010284	0.037237
OR4X1	NM_001004726.1	-1.0455938	0.006382	0.020838
OR51B4	NM_033179.1	-0.5589897	0.011437	0.027714
OR51B6	NM_001004750	0.5598426	0.033442	0.013294
OR51F2	JHU07354	-0.7134377	0.008777	0.040594
OR51I1	NM_001005288.1	0.0321118	0.026292	0.04556
OR51M1	NM_001004756.1	0.2376083	0.011869	0.00992
OR52E6	NM_001005167.1	0.016898	0.042492	0.037718
OR5AC2	NM_054106	0.1824242	0.021859	0.026177
OR5AK3P	Q8NH89	0.8829885	0.01072	0.041655
OR5D16	NM_001005496.1	0.0131465	0.002889	0.022665
OR5F1	JHU09372	-1.6982007	0.016841	0.009316

OR5H14	NM_001005514.1	-2.0888329	0.024731	0.02038
OR5K1	NM_001004736	0.341947	0.052926	0.048759
OR6C4	NM_001005494.1	-2.784215	0.018443	0.029528
OR6Q1	ENST00000302622	-0.8995587	0.00514	0.029433
OR8A1	Q8NGG7	-2.2505239	0.007803	0.037118
ORMDL2	NM_014182.3	1.1620224	0.006615	0.045465
OSBPL1A	BC041563.1	1.0243677	0.003107	0.006475
OSGIN1	Q9UJX0	0.3906126	0.015686	0.044767
OSGIN2	NM_004337.1	-0.023214	0.029613	0.035393
OSR2	BC016936.1	0.7733875	0.013333	0.024523
OTOGL	BC101016.2	0.382418	0.07375	0.039424
OTP	NM_032109.2	1.4248796	0.008626	0.009427
P2RX7	BC011913.2	-1.5376415	0.013958	0.00363
P2RY1	NM_002563.2	0.6055353	0.002409	0.008922
P2RY2	BC012104.1	-1.145561	0.042709	0.002348
P2RY4	JHU15925	0.438155	0.018031	0.016533
P2RY4	BC096068.1	-0.1152271	0.016374	0.019398
P3H3	JHU17825	0.5302749	0.004689	0.005027
P3H4	JHU13200	0.4631594	0.002574	0.049265
P4HTM	NM_177938.2	0.1097205	0.008353	0.030472
PABPC1L2A	NM_001012977.1	0.8588537	0.005828	0.039327
PABPC3	NM_030979.2	1.0315718	0.009625	0.046052
PABPN1L	NM_001080487	1.0364604	0.004902	0.009949
PADI3	NM_016233.2	0.2340592	0.036559	0.033045
PADI6	NM_207421	-0.1411322	0.020802	0.04516
PAK7	BC024179	-0.734246	0.007512	0.033115
PANK2	ENST00000354720	-0.2880198	0.037345	0.007884
PANX1	BC016931.1	0.1029059	0.025016	0.020429
PAPLN	NM_173462.3	0.1048403	0.005485	0.047075
PAPOLB	BC036653	-0.733737	0.007717	0.032443

PAQR3	BC047510.1	0.3461439	0.039999	0.012822
PAQR9	NM_198504.2	1.4078023	0.044309	0.001525
PARD3B	NM_205863	-0.6934471	0.012664	0.003996
PARP11	NM_020367.3	-0.296588	0.009379	0.007364
PARS2	NM_152268.2	1.4923578	0.087717	0.035407
PARS2	JHU06781	-1.5822481	0.044712	0.0409
PARVA	NM_018222.2	-1.4776478	0.051532	0.008187
PASK	NM_015148.3	0.7965226	0.017248	0.020537
PATL2	ENST00000560780	-0.9796529	0.022876	0.037785
PATZ1	NM_032051.1	-0.5719157	0.024907	0.031781
PBLD	NM_022129.3	0.4594028	0.008379	0.000351
PCBP1	NM_006196.2	1.5507548	0.004034	0.011395
PCCA	BC000140.2	-0.8911013	0.026296	0.049131
PCDHA10	PCDHA10	1.1056654	0.009182	0.008857
PCDHA8	NM_031856.1	-0.7769478	0.005289	0.01676
PCDHGC3	BC004321.1	-0.4931793	0.004682	0.00886
PCNP	BC022001.2	1.0300117	0.044799	0.013305
PCP4L1	NM_001102566.1	1.01351	0.003896	0.025034
PCTP	JHU00829	0.7903674	0.0002	0.00272
PCYOX1	BC033815	1.6328168	0.027595	0.000289
PDCD2	NM_002598.2	-1.5958965	0.005121	0.013196
PDCD2L	NM_032346.1	1.825159	0.00543	0.015597
PDCL2	NM_152401.2	0.0012217	0.004684	0.005914
PDE1C	BC022525	0.1627271	0.004643	0.029784
PDE4D	BC008390.1	1.0882682	0.019598	0.045159
PDF	BC019912.1	-2.0657887	0.0245	0.044131
PDIA4	NM_004911.3	1.3620506	0.02391	0.032352
PDIA6	NM_005742.2	-1.1340801	0.007395	0.032035
PDLIM7	NM_005451.3	1.8131795	0.003555	0.010439
PDPK1	NM_031268.4	1.1366502	0.012841	0.018672

PDPN	NM_001006625.1	1.0084748	0.009985	0.040063
PDSS2	NM_020381.2	0.827996	0.010517	0.004058
PDZD4	PDZD4	0.7834331	0.012862	0.001593
PDZK1IP1	BC012303.1	-1.7311832	0.054003	0.001399
PER2	BC111453	0.6676205	0.037821	0.032428
PF4V1	NM_002620.2	-0.6256835	0.031292	0.000747
PFN2	BC018049.1	0.4499138	0.007465	0.024759
PGAM2	NM_000290.2	1.3687535	0.004757	0.010972
PGAM5	BC008196.1	1.8372523	0.005978	0.012342
PGBD3	JHU16221	0.1213484	0.000949	0.009331
PGBD4	NM_152595.3	-0.6500797	0.01598	0.009204
PGLS	NM_012088.2	1.2656325	0.006767	0.026074
PGM2	NM_018290.2	0.8074005	0.020105	0.0142
PGM2	BC010087.2	0.1401313	0.001146	0.022052
PGM2L1	BC059360.1	0.7662535	0.012239	0.03538
PGM3	NM_015599.1	0.8398708	0.002273	0.02231
PGRMC2	NM_006320.1	-0.8127125	0.003559	0.044114
PHACTR2	NM_014721.1	0.495342	0.011459	0.003481
PHACTR2	JHU14581	-0.4728037	0.012331	0.044018
PHACTR3	NM_183246.1	-0.575038	0.005455	0.017186
PHB	NM_002634.2	1.415152	0.027588	0.03074
PHOSPHO2	NM_001008489.2	-0.2858181	0.017564	0.037053
PHTF1	BC002447.2	0.6474056	0.025149	0.007636
PIFO	NM_181643.2	-0.4564113	0.00621	0.041993
PIGA	NM_020472.1	-0.1101366	0.002002	0.012266
PIGQ	NM_148920.1	-0.2920134	0.005002	0.02841
PIGR	NM_002644.3	1.7922998	0.009762	0.048255
PIP4K2A	NM_005028.3	0.0707502	0.012296	0.007994
PIPOX	NM_016518.2	1.524339	0.008514	0.042219
PIPSL	BC068549.1	0.7560477	0.017068	0.038129

PITRM1	BC095422.1	0.6117797	0.034718	0.006046
PLA1A	NM_015900.1	0.545417	0.006804	0.012662
PLA2G15	NM_012320.3	0.164757	0.007597	0.0332
PLA2G4C	BC017956.1	-0.2351588	0.033327	0.00264
PLA2G4D	BC034571.1	-0.8952205	0.009004	0.037714
PLA2G6	NM_001004426.1	-0.2473366	0.00669	0.006077
PLA2R1	NM_007366.3	0.14389	0.000517	0.012236
PLAC8L1	NM_001029869.1	0.6636215	0.01764	0.036188
PLAUR	NM_001005376.1	3.4839805	0.007013	0.02405
PLCXD1	NM_018390.2	0.5253593	0.003157	0.02968
PLEKHA4	BC064601.1	0.7632043	0.033706	0.027895
PLEKHA8	BC053990.1	0.1837678	0.102852	0.041187
PLEKHH3	JHU12690	-0.5097941	0.021978	0.038545
PLOD1	NM_000302.2	1.4606094	0.011788	0.039739
PLTP	BC005045.1	0.9185846	0.002541	0.016765
PMEPA1	NM_199169.1	-0.3677875	0.073074	0.020841
PMEPA1	NM_020182.3	-0.7049761	0.046737	0.033051
PNMAL1	NM_018215.2	-1.5843302	0.020652	0.002318
PNMT	JHU07360	0.2227788	0.011508	0.041131
PNN	NM_002687.2	-1.4906026	0.028298	0.04618
PNPLA7	NM_001098537	-0.0185695	0.01638	0.036661
PNPO	NM_018129.1	1.1028631	0.028023	0.001937
PNPO	JHU04578	-1.28671	0.023582	0.00189
PODN	BC030608.2	-1.3453396	0.003894	0.02734
PODXL2	NM_015720.1	-0.5184337	0.034813	0.008694
POLDIP3	BC095411	0.6794117	0.017702	0.049102
POLE2	POLE2	0.0707379	0.004573	0.008298
POLR2C	NM_032940.2	-1.2744841	0.021358	0.005261
POLR3H	BC088367	0.0901204	0.004786	0.041
POT1	NM_015450.2	0.87887	0.014533	0.048902

POTEA	NM_001005365	1.1091841	0.013735	0.049494
POTEE	NM_001004054.2	1.0996982	0.024106	0.006723
POU6F1	BC074765.2	-0.4029934	0.040781	0.007398
PPAP2A	JHU07361	-0.8492855	0.007413	0.023821
PPARG	ENST00000396998	-1.8629245	0.021206	0.042536
PPBP	NM_002704.2	-1.200686	0.046803	0.036056
PPHLN1	NM_201515.1	1.6999211	0.008399	0.004601
PPIL3	BC007693.2	-0.6184733	0.032771	0.038071
PPIP5K1	JHU07617	-0.8881648	0.013657	0.023446
PPP1R12C	NM_017607.3	0.1983067	0.059245	0.020342
PPP1R16A	NM_032902.5	0.7565632	0.008816	0.02312
PPP1R16B	NM_015568.2	1.2995946	0.014367	0.02256
PPP1R8	NM_138558.1	0.5049868	0.0077	0.021935
PPP2R2D	BC072402.1	1.056338	0.017648	0.026559
PPP2R5A	JHU05737	0.9186238	0.00996	0.013323
PPP2R5E	NM_006246.2	0.4841393	0.038713	0.011814
PPP3R2	NM_147180.2	-0.8551022	0.020678	0.041957
PPY	BC032225	1.6088961	0.008309	0.045079
PQLC2L	JHU16838	1.7060375	0.001451	0.011109
PRAMEF1	BC040902.1	-0.1410074	0.0299	0.010184
PRAMEF3	JHU17573	0.6926599	0.012306	0.018748
PRC1	NM_199414.1	1.3449824	0.060731	0.019784
PRDM1	BC103835.1	0.4163575	0.030899	0.039525
PRDM11	NM_020229	-0.5230023	0.004078	0.030262
PRELID2	NM_138492.4	-1.8871664	0.017403	0.018379
PRKACB	BC035058.1	-2.3815682	0.011302	0.027027
PRKAR1B	NM_002735.1	1.0087324	0.007018	0.014326
PRKCSH	BC013586.1	0.6399043	0.015238	0.036303
PRKD2	JHU10541	-1.6549934	0.005803	0.008466
PRMT7	NM_019023.1	-0.3700039	0.004491	0.01398

PROK1	BC025399.1	2.441248	0.022487	0.01227
PRPH2	BC074720.2	1.109848	0.010849	0.048885
PRR15L	NM_024320.2	1.5916941	0.009474	0.00238
PRRC2B	BC012289.1	-4.032027	0.024939	0.011344
PRRG2	BC026032.1	0.5098313	0.040605	0.002703
PRSS27	NM_031948.3	-0.5362642	0.042084	0.003208
PRSS54	BC057843.1	-0.160411	0.037976	0.021963
PRUNE2	BC022571.1	0.7777064	0.001075	0.008962
PSAPL1	NM_001085382.1	2.737062	0.015133	0.029865
PSAT1	BC016645.2	2.9823554	0.047996	0.022207
PSG7	NM_002783.2	0.5049013	0.021141	0.008471
PSMA1	NM_148976.1	0.3174211	0.003123	0.029371
PSMA3	BC029402.1	-0.0195962	0.013631	0.027168
PSMA4	BC056249.1	-0.7581437	0.021026	0.005944
PSMA5	NM_002790.2	-0.5647603	0.045584	0.031454
PSMA6	JHU02367	-0.1538121	0.010332	0.010484
PSMA6	NM_002791.1	-1.3583722	0.005778	0.001908
PSMA7	NM_002792.2	-0.4004299	0.004549	0.00351
PSMC6	NM_002806.2	-0.1466708	0.014236	0.038663
PSMD14	NM_005805.2	-1.3595366	0.045126	0.033179
PSMD6	NM_014814.1	-0.2580518	0.005504	0.00142
PSMD7	NM_002811.3	1.8571335	0.008133	0.039125
PSTPIP2	NM_024430.2	1.7797287	0.018207	0.022096
PTAFR	BC063000	-1.4238785	0.010471	0.047201
PTCHD4	NM_207499.1	0.0632586	0.002876	0.018334
PTF1A	JHU16679	0.3916863	0.002604	0.018151
PTGFR	NM_000959.3	1.1531406	0.017798	0.01197
PTP4A3	NM_007079.2	0.1769655	0.006737	0.026966
PTPN18	JHU07654	-0.08664	0.010521	0.033056
PTPN2	NM_080423.1	1.1353231	0.024166	0.012229

PTPN21	NM_007039	0.4057202	0.043692	0.017134
PTPN22	ENST00000460620	-0.8250559	0.009407	0.013712
PTRF	BC008849.1	0.4086195	0.006021	0.026765
PURA	BC036087	1.5013006	0.025097	0.012743
PVRL1	NM_203286.1	1.5221418	0.01265	0.026959
PVRL3	BC017572.1	1.6211105	0.005051	0.031226
PWP2	BC013309.2	1.3922365	0.014834	0.043316
PWWP2B	NM_138499.2	3.085253	0.033358	0.027868
PXYLP1	NM_001037172.1	0.8387331	0.002606	0.048847
PYHIN1	NM_198929.2	0.8636306	0.007461	0.021765
PYY2	Q9NRI6	1.2300673	0.001418	0.008491
Q8N6X1	ENST00000325042	2.1223592	0.009877	0.003172
QKI	NM_006775.1	0.0499811	0.044613	0.033286
QPRT	NM_014298.3	0.8776617	0.032966	0.047802
QPRT	BC005060.1	0.068638	0.022743	0.046691
QRFP	NM_198180.1	0.8319999	0.004766	0.014707
QRFP	BC101127	-0.6679312	0.009321	0.001445
QSOX1	JHU17238	0.7035378	0.023621	0.02116
RAB10	BC000896.1	2.4297994	0.028999	0.038767
RAB12	NM_001025300	0.2310987	0.018184	0.043448
RAB24	NM_001031677.2	0.6640762	0.01097	0.023767
RAB28	NM_001017979.1	0.0701008	0.009354	0.009994
RAB31	BC001148	0.6394646	0.007738	0.033952
RAB34	JHU00254	-0.3657997	0.023479	0.029965
RAB35	NM_006861.4	-0.5908385	0.056869	0.024565
RAB37	NM_175738.3	0.7860093	0.007752	0.042956
RAB3C	NM_138453.2	0.5910461	0.005629	0.00175
RAB40B	NM_006822.1	-1.920875	0.036825	0.01273
RABGGTB	NM_004582.2	-0.101283	0.003499	0.000967
RABL2A	CR456554	0.6146632	0.015404	0.04875

RABL4	BC000566.2	1.5905125	0.015377	0.005163
RAD51C	NM_058216.1	0.4218747	0.008307	0.031887
RAD51D	NM_133627.1	-0.401037	0.091529	0.025907
RAD54B	JHU13859	0.1883397	0.007139	0.005016
RALGDS	BC059362.1	0.0409314	0.029634	0.041514
RALY	BC103753.1	1.8967906	0.001735	0.004184
RAP1A	NM_001010935.1	-1.2541514	0.086798	0.010021
RAP2C	BC003403.1	0.0403092	0.004004	0.015927
RARRES1	NM_206963.1	-0.0009636	0.011844	0.049083
RASA4	RASA4	-0.2972881	0.003259	0.039561
RASD1	NM_016084.3	-0.8935715	0.004095	0.014217
RASGEF1A	BC022548.1	0.9745261	0.004998	0.012475
RASGRP2	NM_005825.2	0.7743165	0.010619	0.041373
RASL10B	NM_033315.2	-0.8646245	0.014387	0.012063
RASL11B	NM_023940.2	0.2212611	0.04682	0.030632
RASSF1	ENST00000395117	1.3913753	0.008994	0.049082
RBBP5	NM_005057.2	-0.5295984	0.027751	0.035593
RBBP6	NM_032626.5	-0.1387389	0.028499	0.00977
RBM38	NM_017495.4	0.6197177	0.009393	0.038522
RBM4	BC021120.1	1.302791	0.090328	0.04897
RBM41	NM_018301.2	0.8829134	0.002491	0.018002
RBM6	BC046643.1	0.5353827	0.014055	0.020213
RBMY2FP	BC026077.2	0.4505665	0.034644	0.024277
RCAN3	BC035854	0.3277612	0.009853	0.040249
RDH10	NM_172037.2	-0.4488639	0.00119	0.00389
RDH11	BC051291.1	0.4413108	0.048532	0.011157
RDH11	JHU07368	-0.6884218	0.032533	0.02314
RDH13	NM_138412.2	-1.3583544	0.009451	0.023693
REC8	BC004159.2	0.1539872	0.011713	0.006841
REEP1	NM_022912.1	-0.795501	0.01294	0.02475

RELB	NM_006509.2	1.2885636	0.006891	0.035223
RELL2	BC063469.1	1.4660568	0.035084	0.023935
REM1	NM_014012.4	-0.8636629	0.08522	0.032886
RERGL	NM_024730.2	-1.6936577	0.008657	0.020345
RET	JHU08904	-0.0700989	0.031226	0.00786
RFFL	BC028424.1	-0.4772339	0.057624	0.029187
RFT1	NM_052859.2	0.8657587	0.004417	0.029978
RFX4	BC028582.2	-0.3478216	0.02211	0.023023
RGL4	BC101108.3	0.5183141	0.007109	0.038258
RGMA	BC015886.1	1.9561125	0.012281	0.040468
RGS2	NM_002923.1	-0.8468585	0.029135	0.036499
RGS20	NM_003702.2	-0.2232913	0.018183	0.034041
RGS8	NM_033345.2	1.7047444	0.007591	0.04821
RHBDD1	JHU15746	0.0139703	0.014806	0.032914
RHBDD1	NM_032276.2	-0.4619789	0.046798	0.005141
RHBDF2	BC016034.1	1.2893023	0.006988	0.038259
RHOT1	NM_001033566.1	0.0708674	0.005649	0.01905
RHOT2	NM_138769.1	-0.8120243	0.00761	0.045262
RIBC1	NM_001031745.1	-0.8036115	0.036789	0.046542
RING1	NM_002931.3	0.3641554	0.004696	0.029178
RIOK1	NM_031480.2	-0.8034896	0.017039	0.033004
RIOK3	NM_003831.2	-1.4219617	0.030744	0.013373
RIPK2	JHU00074	0.5492215	0.002068	0.026719
RIPK2	JHU00074	-0.5704741	0.016995	0.002534
RIPK4	BC035755	0.38251	0.005308	0.011244
RIT1	NM_006912.4	-0.2919152	0.020071	0.043092
RNASE2	NM_002934.2	-1.0358921	0.031576	0.048645
RND3	JHU14238	-0.9989822	0.00166	0.014481
RNF113A	NM_006978.1	-1.0128236	0.01061	0.003525
RNF149	BC019355	0.1606902	0.004098	0.018416

RNF166	NM_178841.2	0.1564084	0.049571	0.044141
RNF182	NM_152737.1	0.7938405	0.006554	0.018636
RNF20	NM_019592.5	-1.1272401	0.004464	0.012533
RNF215	NM_001017981	0.4878429	0.00172	0.005152
RNF32	BC028120.1	2.6730455	0.011329	0.028643
RNF6	NM_005977.2	0.7605368	0.013542	0.032918
RNF7	BC005966.1	0.5072572	0.00701	0.012779
ROCK2	NM_004850.3	0.7677774	0.001362	0.011034
RPAIN	BC004451.1	-0.0103598	0.01924	0.047038
RPAP1	BC000246.1	1.0687654	0.029117	0.035915
RPH3AL	NM_006987.2	0.1799856	0.01134	0.041253
RPL10	BC071918.1	0.1397356	0.007489	0.026591
RPL13A	NM_012423.2	-2.1251153	0.0098	0.011819
RPL23A	NM_000984.5	1.5563256	0.026355	0.034267
RPL32P3	BC053996.1	0.0762944	0.010499	0.049431
RPL35A	NM_000996.2	-2.1522913	0.018855	0.049569
RPL36AL	NM_001001.3	-0.2821869	0.024234	0.035996
RPL7	BC009599.1	0.4515539	0.010795	0.046496
RPL7L1	NM_198486.2	0.0939448	0.009138	0.024238
RPN1	NM_002950.3	0.7218849	0.019239	0.044542
RPRD1A	BC010136.2	0.163059	0.039039	0.026606
RPS2	NM_002952.3	0.3227625	0.022268	0.032474
RPS20	NM_001023.2	-0.1347962	0.003651	0.048301
RPS21	ENST00000370562	0.5960221	0.006024	0.016839
RPS24	NM_033022.2	1.2810792	0.061319	0.034013
RPS27A	NM_002954.3	-0.0469379	0.094149	0.037872
RPS6KA4	BC047896.1	1.0640097	0.007022	0.034339
RPS6KB1	NM_003161.2	-1.6357254	0.007362	0.039451
RPS9	NM_001013.3	0.6764406	0.016467	0.029859
RPSA	BC050688.1	-0.3492381	0.031615	0.005322

RRM2	NM_001034.1	0.5019776	0.01957	0.00659
RRP12	JHU12704	-1.9076452	0.004888	0.003674
RRP36	NM_033112.2	-0.7198785	0.000846	0.010445
RSAD2	BC017969.1	0.036552	0.020626	0.035822
RSG1	NM_030907.2	1.0342837	0.012797	0.033578
RSPH10B2	BC044242.1	0.197696	0.036785	0.029816
RSRC1	BC010357.1	0.1927844	0.079336	0.037913
RTCA	BC012604.1	-1.7849689	0.06618	0.043144
RTFDC1	BC003359.1	1.0586971	0.01005	0.037146
RTN3	NM_006054.2	2.3519295	0.024637	0.0264
RTN4	NM_153828.2	-0.7044534	0.007223	0.009613
RTP4	BC013161.1	-0.9259064	0.061539	0.01337
RTP5	JHU29113	1.4784581	0.009577	0.036554
RUNDC1	NM_173079.1	-0.0268338	0.005868	0.00963
RWDD4	BC017472.1	-0.7953854	0.013889	0.010589
RYK	NM_001005861	0.4286715	0.005341	0.008149
RYK	RYK	-1.0676091	0.012218	0.025751
S100A3	NM_002960.1	-1.0163794	0.081343	0.030715
SAP18	NM_005870.3	0.6950446	0.016079	0.034646
SAP18	BC030836.1	0.2433585	0.005905	0.028651
SAP30	NM_003864.3	0.9913242	0.017908	0.038599
SARNP	NM_033082.1	1.7639212	0.008209	0.011745
SARS	BC009390.2	-0.2245184	0.005936	0.046671
SAT2	NM_133491.2	0.1026696	0.007143	0.042395
SBF2	BC011742.2	-0.0432618	0.007714	0.039452
SCARB1	Q8WTV0	0.8781008	0.002922	0.024198
SCCPDH	BC026185.2	0.1787248	0.006251	0.000605
SCD	NM_005063.4	0.5102107	0.013907	0.012129
SCML1	NM_001037535.1	-0.6529724	0.015892	0.002548
SCN2B	NM_004588.3	1.0170263	0.010312	0.002716

SCN4B	BC035017.2	0.8295511	0.021341	0.023978
SCNN1D	NM_002978.2	-1.3180747	0.003668	0.048016
SCO1	JHU05257	0.8282572	0.021222	0.036351
SCO1	NM_004589.1	-1.9665245	0.02496	0.045809
SCP2	NM_001007250.1	-0.376112	0.006747	0.004681
SCRN3	JHU29119	0.5456848	0.007879	0.019574
SCUBE1	NM_173050	-0.399444	0.046602	0.018375
SDC4	NM_002999.2	1.6279107	0.003503	0.011614
SDE2	BC071563.1	1.0139524	0.007114	0.026627
SDF4	NM_016176.2	-0.6614694	0.055679	0.038638
SDSL	NM_138432.2	0.2063678	0.003106	0.028895
SEC22C	JHU08999	0.1403595	0.008066	0.047973
SEC31A	BC047883.2	1.6193273	0.016701	0.036811
SEL1L3	NM_015187.1	-1.7332066	0.003045	0.006934
SEMA3F	NM_004186.2	0.3107096	0.015059	0.011722
SEP15	NM_004261.3	-2.4289156	0.036398	0.041672
SEPHS2	NM_012248.2	0.7284273	0.020151	0.024166
SEPT2	JHU07584	0.2448016	0.006542	0.006747
SEPT2	NM_001008491.1	-0.1182904	0.061296	0.006757
SEPT6	NM_145799.2	1.7537113	0.016165	0.024453
SEPT6	BC009291.2	-0.2741856	0.00673	0.047495
SERF1A	NM_021967.1	1.12307	0.010977	0.047224
SERF1A	JHU03893	0.0191855	0.011922	0.012871
SERHL2	BC041802.1	-1.3921525	0.065575	0.028734
SERPINA4	NM_006215.2	1.1390312	0.010374	0.000277
SERPINA7	BC020747.2	1.1549572	0.009206	0.04767
SERPINB2	BC012609.1	1.4070915	0.006301	0.038826
SERPINE2	JHU07659	1.8071705	0.010806	0.040128
SERTAD4	NM_019605.2	-0.8683065	0.027086	0.003713
SETDB1	BC009362.2	0.1152875	0.010119	0.010394

SFRP4	BC058911.1	0.8360802	0.037903	0.003112
SFRP5	NM_003015.3	1.7075756	0.005401	0.03095
SFT2D2	NM_199344.1	-0.5531127	0.013781	0.005658
SFXN3	ENST00000393459	0.0642878	0.002083	0.014016
SGCD	NM_000337.4	1.3002224	0.012866	0.046986
SGK2	NM_016276.3	0.2743719	0.01852	0.038146
SGOL1	NM_138484.1	2.4823162	0.013695	0.000796
SH3BGR	BC006371.2	0.9706049	0.005769	0.001711
SH3BGRL	NM_003022.1	-1.0256957	0.007304	0.039026
SH3PXD2A	NM_014631	0.9699488	0.008172	0.032528
SH3YL1	JHU03145	-1.0965053	0.029379	0.040653
SHANK2-AS3	BC004224.2	-1.5349185	0.011988	0.026694
SHISA6	NM_207386	0.3120614	0.00881	0.049061
SHKBP1	NM_138392.1	1.4553633	0.008802	0.002775
SIGLEC5	BC029896.1	0.5574448	0.011103	0.025319
SIM2	BC110444	0.7491855	0.02808	0.037775
SIN3B	BC063531.1	-0.2394038	0.005904	0.005491
SIPA1L2	BC013119.1	0.3479532	0.012082	0.05
SIRPB1	BC075835.1	-0.7223568	0.003816	0.026122
SIRT3	NM_012239.4	1.1145673	0.01145	0.019618
SIX4	BC098282	-0.4094979	0.037055	0.015356
SKAP1	Q86WV1	-0.3141233	0.006072	0.009303
SKAP1	JHU07660	-0.3712819	0.037497	0.023899
SKAP2	JHU00079	-1.1327372	0.110824	0.005874
SKP1	NM_006930.2	0.7043343	0.003218	0.042698
SKP1	NM_170679.1	0.1174751	0.020748	0.020363
SLC10A4	NM_152679.2	0.6844438	0.003923	0.027628
SLC13A4	BC030689	0.1939306	0.021515	0.017326
SLC15A2	NM_021082.2	0.956271	0.003331	0.017167
SLC16A5	NM_004695.2	-0.1507866	0.005454	0.027544

SLC1A1	BC033040.1	-1.238923	0.012557	0.002745
SLC1A6	BC028721.2	1.0768675	0.005362	0.036833
SLC22A10	NM_001039752	0.274535	0.003239	0.040432
SLC22A14	SLC22A14	-0.8138193	0.016532	0.017794
SLC22A23	NM_021945.4	-0.2444835	0.009653	0.004186
SLC22A9	ENST00000310969	-2.573966	0.018782	0.040016
SLC23A1	NM_005847.3	1.2224817	0.009205	0.048244
SLC25A15	NM_014252.2	0.8169829	0.016729	0.044781
SLC25A37	SLC25A37	0.138591	0.008738	0.029238
SLC26A2	BC059390.1	1.6987096	0.00687	0.007905
SLC27A5	JHU17487	-0.1682114	0.03126	0.025189
SLC2A12	NM_145176.2	-0.0518185	0.018867	0.01072
SLC30A5	NM_022902.2	-0.1862171	0.014378	0.03971
SLC30A6	NM_017964.2	0.2250369	0.004168	0.048921
SLC30A6	BC032525.1	-1.2893893	0.006306	0.036484
SLC30A9	BC007732.2	1.2536721	0.01402	0.025577
SLC35A5	NM_017945.2	0.4802296	0.03873	0.043129
SLC35B1	NM_005827.1	0.4063005	0.015653	0.011655
SLC35C1	NM_018389.3	0.4595115	0.027795	0.001485
SLC35E3	NM_018656.2	2.4318789	0.031587	0.049437
SLC35G2	BC000111.1	1.6415944	0.010627	0.012413
SLC36A3	BC101094.1	0.4225473	0.005571	0.009945
SLC36A4	BC047374.1	1.6758454	0.015488	0.025964
SLC38A10	NM_138570.2	0.356	0.018952	0.031646
SLC41A3	BC035753.1	-0.7721173	0.057058	0.039909
SLC43A1	NM_003627.4	-0.162291	0.013455	0.048028
SLC43A3	NM_014096.2	-0.717839	0.02132	0.042361
SLC46A3	JHU09967	1.5300602	0.005071	0.017515
SLC52A1	BC092473.1	1.1493388	0.003555	0.018955
SLC5A1	JHU18813	1.2843369	0.00154	0.01482

SLC5A10	BC039868.1	0.3857042	0.041518	0.042867
SLC5A12	JHU05747	0.6167559	0.004149	0.018808
SLC5A7	NM_021815.2	0.4094894	0.054078	0.0194
SLC6A11	BC036083.1	0.9667738	0.065477	0.006315
SLC6A13	NM_016615.2	0.6283158	0.004151	0.021805
SLC9A1	SLC9A1	0.4197091	0.025258	0.048414
SLCO1B3	NM_019844	1.8000222	0.007208	0.041907
SLCO4C1	JHU17490	1.2123341	0.011635	0.048791
SLCO4C1	NM_180991	1.1446785	0.008043	0.020461
SLMO2	NM_016045.2	-0.66207	0.02045	0.048659
SMAD5	NM_001001419.1	1.0676996	0.03149	0.02761
SMAD9	JHU19749	0.5962931	0.009086	0.040183
SMARCA5	BC023144.1	-0.7297667	0.004977	0.026993
SMARCB1	NM_003073.3	0.9673748	0.022282	0.016839
SMIM12	NM_138428.2	1.0892641	0.020317	0.04909
SMIM14	NM_174921.1	1.3515213	0.028253	0.012317
SMIM8	NM_020425.4	0.8948823	0.008724	0.019615
SMN1	JHU03060	1.1072321	0.007049	0.002305
SMOX	NM_175839.1	-0.1198822	0.019409	0.036197
SMPDL3B	NM_001009568.1	1.4511151	0.006035	0.042598
SMYD2	NM_020197.1	0.2078184	0.030527	0.009547
SNAP25	JHU01227	1.4307558	0.00066	0.00232
SNAP91	BC060818.1	-0.7687242	0.008267	0.000239
SNCB	NM_001001502.1	0.8828043	0.041978	0.032716
SNN	NM_003498.4	-1.0675135	0.077269	0.029237
SNPH	NM_014723.2	1.833725	0.029581	0.024675
SNRNP40	NM_004814.2	1.3277305	0.002892	0.018193
SNTB2	ENST00000360496	1.077175	0.010886	0.030516
SNX13	JHU07662	-1.8792052	0.03959	0.040019
SNX24	NM_014035.1	-0.4519417	0.01969	0.041016

SNX33	NM_153271.1	-0.8492865	0.008034	0.016596
SOAT2	BC096090.1	0.3674203	0.005246	0.039342
SOGA3	NM_001012279	1.0946933	0.009832	0.036941
SORBS3	NM_001018003.1	0.3573654	0.031555	0.001483
SOX8	NM_014587.2	0.1498696	0.02871	0.025387
SP5	SP5	1.9668524	0.00418	0.008622
SPANXB1	NM_032461.2	-0.4476686	0.013224	0.020727
SPANXN4	NM_001009613.1	2.0185299	0.009117	0.035421
SPARCL1	BC033721.1	0.2525211	0.009088	0.040901
SPATA16	BC034496.2	-1.3078691	0.003789	0.005671
SPATA20	NM_022827.2	-0.7506296	0.014822	0.043911
SPATA5L1	BC051861.2	0.8470104	0.016056	0.045008
SPATA6	BC020660.1	1.9662578	0.003479	0.019042
SPATC1L	JHU01557	0.6172611	0.009932	0.023308
SPATS2L	BC018736.1	2.5611011	0.008974	0.019572
SPCS2	NM_014752.1	-1.8580807	0.035989	0.014834
SPCS3	NM_021928.1	-0.0492299	0.032315	0.049023
SPEF1	BC022476.1	0.0492782	0.017417	0.022968
SPEG	NM_005876.3	1.4210906	0.014791	0.04768
SPG7	NM_003119.2	1.2155866	0.043576	0.038627
SPHKAP	SPHKAP	0.1501687	0.018299	0.024567
SPINK14	NM_001001325.1	0.0378449	0.004194	0.029359
SPINK2	NM_021114.1	0.8154895	0.008572	0.017669
SPINK6	BC032003.1	-0.5306761	0.054771	0.02509
SPINT2	BC011951.1	-2.2709165	0.029641	0.040978
SPPL2B	BC001788.1	-0.7326059	0.027051	0.036223
SPRY2	NM_005842.2	-0.5389688	0.021353	0.042033
SPSB4	NM_080862.1	0.1462249	0.000806	0.009889
SQLE	NM_003129.3	-0.2550361	0.042158	0.049897
SRA1	BC067895.1	0.1407822	0.009939	0.030031

SRD5A1	NM_001047.2	0.7956951	0.003597	0.01043
SRFBP1	NM_152546.2	1.8064051	0.003867	0.019783
SRL	NM_001098814	0.5569172	0.003064	0.036732
SRMS	JHU19242	1.8553188	0.007953	0.024143
SRMS	NM_080823	1.3252032	0.00869	0.000786
SRP54	NM_003136.2	1.1747132	0.046028	0.046943
SRP72	NM_006947	-0.3018988	0.004662	0.031311
SRPX2	JHU13675	2.1371897	0.014466	0.041787
SRR	NM_021947.1	0.8804363	0.002466	0.01003
SRSF2	NM_003016.2	1.5192559	0.032139	0.019269
SRSF3	BC000914.1	-1.2154513	0.031781	0.045863
SS18L2	NM_016305.2	0.819725	0.045085	0.041515
SSBP1	BC093054.1	1.3028846	0.028992	0.033503
SSBP1	NM_003143.1	0.6612747	0.055255	0.005773
SSX4	BC005325	1.2585187	0.013814	0.027958
ST6GAL2	BC008680.1	2.7863027	0.011645	0.048093
ST6GALNAC1	BC022462.1	-1.1216942	0.044899	0.038943
ST6GALNAC4	NM_175040.3	1.0313959	0.006275	0.021111
STAC	BC020221.1	0.4848342	0.026074	0.006681
STAC3	NM_145064.1	0.8097038	0.029628	0.041482
STAMBP	NM_006463.3	1.7743678	0.045101	0.02274
STARD3	JHU07665	-0.2783437	0.026505	0.046526
STAT4	BC031212.1	0.6953106	0.01009	0.010311
STAU2	NM_014393.1	0.4276313	0.014272	0.022913
STEAP3	NM_001008410.1	-1.2942527	0.019539	0.044157
STK11IP	BC034051.1	0.0949768	0.018189	0.017981
STK3	JHU07093	-1.0738975	0.003165	0.032706
STK35	BC140884.1	-0.2492348	0.013107	0.047148
STOX1	NM_152709	0.0972325	0.030048	0.030013
STRA13	BC009571.1	-1.6374417	0.013732	0.01348

STRADB	NM_018571.4	1.4386385	0.043925	0.013076
STS	NM_000351.3	1.075602	0.011601	0.040597
STX11	NM_003764.2	-0.9564001	0.007618	0.033441
STXBP2	NM_006949.1	0.1814119	0.001749	0.03904
STXBP5	NM_139244.2	0.1201345	0.019285	0.030943
SUB1	NM_006713.2	-0.0777687	0.076174	0.046569
SUDS3	BC093990.1	0.1666959	0.007367	0.032077
SULT2A1	BC020755	-0.0462939	0.009984	0.046401
SULT2B1	NM_177973.1	1.0859739	0.020917	0.006277
SUMF2	NM_001042469.1	1.0330173	0.01543	0.032988
SUMO4	NM_001002255.1	-0.0378313	0.006249	0.037567
SUPT3H	BC050384	1.0828581	0.005375	0.03277
SUPV3L1	NM_003171.2	-1.8867518	0.009128	0.018635
SURF1	NM_003172.2	-0.8927805	0.013902	0.001558
SUSD3	NM_145006.2	-0.4605009	0.003383	0.032501
SUV420H1	BC098121.1	1.1975681	0.028665	0.01237
SYNCRIP	BC032643.1	1.7259354	0.008342	0.000254
SYNGAP1	NM_006772	0.47038	0.017478	0.02482
SYT16	BC040924.1	0.907172	0.069282	0.024908
SYT6	NM_205848.2	-0.0769053	0.024911	0.031932
SYTL2	NM_206928.1	0.8439879	0.005559	0.023596
TAAR6	NM_175067.1	1.3778712	0.035348	0.028761
TAB1	NM_006116.2	-0.0135412	0.000942	0.005689
TAC4	NM_001077504.1	0.1203752	0.002383	0.020872
TACR1	NM_015727.1	-1.3101031	0.039463	0.034619
TAF10	NM_006284.2	1.0942551	0.011051	0.029297
TAF11	NM_005643.2	2.3797175	0.045365	0.044545
TAF1D	NM_024116.2	0.5209296	0.014069	0.012641
TAOK3	NM_016281.2	1.4426461	0.006636	0.007776
TAP2	ENST00000383118	0.1039368	0.010181	0.037027

TARBP1	NM_005646	0.1431033	0.011322	0.021561
TAS2R14	JHU08516	0.018936	0.025351	0.029931
TAS2R40	NM_176882.1	0.8846683	0.018825	0.021014
TAS2R5	BC095522.1	0.0627229	0.045154	0.031713
TAX1BP1	BC050358.2	-0.2497339	0.024053	0.006379
TBC1D20	NM_144628.2	1.1748876	0.051795	0.019028
TBC1D4	NM_014832	1.3221246	0.005664	0.038255
TBC1D9B	NM_015043.3	1.1725494	0.008174	0.045314
TBCA	NM_004607.2	0.8450624	0.004255	0.010765
TBPL1	BC000381.2	0.7079666	0.008544	0.04952
TBX10	TBX10	1.1714798	0.006643	0.040999
TBX18	TBX18	1.3451582	0.008829	0.03262
TCAP	BC013330.1	0.9820488	0.013249	0.011098
TCEA2	NM_198723.1	-0.6801843	0.007743	0.039549
TCEA2	BC050623.1	-1.3681251	0.012426	0.049593
TCEAL5	NM_001012979.1	-0.3245158	0.003227	0.002206
TCEANC2	NM_153035.1	1.0236402	0.018183	0.010401
TCEB2	NM_207013.1	0.3158008	0.005117	0.004377
TCEB3B	BC103911.2	1.0324196	0.017528	0.018349
TCERG1L	BC093639.1	-0.229661	0.033072	0.029311
TCN1	BC018632.1	-0.1067672	0.003791	0.040378
TCN2	BC001176.1	2.016107	0.013131	0.00446
TCN2	NM_000355.2	-1.0728099	0.048113	0.005674
TCOF1	NM_001008657.1	0.4093803	0.012396	0.005988
TCTA	NM_022171.1	1.83391	0.021914	0.00414
TECPR1	NM_015395.1	1.7776781	0.036109	0.004045
TERT	NM_198253.2	-0.308434	0.006312	0.021722
TESK2	BC033085.1	0.6971486	0.008335	0.019114
TET2	XM_042301.6	2.338843	0.002269	0.000497
TEX101	BC001861.1	-1.1820864	0.020734	0.001631

TFAP2B	BC037225.1	0.6010773	0.011532	0.031858
TFAP4	NM_003223.1	-0.6408518	0.003834	0.000883
TFB2M	NM_022366.1	0.8070381	0.06675	0.044861
TFB2M	JHU06329	0.6766468	0.007895	0.030255
TFF2	NM_005423.3	-0.2871836	0.003734	0.030237
TFG	NM_001007565.1	0.6283736	0.005977	0.02754
TFPI2	BC005330.1	-0.3601567	0.024222	0.03697
TGFA	BC005308.1	0.9929762	0.010553	0.04272
TGFA	NM_003236.1	0.6235845	0.010972	0.04772
TGFB3	NM_003239.2	0.2571551	0.012261	0.010008
THAP1	NM_018105.2	-0.7211489	0.016534	0.035231
THAP10	NM_020147.2	-0.6946716	0.02485	0.038549
THAP9	NM_024672	1.8435022	0.012487	0.047536
THEM5	NM_182578.1	0.4671443	0.002343	0.020445
THOC3	BC066325.1	0.4147793	0.020699	0.025449
THOC7	JHU12045	0.9702603	0.0081	0.037626
THOC7	BC065012.1	-0.7017224	0.044946	0.024978
THUMPD3	BC001622.1	1.326173	0.004161	0.00205
THY1	AL161958	0.7986675	0.005406	0.030012
TIMM17B	NM_005834.1	-1.0310553	0.02988	0.036422
TIMM23	NM_006327.2	0.3767294	0.002603	0.012034
TIMM8A	NM_004085.2	-0.4472262	0.013992	0.041777
TIMMDC1	NM_016589.3	0.7520789	0.005422	0.005969
TINAG	NM_014464.2	0.613487	0.004127	0.019524
TINF2	BC005030.1	1.3655099	0.018489	0.001178
TIPIN	JHU06997	-0.6311898	0.001844	0.022451
TLK1	TLK1	1.020847	0.0053	0.035858
TLX1	TLX1	1.5080677	0.005227	0.037393
TM2D3	BC008873.2	-0.2460347	0.015298	0.009078
TM4SF1	NM_014220.2	0.5448273	0.061929	0.029988

TM4SF18	NM_138786.1	-1.9618428	0.02391	0.012697
TM7SF2	BC012857.1	-0.5671944	0.008879	0.042778
TMA16	BC011842.2	-1.3836034	0.012852	0.041769
TMBIM6	BC000916.1	-0.8950043	0.050893	0.024406
TMCO1	NM_019026.2	-1.5658479	0.028354	0.035982
TMCO4	BC053600.1	-0.4984637	0.010509	0.044625
TMCO6	BC001910.1	1.124872	0.017132	0.006998
TMED1	NM_006858.2	-1.4134113	0.026539	0.026153
TMED2	NM_006815.2	1.0943098	0.00789	0.046788
TMED4	NM_182547.2	-1.1215688	0.022314	0.005466
TMED5	NM_016040.3	-0.2043705	0.004852	0.004813
TMEM110	NM_198563.1	0.5901113	0.014295	0.00415
TMEM120A	BC051850.1	0.2539835	0.004425	0.004062
TMEM14B	NM_030969.2	0.8416677	0.006425	0.042752
TMEM167B	NM_020141.3	-1.3015011	0.024369	0.006386
TMEM174	NM_153217.1	1.6429768	0.011076	0.008797
TMEM18	BC032379.1	1.8161574	0.037608	0.000829
TMEM187	NM_003492.1	0.7485601	0.003147	0.019711
TMEM200A	NM_052913.2	0.3772144	0.017156	0.010566
TMEM208	BC013412.2	1.8188814	0.004217	0.020208
TMEM209	BC057784.1	1.2785997	0.019342	0.003087
TMEM211	NM_001001663.1	-0.2869457	0.004907	0.026934
TMEM217	BC029657.1	0.6406868	0.021855	0.03857
TMEM239	BC021178.2	-2.3044116	0.058868	0.041303
TMEM27	NM_020665.2	-0.9765976	0.008763	0.026361
TMEM5	BC013152	-0.546683	0.024244	0.011907
TMEM52B	BC098571.1	-2.1830103	0.01379	0.027897
TMEM59	NM_004872.3	-1.3700708	0.031872	0.038706
TMEM81	NM_203376.1	-1.67658	0.043447	0.044071
TMOD1	NM_003275.1	1.150736	0.007822	0.045223

TMPO	NM_001032283.1	0.3446268	0.008756	0.010818
TMPRSS12	BC035123.1	-1.9019952	0.066464	0.031601
TMPRSS5	NM_030770.1	-0.4963527	0.032024	0.040942
TMSB4X	NM_021109.2	0.338059	0.009155	0.004065
TMUB1	NM_031434.2	1.6039711	0.003495	0.01798
TNFRSF10B	NM_147187.1	-0.8279845	0.009492	0.022328
TNFRSF21	BC010241.1	0.2803554	0.031564	0.028891
TNFRSF25	TNFRSF25	1.3106537	0.004946	0.027932
TNFSF12	BC071837.1	-0.1887461	0.001064	0.013147
TNFSF15	NM_005118.2	-0.4592615	0.020822	0.015691
TNIP1	BC007666.2	0.3868729	0.039839	0.004794
TNKS2	NM_025235	-1.1154305	0.014223	0.018861
TOR1AIP2	NM_145034.1	0.3708535	0.012364	0.049528
TP53	BC003596.1	-0.5066263	0.062379	0.044576
TP53RK	BC066309.1	-1.8973058	0.038883	0.016502
TP63	NM_003722.3	1.1100966	0.004536	0.005196
TPD52L1	NM_001003396.1	-0.5748268	0.039465	0.003068
TPD52L3	NM_001001874.1	-0.6076901	0.020874	0.036897
TPMT	BC005339.1	0.6467371	0.00216	0.001922
TPSB2	JHU13777	0.8624963	0.013464	0.049227
TPST1	NM_003596.2	-0.7346443	0.011805	0.000806
TPT1	BC022436.1	0.7370544	0.019223	0.034135
TPT1	NM_003295.1	-0.6567601	0.047113	0.028837
TPTE2	NM_199255.1	-0.0167254	0.020044	0.046442
TRAC	JHU01914	2.3813337	0.015615	0.012135
TRAF3IP3	TRAF3IP3	0.5900278	0.007928	0.032714
TRAF7	BC024267.1	-1.5703367	0.016426	0.043032
TREM1	NM_018643.2	0.8663077	0.035703	0.021786
TRGC1	JHU16241	-0.0014982	0.005344	0.022687
TRIM31	BC016866.1	0.0887783	0.00297	0.032052

TRIM32	BC003154.1	1.6159662	0.005556	0.025764
TRIM46	BC069416.1	0.7459361	0.008591	0.001488
TRIM48	NM_024114.2	1.3076721	0.005132	0.009625
TRMT1	BC018302.1	0.0772725	0.009961	0.028725
TRMT10B	NM_144964.2	-1.7556837	0.009437	0.034488
TRMT1L	BC045535.1	-0.1379563	0.007455	0.031305
TRMT44	NM_152544.1	0.6013382	0.008501	0.008738
TRMT6	NM_015939.3	2.7552104	0.009884	0.022641
TRNAU1AP	NM_017846.4	0.7652095	0.001262	0.002792
TROAP	BC032583.1	1.1595999	0.020227	0.016514
TRPM3	NM_001007470.1	-0.1293571	0.014539	0.012984
TRPV6	NM_018646	1.1491	0.011535	0.038434
TSC22D3	NM_001015881.1	-0.0441026	0.01511	0.019215
TSG101	BC002487.1	-0.8627695	0.01869	0.047576
TSHB	NM_000549.3	1.4845118	0.009155	0.001262
TSSC4	BC050616.1	-1.1408846	0.003726	0.035357
TTC16	BC031281.1	0.8398819	0.013679	0.021581
TTC27	NM_017735.3	0.4718449	0.011247	0.009659
TTC30B	BC033795.1	-0.8463969	0.026145	0.048678
TTC39B	BC038592.1	0.5319154	0.008488	0.021893
TTI2	BC007387.2	0.728943	0.016827	0.024709
TTLL3	BC009479.1	0.597955	0.018226	0.032317
TUBA3C	NM_079836.1	0.3196343	0.003572	0.022911
TUBB2B	NM_178012.3	-0.9270048	0.012334	0.048906
TUBGCP5	NM_052903.2	1.1528001	0.013282	0.023578
TULP1	BC032714.1	1.1502024	0.007909	0.007365
TULP3	BC032587.1	0.1978165	0.009103	0.044858
TUSC5	NM_172367.1	0.098548	0.003981	0.035035
TWF1	NM_002822.3	1.170541	0.004943	0.007499
TWIST1	NM_000474.3	-1.2371713	0.024741	0.014735

TWSG1	NM_020648.3	1.0509901	0.048098	0.036265
TXK	NM_003328	1.9462618	0.008343	0.029565
TXN	NM_003329.2	-1.461537	0.009177	0.023698
TXNDC15	BC001615	0.0604497	0.002922	0.012401
TXNDC16	NM_020784.1	2.8100157	0.012974	0.047317
TXNDC8	NM_001003936.1	0.38746	0.029152	0.048538
TXNIP	NM_006472.1	0.9000705	0.006587	0.011226
TYRO3	NM_006293.2	-0.7710806	0.007756	0.025648
UBASH3B	NM_032873.3	-0.7092006	0.013118	0.045083
UBE2C	BC016292.1	0.4406385	0.002109	0.021073
UBE2F	NM_080678.1	-0.6051904	0.025905	0.023339
UBE2H	NM_003344.2	0.2804819	0.00073	0.026486
UBE2J2	ENST00000358663	1.4673383	0.014394	0.020578
UBE2L3	JHU06330	-2.5802358	0.032046	0.001826
UBE2Q1	BC015316.1	0.0862695	0.004558	0.044283
UBE2V1	NM_022442.3	0.388065	0.014813	0.009537
UBE3A	BC002582.2	0.6236949	0.003243	0.024483
UBE3C	BC026241.1	0.361468	0.013103	0.03968
UBL5	NM_024292.2	0.9325383	0.005968	0.028968
UBP1	JHU05845	1.19844	0.00919	0.018341
UBP1	NM_014517.2	1.1939854	0.005809	0.04005
UBQLN2	NM_013444.2	-0.3544652	0.008999	0.016208
UBQLNL	BC012183.1	0.8832362	0.037941	0.027209
UCHL3	NM_006002.3	1.2481984	0.003371	0.019018
UCMA	NM_145314.1	0.0333595	0.010486	0.007697
UCP3	BC008392.1	0.8964007	0.020113	0.009195
UCP3	NM_003356.2	-1.0145087	0.002242	0.02203
UCP3	NM_022803.1	-1.4861672	0.057392	0.019273
UGDH	NM_003359.2	1.1205919	0.016464	0.018907
UGT1A10	BC053576.1	0.8711859	0.005615	0.03851

UGT1A6	BC019861.1	-1.735931	0.012892	0.028233
UNKL	NM_001037125.1	-0.312833	0.050947	0.016118
Unknown (protein for MGC:88814)	BC073794	1.4839351	0.024368	0.024935
UPK1A	NM_007000.2	-0.1931663	0.028442	0.040086
UQCRC1	NM_003365.2	-1.5234521	0.005004	0.003189
URB2	NM_014777.1	1.0833158	0.009075	0.00501
URM1	NM_030914.1	1.1349984	0.004595	0.029204
USP15	ENST00000312635	1.2984407	0.01142	0.035594
USP18	NM_017414.2	0.7679494	0.006507	0.047889
USP36	BC038983	1.4111546	0.006436	0.015747
USP45	JHU05275	1.3021534	0.006429	0.020404
USP47	BC000226.1	-0.6500681	0.009109	0.035386
UTP18	NM_016001.1	-0.9972877	0.002597	0.042156
VAMP2	JHU04888	1.3989229	0.038946	0.044942
VANGL2	NM_020335.1	-0.9183607	0.008685	0.027401
VASH2	BC053836.1	-1.4188562	0.020101	0.030028
VCAN	JHU07674	0.1859348	0.004808	0.034324
VDAC3	NM_005662.3	0.38968	0.001628	0.011691
VGLL4	BC003038.1	-1.4728374	0.02819	0.047032
VIM	NM_003380.2	0.4789443	0.016391	0.010526
VIP	BC009794.1	-0.8113568	0.011635	0.027122
VMA21	NM_001017980.1	0.6240653	0.02213	0.004151
VOPP1	NM_030796.3	-1.5735009	0.009143	0.012348
VPS39	NM_015289.2	0.4277471	0.004123	0.029628
VSTM2A	NM_182546.1	-1.137456	0.00955	0.033615
VWA2	ENST00000298715	1.1620399	0.017031	0.016794
VWA3B	BC022028.2	-0.2281147	0.056267	0.010806
VWA5A	NM_014622.4	0.878079	0.002019	0.027662
WARS2	NM_201263.1	0.3327515	0.036927	0.000552

WASF3	BC050283.1	0.4245366	0.010689	0.018754
WASH3P	NM_199163.2	-0.3645532	0.069787	0.030633
WBSCR28	NM_182504.2	-0.6386294	0.00902	0.037017
WDR31	NM_001006615.1	2.0503596	0.01861	0.047679
WDR70_frag	JHU12761	-2.1550121	0.011296	0.001866
WDR77	BC009411.1	0.3530972	0.005086	0.046726
WDR88	ENST00000361680	-0.5298743	0.027121	0.01989
WDR91	BC017246.2	0.5231241	0.005303	0.036411
WDTC1	NM_015023.2	0.3214478	0.009813	0.035043
WHAMM	NM_001080435	1.4758972	0.010145	0.002575
WISP2	NM_003881.2	0.1427259	0.003665	0.000675
WNK1	BC021121.1	-1.1331414	0.032938	0.025327
WNT8B	NM_003393.3	0.1797146	0.042929	0.048125
WSCD2	ENST00000261400	0.1034661	0.001598	0.000333
WT1-AS	BC096708.1	1.229331	0.004648	0.015342
WWOX	NM_130844.1	2.023802	0.012175	0.018955
X90000.1_frag	JHU12059	0.3944748	0.004341	0.031602
XDH	NM_000379	0.630082	0.021145	0.035877
XM_001162648.4_frag	JHU12590	-0.6632957	0.007484	0.000492
XM_003830043.3_frag	JHU03847	0.3401404	0.022339	0.039841
XM_006499100.2_frag	JHU19691	1.148972	0.020361	0.028751
XM_006714753.1_frag	JHU11517	0.5172693	0.004713	0.01795
XM_008971841.2_frag	JHU02797	1.2084584	0.042944	0.016983
XM_011510012.1_frag	JHU03575	-0.9430464	0.001359	0.001275
XM_011513424.1_frag	JHU11227	-1.0826888	0.071877	0.01274
XM_011530005.1_frag	JHU11242	0.0406106	0.024737	0.009926
XM_011530315.1_frag	JHU07416	-0.5267928	0.018371	0.027987
XM_014342361.1_frag	JHU02872	0.619407	0.001675	0.005885
XPO4	XPO4	0.4714621	0.016775	0.030394
XR_243401.3_frag	JHU07773	0.0083526	0.008117	0.043771

XR_243654.3_frag	JHU02867	-0.5315727	0.039065	0.015068
XR_432093.2_frag	JHU14999	1.6094179	0.01796	0.045678
XRCC4	BC005259.1	0.3642881	0.010143	0.021003
XRCC4	NM_022406.1	-0.2185715	0.00823	0.032121
XRCC6BP1	NM_033276.2	0.3184652	0.015168	0.045553
YIF1A	BC001299.1	3.0969421	0.020847	0.046555
YIPF6	BC012469	-0.1585882	0.044367	0.032513
YO001	JHU14990	-1.668832	0.009101	0.037759
YS002	JHU19025	-0.8518165	0.029754	0.031146
YS049	JHU03360	-1.6605114	0.00761	0.025146
YT011	JHU17069	0.6311111	0.007435	0.041078
YY2	AY567472.1	-0.4393871	0.00567	0.014152
ZAP70	ENST00000389515	1.6891017	0.013328	0.033666
ZBBX	BC034229.1	0.0440676	0.00494	0.02198
ZBTB22	NM_005453.3	0.2246813	0.04166	0.002864
ZBTB44	BC049375.1	-0.3698207	0.030582	0.046307
ZBTB47	BC021855	0.5012975	0.081629	0.032904
ZCCHC12	BC057841.1	1.0324397	0.026307	0.04786
ZCCHC12	BC036572.2	0.2015777	0.017464	0.036524
ZCCHC3	JHU09982	0.792823	0.005351	0.01599
ZDHHC23	NM_173570.2	-0.4675647	0.023478	0.021712
ZDHHC5	BC026967.1	1.1110916	0.01953	0.024897
ZFAND6	BC005283	0.1329926	0.001892	0.014243
Zfp106	Zfp106	1.3971439	0.005315	0.033162
ZFP36L1	NM_004926.2	-1.7106172	0.037543	0.032605
ZFP64	BC012759.2	-1.2514704	0.019484	0.031207
ZFYVE9	NM_007323.1	1.0559995	0.004975	0.011186
ZG16	NM_152338.1	1.9443298	0.053881	0.001114
ZHX1	NM_001017926.1	2.6173044	0.005106	0.015406
ZHX1-C8orf76	NM_001204180.1	0.3692204	0.00176	0.01504

ZHX3	BC068569.1	0.9236917	0.004518	0.045587
ZKSCAN1	BC022378.1	-0.1436175	0.018025	0.029337
ZKSCAN7	NM_025169.1	-0.8357605	0.079141	0.045329
ZMYND12	NM_032257.3	1.6302172	0.006868	0.011922
ZMYND19	NM_138462.2	0.3915062	0.009318	0.006335
ZNF114	JHU19662	1.3654252	0.015852	0.028783
ZNF136	NM_003437.2	0.0068663	0.003228	0.003842
ZNF137P	NM_003438.2	-0.7505671	0.006957	0.01919
ZNF17	NM_006959	0.7805533	0.02219	0.036864
ZNF174	NM_001032292.1	-0.4214221	0.047958	0.035788
ZNF175	NM_007147.2	-0.6211202	0.011807	0.005351
ZNF177	ENST00000343499	-0.2966421	0.0095	0.001499
ZNF205	ENST00000219091	0.5539084	0.041839	0.014542
ZNF222	BC030261.1	1.015022	0.00886	0.045422
ZNF226	NM_001032372.1	0.7295414	0.017615	0.020697
ZNF23	NM_145911.1	1.1743325	0.007489	0.00521
ZNF24	BC003566.1	0.3850822	0.004738	0.030982
ZNF257	BC036446.1	0.9028737	0.006846	0.016451
ZNF280D	NM_001002844.1	0.4732602	0.001472	0.012583
ZNF284	NM_001037813	0.9405453	0.006093	0.016355
ZNF295-AS1	BC029588.1	0.14828	0.051184	0.027917
ZNF317	BC078154.1	0.0768809	0.014469	0.048232
ZNF320	ZNF320	2.0315666	0.010736	0.004585
ZNF343	ENST00000381253	0.4570533	0.011887	0.008407
ZNF35	ENST00000296092	1.1149332	0.002139	0.016271
ZNF385A	NM_015481.1	1.5568801	0.010569	0.040019
ZNF385C	BC067901.1	0.0956276	0.009515	0.002231
ZNF396	JHU10736	0.6848068	0.004929	0.02606
ZNF408	NM_024741.1	0.2148562	0.014537	0.047302
ZNF423	BC112317	0.9891029	0.015745	0.048399

ZNF426	NM_024106.1	0.9619961	0.006941	0.019167
ZNF431	BC040506.1	1.2249168	0.002091	0.011812
ZNF432	BC002858	1.1317467	0.003837	0.009233
ZNF436-AS1	JHU14527	-0.3070329	0.012144	0.021831
ZNF449	JHU07198	-3.4702449	0.003525	0.01077
ZNF460	ZNF460	1.6332383	0.019485	0.046416
ZNF485	NM_145312.2	-1.1031467	0.008753	0.004242
ZNF497	BC125273	1.5948922	0.015802	0.045832
ZNF501	ENST00000302420	0.4110147	0.016073	0.049569
ZNF507	NM_014910.2	2.2523198	0.005605	0.031879
ZNF524	BC067748.1	-0.9231927	0.01793	0.041562
ZNF526	NM_133444.1	1.2673018	0.007702	0.042894
ZNF528	NM_032423.2	1.0150345	0.006493	0.02706
ZNF540	JHU17207	-0.6820021	0.037322	0.038835
ZNF540	ZNF540	-1.181232	0.000586	0.015697
ZNF584	NM_173548.1	0.3956683	0.032733	0.005132
ZNF585A	BC063820	1.4606231	0.070936	0.023593
ZNF614	ENST00000356322	1.4699246	0.023385	0.031108
ZNF654	NM_018293.1	1.3574507	0.02258	0.04287
ZNF655	NM_001009956.1	-0.0569106	0.003102	0.007589
ZNF658	BC031626.1	-0.1383931	0.003499	0.029101
ZNF669	BC015312.1	-0.8329124	0.006001	0.025751
ZNF671	BC025728.1	1.1311357	0.019546	0.02637
ZNF688	NM_145271.3	-0.6007464	0.012506	0.003619
ZNF691	NM_015911.2	-0.8130696	0.005174	0.026618
ZNF692	NM_017865.2	1.034463	0.003571	0.028122
ZNF71	NM_021216.3	-0.4396784	0.150067	0.030858
ZNF720	BC055408.1	0.7707837	0.00382	0.022017
ZNF746	ZNF746	1.7024877	0.005708	0.03166
ZNF804A	NM_194250.1	1.8413607	0.003483	0.019924

ZNF823	ZNF823	1.7504166	0.008516	0.006037
ZNF839	BC006222.2	-0.1714981	0.01672	0.048739
ZNF85	BC008688.1	-0.5579119	0.001845	0.012247
ZNF92	BC036439.1	1.0079001	0.007823	0.01976
ZNFX1	NM_021035.1	-0.2146229	0.020261	0.044744
ZP2	BC096304.3	0.1167608	0.005822	0.011959
ZP3	NM_007155	0.6880429	0.035968	0.040921
ZSCAN31	NM_145909.1	1.0612856	0.030182	0.001025
ZSCAN5A	NM_024303.1	1.0962704	0.009382	0.046695

Supplementary Table 2. Predominant metabolic pathways enriched for in putative HUWE1 substrates.

**Glycolysis related metabolism**	
SLC2A12	
SLC5A1	
SLC5A10	
ALDH2	
ALDH3A1	
ALDH6A1	
PKM	
**Glutaminolysis Related Metabolism**	
GRM7	Glutamate Receptor 7
SLC1A1	Solute Carrier Family 1 Member 1
SLC1A6	Solute Carrier Family 1 Member 6
SLC38A10	Solute Carrier Family 38 Member 10
GGCT	Gamma-Glutamylcyclotransferase
ODC1	Ornithine Decarboxylase 1
PSAT1	Phosphoserine Aminotransferase 1
**Pentose Phosphate Pathway**	
PGLS	6-Phosphogluconolactonase
PGM2	Phosphoglucomutase 2
**Oxidative Phosphorylation**	
COX7A2L	Cytochrome C Oxidase Subunit 7A2 Like
COX8A	Cytochrome C Oxidase Subunit 8A
COX18	Cytochrome C Oxidase Subunit 18
NDUFA6	NADH:Ubiquinone Oxidoreductase Subunit A6
NDUFAF7	NADH:Ubiquinone Oxidoreductase Assembly Factor 7
NDUFB3	NADH:Ubiquinone Oxidoreductase Subunit B3
**Fatty Acid Related Metabolism**	
SLC27A5	Solute Carrier Family 27 Member 5
FAS	Fatty Acid Synthase
APOA4	Apolipoprotein A4
APOA5	Apolipoprotein A5
ACSL5	Acyl-CoA Synthetase Long Chain Family Member 5
ACACA	Acetyl-CoA Carboxylase Alpha
**Transport ATPases**	
ATP1A1	ATPase Na+/K+ Transporting Subunit A1
APT1A3	ATPase Na+/K+ Transporting Subunit A3
ATP1B3	ATPase Na+/K+ Transporting Subunit B3
APT6V1G3	ATPase H+ Transporting V1 Subunit G3
ATP6V0A2	ATPase H+ Transporting V0 Subunit A2
**Lactic Acid Efflux**	
SLC16A1	Solute Carrier Family 16 Member 1
SLC16A5	Solute Carrier Family 16 Member 5
